# Seven new freshwater species of *Gammarus* from southern China (Crustacea, Amphipoda, Gammaridae)

**DOI:** 10.3897/zookeys.749.23165

**Published:** 2018-04-10

**Authors:** Zhonge Hou, Shuangyan Zhao, Shuqiang Li

**Affiliations:** 1 Key Laboratory of Zoological Systematics and Evolution, Institute of Zoology, Chinese Academy of Sciences, Beijing 100101, China; 2 Southeast Asia Biodiversity Research Institute, Chinese Academy of Sciences, Yezin, Nay Pyi Taw 05282, Myanmar; 3 University of Chinese Academy of Sciences, Beijing, 100049, China

**Keywords:** Cave, DNA barcoding, morphology, new species, taxonomy

## Abstract

Seven new species of the genus *Gammarus* are described and illustrated from southern China. The new species *Gammarus vallecula* Hou & Li, **sp. n.**, *G. qinling* Hou & Li, **sp. n.**, *G. zhigangi* Hou & Li, **sp. n.** and *G. jidutanxian* Hou & Li, **sp. n.** are characterized by inner ramus of uropod III half the length of outer ramus. *Gammarus longdong* Hou & Li, **sp. n.** is characterized by inner ramus of uropod III 0.9 times as long as outer ramus. *Gammarus mosuo* Hou & Li, **sp. n.** is characterized by pereopods V–VII with long setae on anterior margins and both rami of uropod III armed with simple setae. *Gammarus caecigenus* Hou & Li, **sp. n.** can be distinguished from other species by eyes absent. DNA barcodes of the new species are documented as proof of molecular differences between species. A key to the new species and a map of their distributions are provided.

## Introduction

The genus *Gammarus* Fabricius, 1775 contains more than 200 freshwater, brackish, and marine species in the Northern Hemisphere (Väinölä et al. 2008), of which 80% species inhabit fresh waters. They are essential components of freshwater ecosystems, often forming bioindicators for water quality assessment (Gerhardt et al. 2011). Previous studies suggested that *Gammarus* originated from the ancient Tethys, then diversified in Eurasia driven by plate tectonic activities between Eurasia and Africa/India (Hou et al. 2011). In China, 76 species of *Gammarus* have been recorded and phylogenetic analysis indicated that Tibetan uplift triggered the separation of north and south lineages (Hou et al. 2007). The new species described in current paper belong to south lineage.

In the last 15 years several collecting trips were carried out in southern China including Qinling, Daba Mountain, and Yunnan-Guizhou Plateau. This effort allowed the collection of many freshwater *Gammarus*, and was followed by the preliminary description of some new species (Hou and Li 2010, Hou et al. 2013, Li et al. 2013). However, detailed morphological examination and molecular studies of this material revealed a further species diversity that was previously underestimated. In the present paper, seven new species of the genus *Gammarus* from southern China are described and illustrated. A distribution map of new species is presented, as is a key to the new species.

## Materials and methods

### Sampling

The specimens were collected with a fine-meshed hand net. Samples were preserved in 95% ethanol in the field, and then deposited at -20 °C refrigerator for long preservation. Type specimens are lodged in the Institute of Zoology, Chinese Academy of Sciences (**IZCAS**), Beijing.

### Morphological observations

The body length was recorded by holding the specimen straight and measuring the distance along the dorsal side of the body from the base of the first antenna to the base of the telson. All dissected appendages were mounted in glycerol on slides. Appendages were drawing using a Leica DM2500 compound microscope equipped with a drawing tube. Terminology and taxonomic descriptions follow Zhao et al. (2017). The nomenclature of setal patterns on the mandibular palp follows Cole (1980). The holotype specimen was used for morphological observation, while one paratype specimen was used for both morphological and molecular parts.

### DNA sequencing and COI genetic distance calculations

A partial fragment of the mitochondrial cytochrome *c* oxidase subunit I (COI) was proposed as a crustacean barcode (Costa et al. 2007, Hou et al. 2009). The primers used are LCO1490 (5’- GGTCAACAAATCATAAAGATATTGG-3’) and HCO2198 (5’-TAAACTTCAGGGTGACCAAAAAATCA-3’) (Folmer et al. 1994). Genomic DNA extraction, amplification, and sequencing procedures were performed as in Hou et al. (2007). All sequences were deposited in GenBank, and the accession numbers are provided in Table 1.

**Figure 1.**
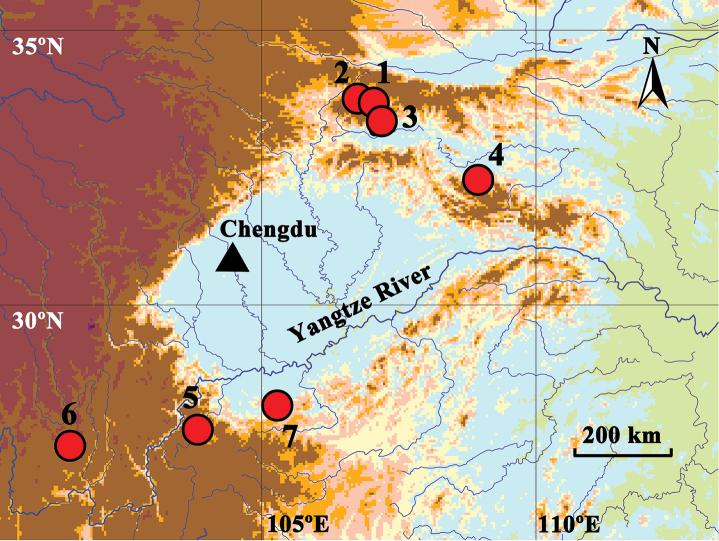
Collection localities of seven *Gammarus* species from southern China. **1** *Gammarus vallecula* Hou & Li, sp. n. **2** *G. qinling* Hou & Li, sp. n. **3** *G. zhigangi* Hou & Li, sp. n. **4** *G. jidutanxian* Hou & Li, sp. n. **5** *G. longdong* Hou & Li, sp. n. **6** *G. mosuo* Hou & Li, sp. n. **7** *G. caecigenus* Hou & Li, sp. n.

**Table 1.** GenBank accession numbers and uncorrected pairwise distance of the COI partial sequences between species in this text.

	Species	GenBank accession number	1	2	3	4	5	6
1	*Gammarus vallecula* Hou & Li, sp. n.	MG550237						
2	*Gammarus qinling* Hou & Li, sp. n.	MG550238	0.177					
3	*Gammarus zhigangi* Hou & Li, sp. n.	MG550239	0.207	0.209				
4	*Gammarus jidutanxian* Hou & Li, sp. n.	MG550240	0.251	0.270	0.243			
5	*Gammarus longdong* Hou & Li, sp. n.	MG550241	0.213	0.202	0.239	0.255		
6	*Gammarus mosuo* Hou & Li, sp. n.	MG550242	0.214	0.208	0.227	0.244	0.227	
7	*Gammarus caecigenus* Hou & Li, sp. n.	MG550243	0.264	0.274	0.275	0.275	0.288	0.254

Raw sequences were edited and assembled using MacClade 4.0 (Maddison and Maddison 2000), and uncorrected pairwise distances between sequences were calculated using MEGA 7.0.16 (Kumar et al. 2016) and are shown in Table 1.

## Taxonomy

### Family Gammaridae Leach, 1814

#### Genus Gammarus

Taxon classificationAnimaliaAmphipodaGammaridae

Fabricius, 1775

##### Type species.

*Gammarus pulex* (Linnaeus, 1758)

#### Gammarus vallecula

Taxon classificationAnimaliaAmphipodaGammaridae

Hou & Li sp. n.

http://zoobank.org/6D34788A-7029-4933-9A27-855786E4F731

**Figure 2.**
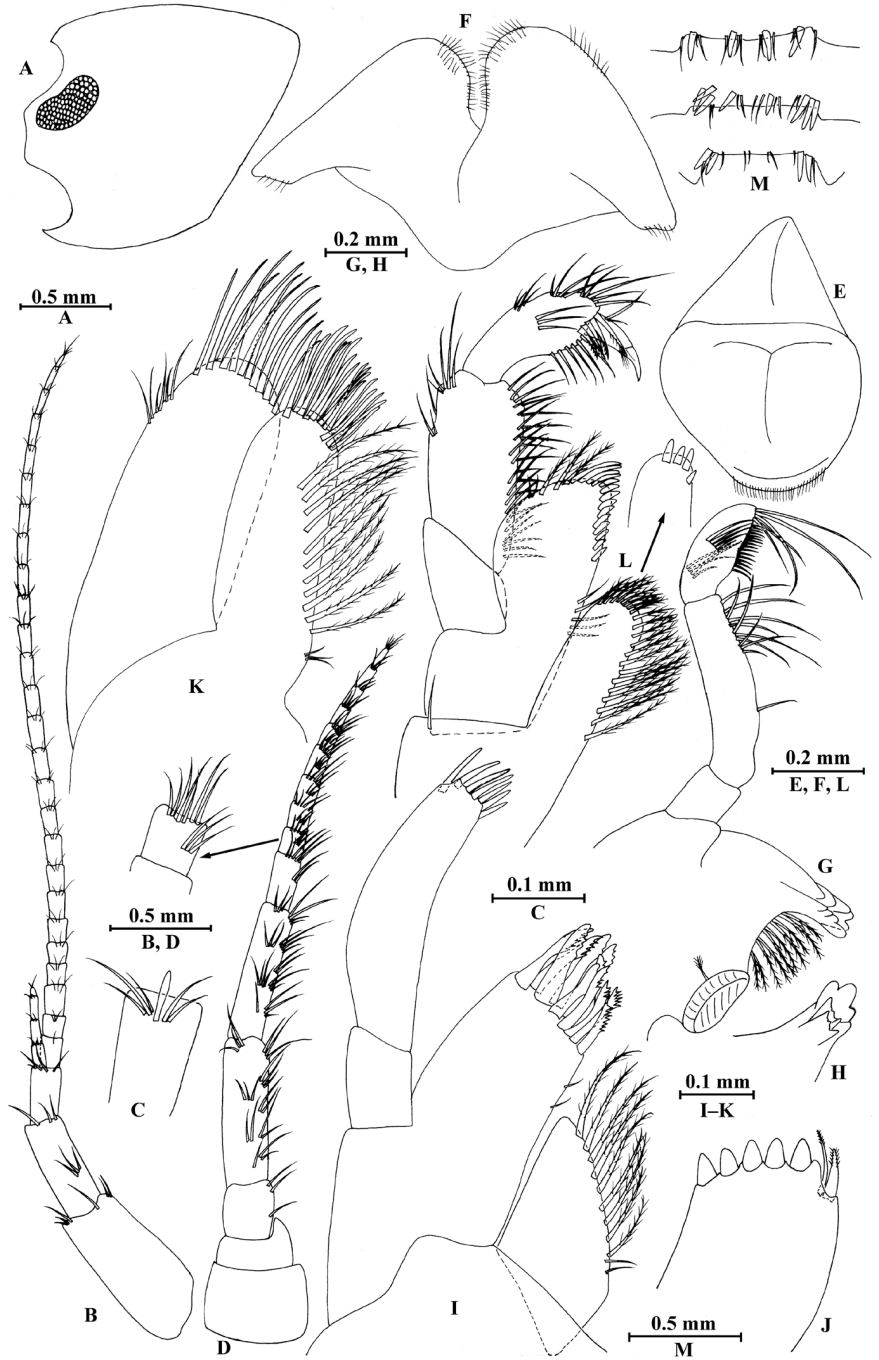
*Gammarus vallecula* Hou & Li, sp. n., male holotype. **A** head **B** antenna I **C** flagellar article of antenna I with aesthetasc **D** antenna II **E** upper lip **F** lower lip **G** left mandible **H** incisor and lacinia mobilis of right mandible **I** left maxilla I **J** distal part of palp article II of right maxilla I **K** maxilla II **L** maxilliped **M** dorsal margins of urosomites I–III.

**Figure 3.**
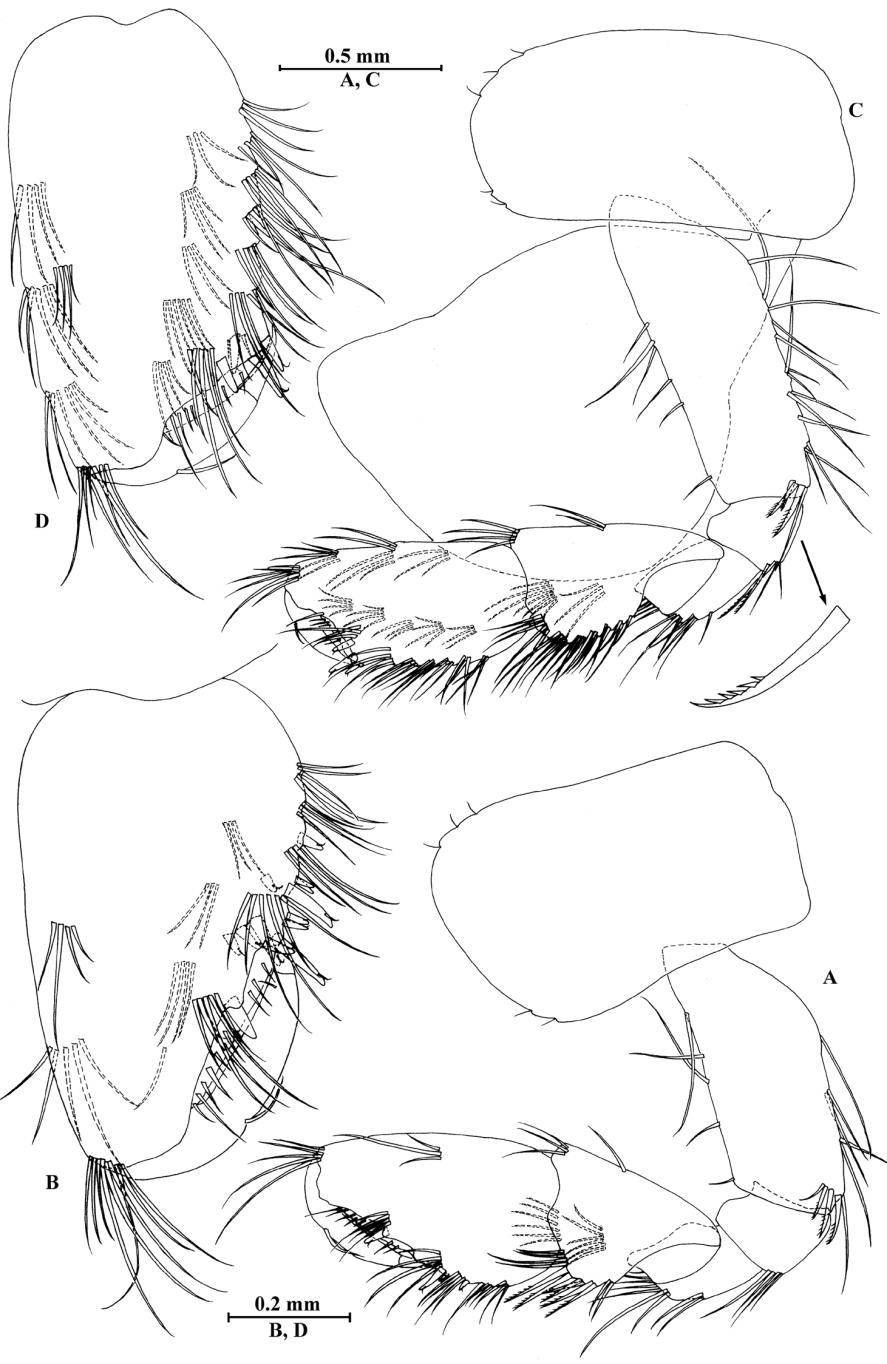
*Gammarus vallecula* Hou & Li, sp. n., male holotype. **A** gnathopod I **B** propodus and dactylus of gnathopod I **C** gnathopod II **D** propodus and dactylus of gnathopod II.

**Figure 4.**
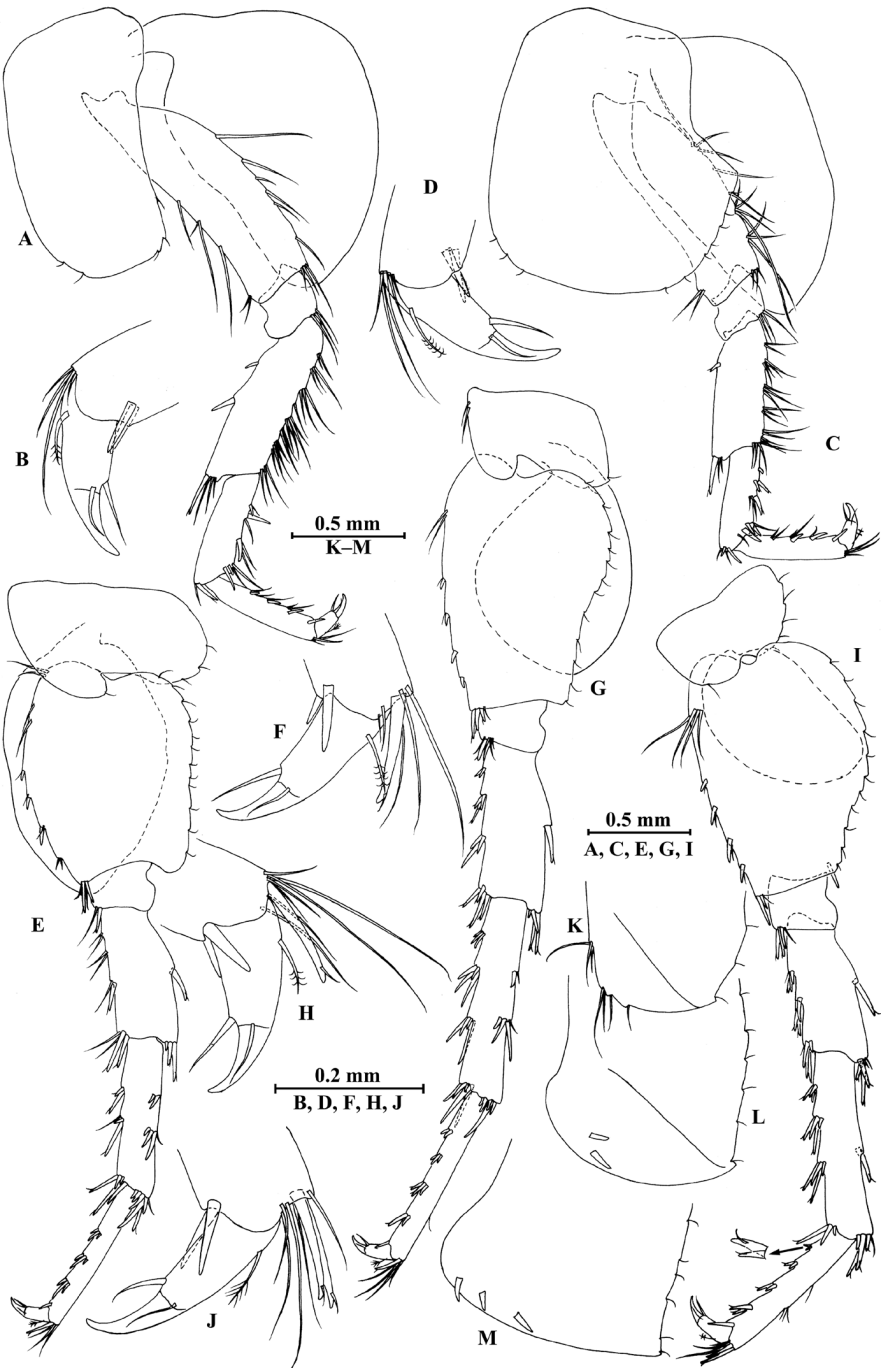
*Gammarus vallecula* Hou & Li, sp. n., male holotype. **A** pereopod III **B** dactylus of pereopod III **C** pereopod IV **D** dactylus of pereopod IV **E** pereopod V **F** dactylus of pereopod V **G** pereopod VI **H** dactylus of pereopod VI **I** pereopod VII **J** dactylus of pereopod VII **K** epimeral plate I **L** epimeral plate II **M** epimeral plate III.

**Figure 5.**
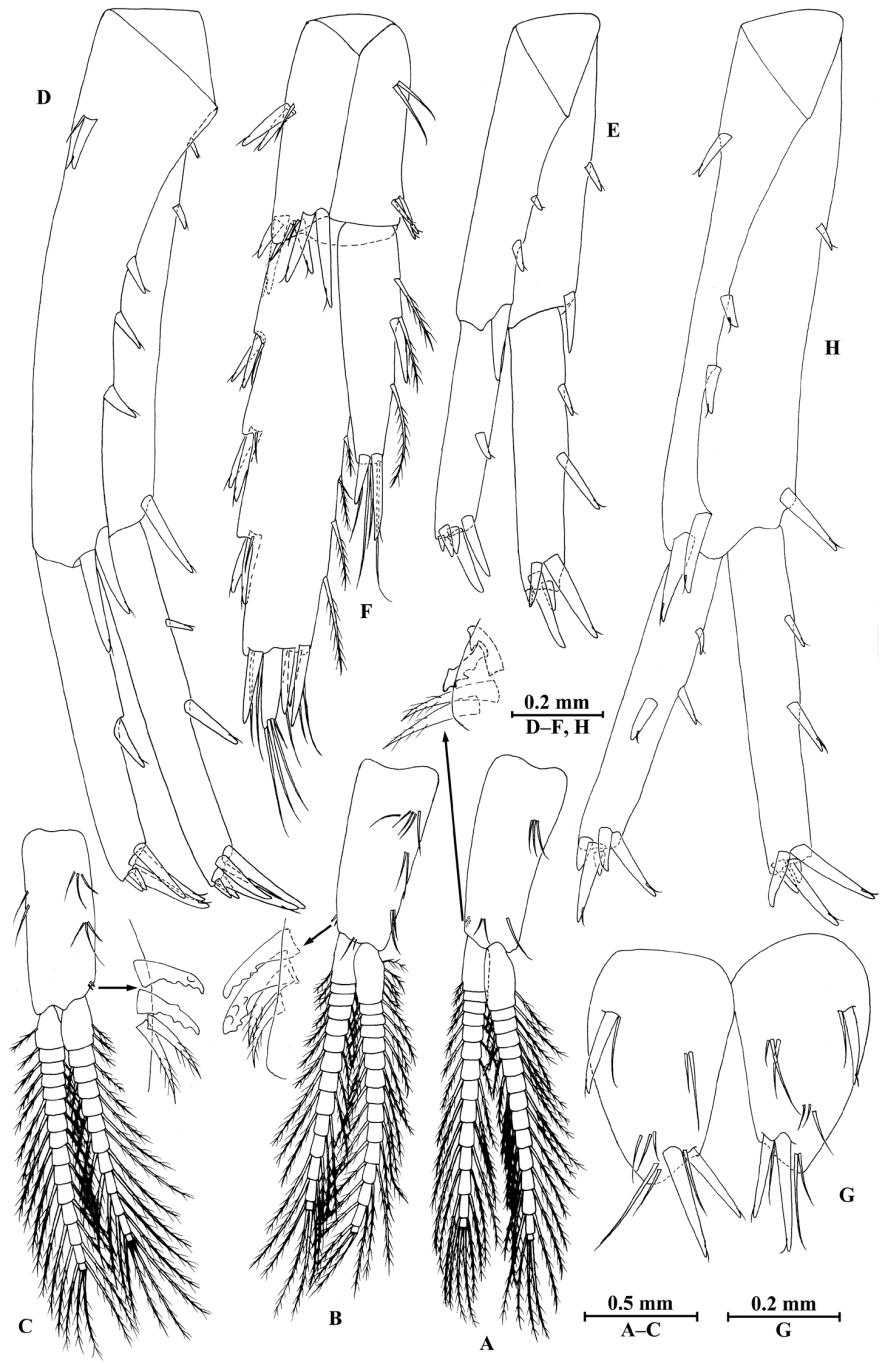
*Gammarus vallecula* Hou & Li, sp. n., **A–G** male, holotype; **H** female, paratype. **A** pleopod I **B** pleopod II **C** pleopod III **D** uropod I **E** uropod II **F** uropod III **G** telson **H** uropod I.

**Figure 6.**
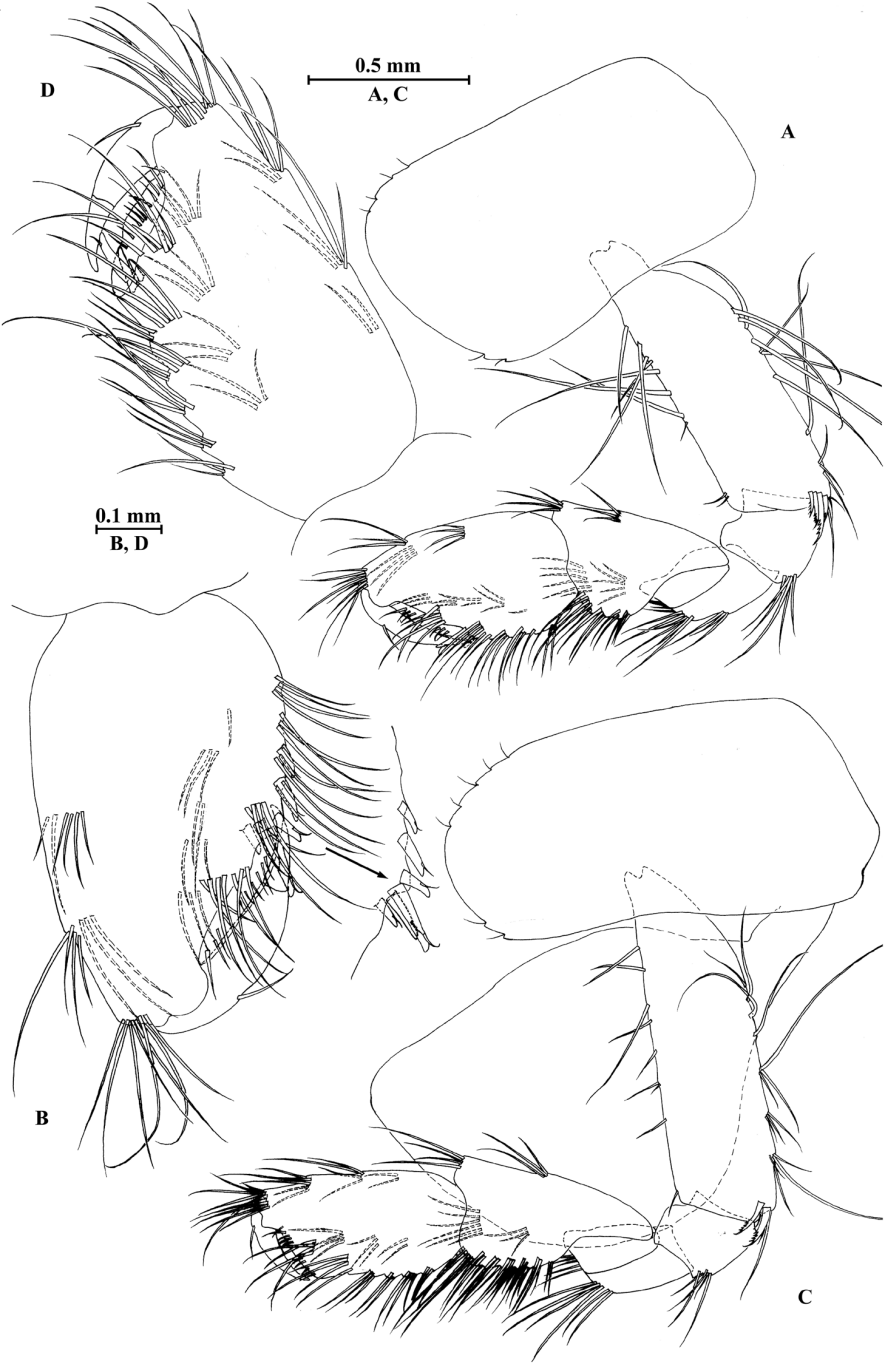
*Gammarus vallecula* Hou & Li, sp. n., female paratype. **A** gnathopod I **B** propodus and dactylus of gnathopod I **C** gnathopod II **D** propodus and dactylus of gnathopod II.

**Figure 7.**
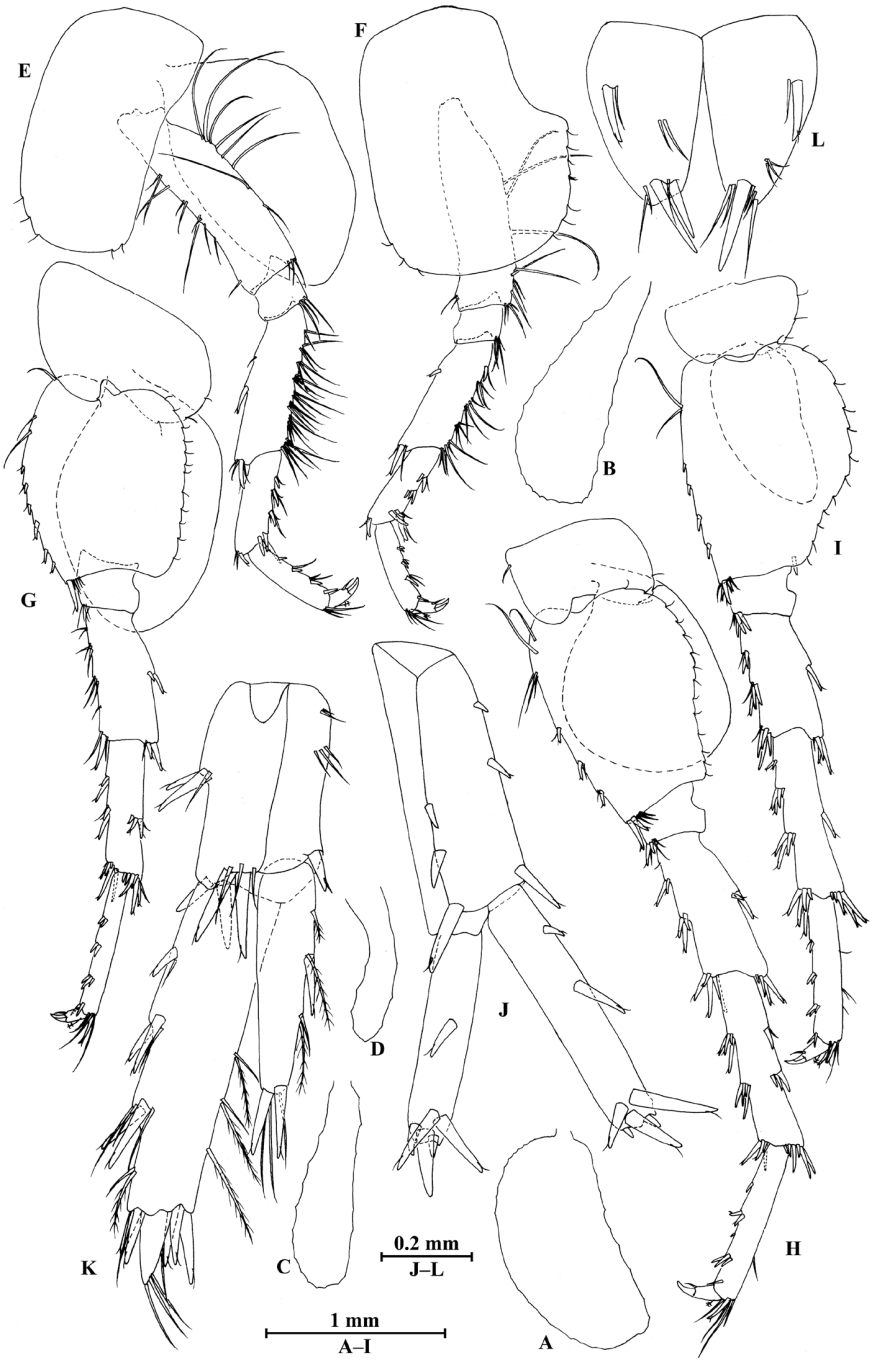
*Gammarus vallecula* Hou & Li, sp. n., female paratype. **A** oostegite of gnathopod II **B** oostegite of pereopod III **C** oostegite of pereopod IV **D** oostegite of pereopod V **E** pereopod III **F** pereopod IV **G** pereopod V **H** pereopod VI **I** pereopod VII **J** uropod II **K** uropod III **L** telson.

Figs 2 , 3 , 4 , 5 , 6 , 7

##### Material examined.

Holotype: male (IZCAS-I-A1411-1), 8.5 mm, Liuba County (106.92°E, 33.61°N), altitude 1001 m, Hanzhong City, Shaanxi Province, China, October 23, 2013, collected by Yunchun Li and Jincheng Liu. Paratype: female (IZCAS-I-A1411-2), 7.8 mm, same data as holotype.

##### Etymology.

The specific name alludes to its typical biotope, living in a valley; adjective.

##### Diagnosis.

Antenna II with setae along peduncle articles and flagellum, calceoli absent; merus to propodus of pereopods III and IV with short straight setae on posterior margins; epimeral plate II with blunt posterodistal corner; epimeral plate III with subacute posterodistal corner; uropod III inner ramus reaching 0.5 times the length of outer ramus, second article of outer ramus subequal to adjacent spines, both rami with a few plumose setae on inner margins.

##### Description of holotype male

(IZCAS-I-A1411-1). 8.5 mm.

***Head*** (Fig. 2A): eyes reniform, inferior antennal sinus deep, lateral cephalic lobe rounded.

*Antenna I* (Fig. 2B, C): peduncle articles I–III in length ratio 1.0: 0.7: 0.4, with distal setae; flagellum with 26 articles, articles IV–XX with aesthetascs; accessory ﬂagellum with four articles; both primary and accessory ﬂagella with short distal setae.

*Antenna II* (Fig. 2D): peduncle articles III–V in length ratio 1.0: 2.5: 2.3, peduncle article III with setae on lateral margin, articles IV and V of peduncle with clusters of lateral and medial setae; flagellum with ten articles, each article with long setae; calceoli absent.

*Upper lip* (Fig. 2E): ventral margin rounded, bearing short minute setae.

*Mandible* (Fig. 2G, H): left mandible incisor with five teeth; lacinia mobilis with four teeth; spine row with five pairs of plumose setae; articles I–III of palp in length ratio 1.0: 3.1: 1.9, second article of palp with 12 marginal setae, article III with four A-setae, four B-setae, 16 D-setae, and five E-setae apically; incisor of right mandible with four teeth, lacinia mobilis bifurcate, with small teeth.

*Lower lip* (Fig. 2F): inner lobes lacking, outer lobes covered with thin setae.

*Maxilla I* (Fig. 2I, J): asymmetrical, left inner plate with nine plumose setae and two simple setae on medial margin; outer plate with 11 robust serrated apical spines, each spine with small teeth; second article of left palp with seven slender spines apically; second article of right palp with five stout spines, one slender spine and one stiff spine.

*Maxilla II* (Fig. 2K): inner plate with three simple setae and 11 plumose facial setae in an oblique row; inner and outer plates with long setae apically.

*Maxilliped* (Fig. 2L): inner plate with three stout apical spines and one subapical spine, 21 plumose setae along lateral margin; outer plate bearing a row of 14 blade spines and three plumose setae apically; article IV of palp hooked, with a group of setae at hinge of unguis.

***Pereon.*** *Gnathopod I* (Fig. 3A, B): coxal plate bearing three setae and two setae on anterior and posterior margins, respectively; basis with setae on anterior and posterior margins, and with three serrated spines accompanied by two setae on posterodistal corner; carpus 1.3 times as long as wide, 0.7 times as long as propodus, ventral margin bearing a cluster of simple setae and three clusters of serrated setae; propodus oval, palm with one medial spine and 12 spines on posterior margin and surface; dactylus with one seta on outer margin.

*Gnathopod II* (Fig. 3C, D): coxal plate bearing three setae and two setae on anterior and posterior margins, respectively; basis with setae on anterior and posterior margins, and with three serrated spines accompanied by one seta on posterodistal corner; carpus 1.8 times as long as wide, 0.9 times as long as propodus, bearing seven clusters of setae along ventral margin, two clusters of setae on dorsal margin; propodus subrectangular, palm margin with one medial spine and three spines on lateral posterodistal corner; dactylus with one seta on outer margin.

*Pereopod III* (Fig. 4A, B): coxal plate bearing two setae on anterior margin and three setae on posterior margin; basis elongated, with setae along anterior and posterior margins; merus with long and straight setae on posterior margin and two spines on anterior margin, anterodistal corner with one spine accompanied by setae; carpus with five spines accompanied by setae on posterior margin, anterodistal corner with one spine and one seta; propodus with three spines accompanied by setae on posterior margin and two spines on posterodistal corner; dactylus with one plumose seta on anterior margin, and two setae at hinge of unguis.

*Pereopod IV* (Fig. 4C, D): coxal plate concave, bearing three setae on anterior margin and seven setae on posterior margin; basis with two setae on anterior margin, with clusters of setae on posterior margin; merus with clusters of long setae on posterior margin and one spine on anterior margin, anterodistal corner with one spine accompanied by setae; carpus with seven spines accompanied by setae on posterior margin, anterodistal corner with two spines accompanied by setae; propodus with two spines accompanied by setae on posterior margin and two spines on posterodistal corner; dactylus with one plumose seta on anterior margin, and two setae at hinge of unguis.

*Pereopod V* (Fig. 4E, F): coxal plate bearing one seta on anterior margin and three setae on posterior margin; basis expanded, with four long setae and three spines accompanied by fine setae on anterior margin, anterodistal corner with two spines accompanied by two setae, posterior margin with a row of 12 setae; merus with one spine accompanied by setae on anterior margin and one spine on posterior margin, anterodistal corner with two spines accompanied by setae and posterodistal corner with three spines; carpus with two groups of spines on anterior and posterior margins each; propodus with three groups of spines on anterior margin; dactylus with one plumose seta on posterior margin, and two setae at hinge of unguis.

*Pereopod VI* (Fig. 4G, H): coxal plate bearing two long setae and two fine setae on anterior and posterior margins, respectively; basis elongated, with two long setae and four spines accompanied by setae on anterior margin, anterodistal corner with three spines and one fine seta, posterior margin with a row of 12 fine setae; merus with three groups of spines and one spine on anterior and posterior margins, respectively, anterodistal and posterodistal corners with four spines each; carpus with three or two groups of spines on anterior and posterior margins, respectively; propodus with four groups of spines on anterior margin; dactylus with one plumose seta on posterior margin, and two setae at hinge of unguis.

*Pereopod VII* (Fig. 4I, J): coxal plate bearing one seta on anterior margin and five setae on posterior margin; basis with four long setae and four spines accompanied by setae on anterior margin, anterodistal corner with two spines and a fine seta, posterior margin with a row of 11 setae, posterodistal corner with one spine; merus with two groups of spines on anterior margin and one spine on posterior margin, anterodistal and posterodistal corners with four and three spines, respectively; carpus with three groups of spines on anterior margin and one spine on posterior margin, anterodistal and posterodistal corners with three and four spines, respectively; propodus with groups of spines on anterior margin; dactylus with one plumose seta on posterior margin, and two setae at hinge of unguis.

*Coxal gills*: coxal gill of gnathopod II and gills of pereopods III–V longer than bases; gill of pereopod VI a little shorter than basis; gill of pereopod VII smallest, half the length of basis.

***Pleon.*** *Epimeral plates* (Fig. 4K–M): plate I ventrally rounded, bearing seven long setae on anteroventral margin and three tiny setae on posterior margin; plate II with two spines on ventral margin and six tiny setae on posterior margin, posterodistal corner blunt; plate III with three spines on ventral margin and six tiny setae on posterior margin, posterodistal corner subacute.

*Pleopods I–III* (Fig. 5A–C): similar, peduncle with two retinacula accompanied by two setae; outer ramus slightly longer than inner ramus, both inner and outer rami fringed with plumose setae.

***Urosome.*** *Urosomites* (Fig. 2M): urosomite I with one-one-one-one spines accompanied by setae on dorsal margin; urosomite II with three-one-one-three spines accompanied by setae on dorsal margin; urosomite III with two spines accompanied by two setae on each side and two pairs of setae on dorsal margin.

*Uropods I–III* (Fig. 5D–F): uropod I peduncle with one basofacial spine, two spines on inner margin and three spines on outer margin, inner and outer distal corners with one and two spines, respectively; inner and outer rami with two and one spines on inner margins, respectively; both rami with five terminal spines. Uropod II peduncle with one and two spines on inner and outer margins respectively, and with one distal spine on each corner; inner ramus with two spines on inner margin; outer ramus with one spine on inner margin; both rami with five terminal spines. Uropod III peduncle with two spines accompanied by one seta on surface and eight distal spines; inner ramus 1.2 times as long as peduncle, reaching 0.5 times the length of outer ramus, with one spine accompanied by three plumose setae on inner margin and two distal spines accompanied by setae; proximal article of outer ramus with three pairs of spines accompanied by simple setae on outer margin, with four plumose setae and one simple seta on inner margin, terminal article with simple setae, subequal to adjacent spines.

*Telson* (Fig. 5G): deeply cleft, 0.8 times as long as wide; each lobe with one spine accompanied by one seta and clusters of setae on surface, bearing two distal spines accompanied by three setae.

##### Description of paratype female

(IZCAS-I-A1411-2). 7.8 mm.

***Pereon.*** *Gnathopod I* (Fig. 6A, B): coxal plate bearing four and two setae on anterior and posterior margins, respectively; basis with long setae on anterior and posterior margins, posterodistal corner with four serrated spines accompanied by setae; propodus oval, palm with six spines on posterior margin, bearing long setae along anterior and posterior margins; dactylus with one seta on outer margin.

*Gnathopod II* (Fig. 6C, D): coxal plate bearing five and two fine setae on anterior and posterior margins, respectively; basis with setae on anterior and posterior margins; propodus subrectangular, palm margin with three spines on posterodistal corner, bearing long setae along anterior and posterior margins; dactylus with one seta on outer margin.

*Pereopods III–VII* (Fig. 7E–I): similar to those of male.

*Oostegite* (Fig. 6H–K): oostegite of gnathopod II broad, oostegites of pereopods III and IV elongated, oostegite of pereopod V smallest.

***Urosome.*** *Uropods I–III* (Figs 5H; 7J, K): uropod I peduncle with one basofacial spine, with two and one spines on outer and inner margins, respectively, with two and one spines on outer and inner distal corners, respectively; outer ramus with two spines on inner margin and one spine on outer margin; inner ramus with two spines on inner margin; both rami with five terminal spines. Uropod II short, peduncle bearing two spines on outer and inner margins each, with one spine on outer and inner distal corners each; outer ramus with one spine on outer margin; inner ramus with two spines on inner margin; both rami with five terminal spines. Uropod III peduncle with two spines accompanied by one seta on surface and five distal spines; inner ramus 1.1 times as long as peduncle, reaching 0.6 times the length of outer ramus, with one spine accompanied by three plumose setae and one simple seta on inner margin and two distal spines accompanied by setae; proximal article of outer ramus with five spines accompanied by one plumose seta and simple setae on outer margin, inner margin with three plumose setae and two simple setae, terminal article subequal to adjacent spines.

*Telson* (Fig. 7L): cleft, 0.8 times as long as wide; left lobe with one spine accompanied by one seta and a cluster of two setae on surface, bearing two distal spines accompanied by three setae; right lobe with one spine accompanied by one seta and a cluster of three setae on surface, bearing one distal spine accompanied by five setae.

##### Habitat.

This species was collected from a valley of south part of the Qinling. Individuals inhabit a stream, usually under decomposing leaves.

##### Remarks.

The new species of *Gammarus vallecula* Hou & Li, sp. n. is similar to *G. craspedotrichus* Hou & Li, 2002a in antenna II calceoli absent; pereopods III and IV with straight setae on posterior margins; and both rami of uropod III with plumose setae on inner margins. It differs from *G. craspedotrichus* (*G. craspedotrichus* in parentheses) by peduncle of antenna II with setae along ventral margin, setae as long as article’s diameter (antenna II with long setae along ventral margin, setae as long as three times of article’s diameter); uropod I peduncle with one basofacial spine (without basofacial spine); inner ramus of uropod III 0.5 times the length of outer ramus (as long as first article of outer ramus); terminal article of outer ramus of uropod III subequal to adjacent spines (shorter); and urosomites I–III with four clusters of dorsal spines and setae (with two clusters of dorsal spines and setae).

*Gammarus vallecula* Hou & Li, sp. n. is also similar to *G. emeiensis* Hou, Li & Koenemann, 2002 in antenna II calceoli absent; epimeral plate II with blunt posterodistal corner and plate III with subacute posterodistal corner; peduncle of uropod I with one basofacial spine; and terminal article of outer ramus of uropod III approx. as long as adjacent spines of first article. It can be distinguished from *G. emeiensis* by the following characters (*G. emeiensis* in parentheses): second article of left palp of maxilla I with seven slender spines apically (seven slender spines and three stiff setae); pereopod III with short setae on posterior margin (with long setae on posterior margin); and inner ramus of uropod III 0.5 times the length of outer ramus (0.74 times the length of first article of outer ramus).

The new species of *Gammarus vallecula* Hou & Li, sp. n. can be distinguished from *G. martensi* Hou & Li, 2004a which was collected on the summit of the Qinling by the following characters (*G. martensi* in parentheses): antenna II flagellum with a few setae, calceoli absent (with flag-like brush of setae, calceoli present); merus and carpus of pereopods V–VII with few marginal setae (with marginal setae); and uropod III inner ramus approx. half of outer ramus, both with a few plumose setae on inner margins (inner ramus 0.75 times as long as outer ramus, both rami densely with plumose setae on inner and outer margins).

#### Gammarus qinling

Taxon classificationAnimaliaAmphipodaGammaridae

Hou & Li sp. n.

http://zoobank.org/DD98C03F-55E1-4A97-9D00-686ECAC39F54

**Figure 8.**
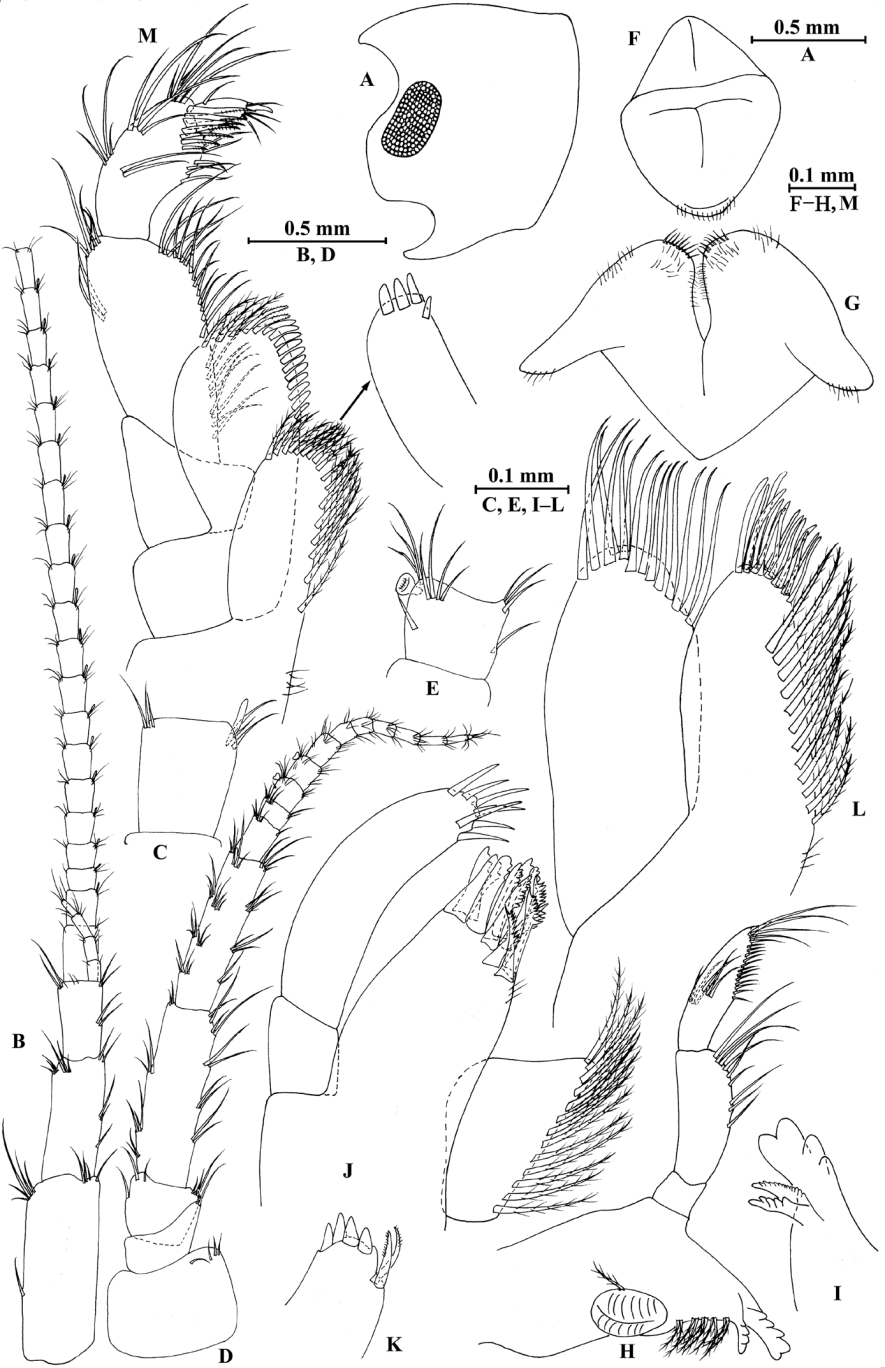
*Gammarus qinling* Hou & Li, sp. n., male holotype. **A** head **B** antenna I **C** flagellar article of antenna I with aesthetasc **D** antenna II **E** calceoli of antenna II **F** upper lip **G** lower lip **H** left mandible **I** incisor and lacinia mobilis of right mandible **J** left maxilla I **K** distal part of palp article II of right maxilla I **L** maxilla II **M** maxilliped.

**Figure 9.**
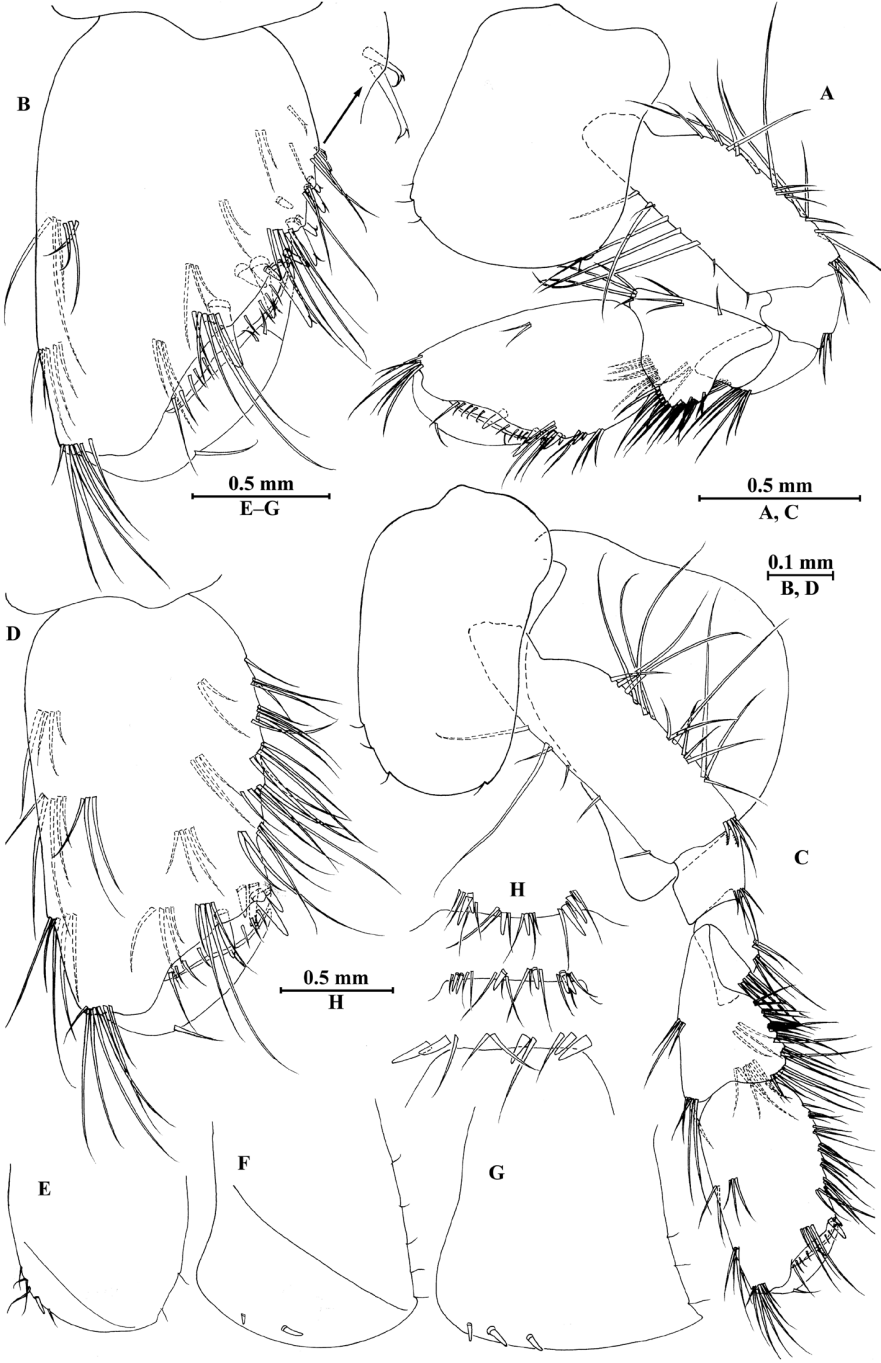
*Gammarus qinling* Hou & Li, sp. n., male holotype. **A** gnathopod I **B** propodus and dactylus of gnathopod I **C** gnathopod II **D** propodus and dactylus of gnathopod II **E** epimeral plate I **F** epimeral plate II **G** epimeral plate III **H** dorsal margins of urosomites I–III.

**Figure 10.**
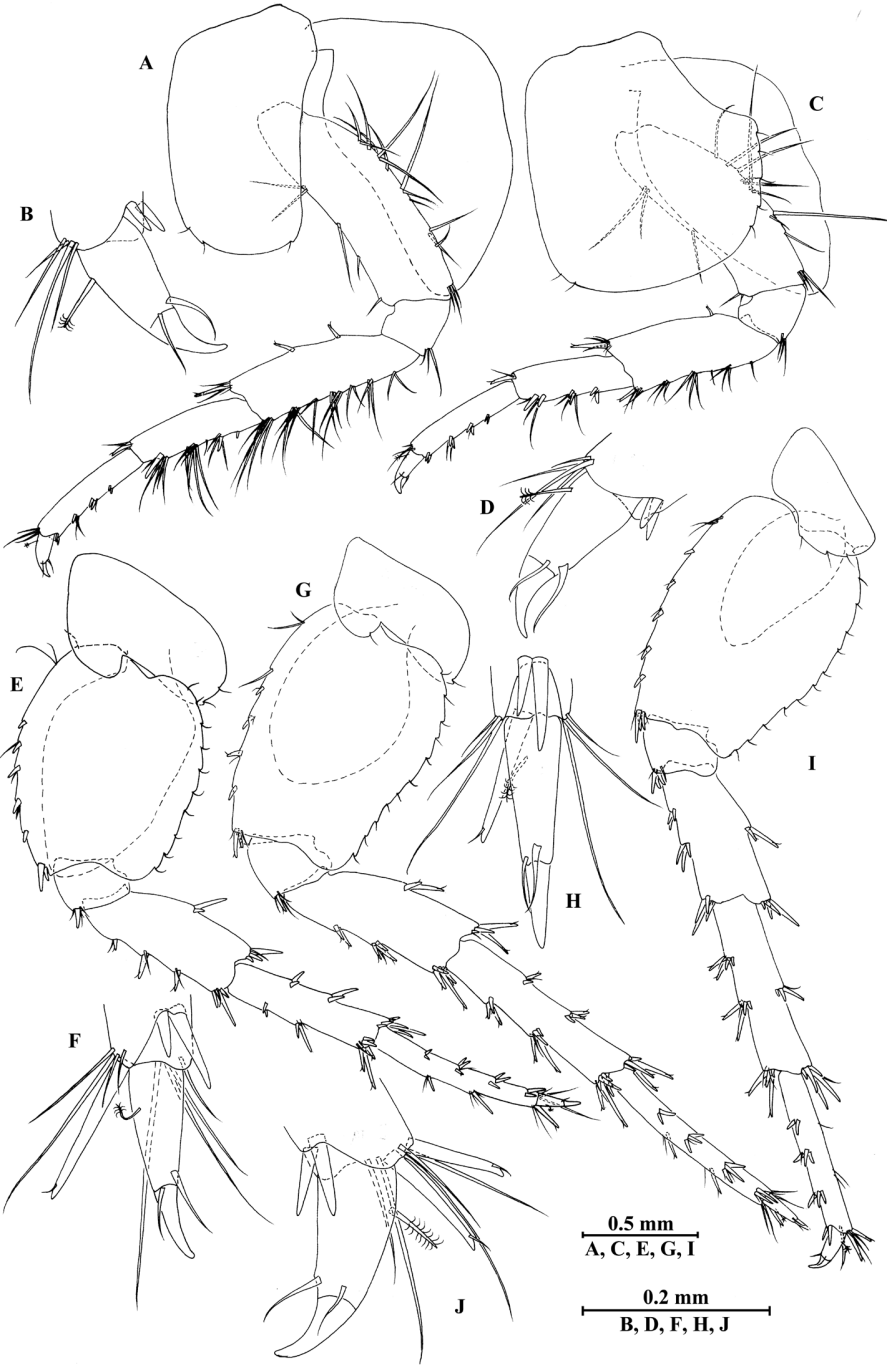
*Gammarus qinling* Hou & Li, sp. n., male holotype. **A** pereopod III **B** dactylus of pereopod III **C** pereopod IV **D** dactylus of pereopod IV **E** pereopod V **F** dactylus of pereopod V **G** pereopod VI **H** dactylus of pereopod VI **I** pereopod VII **J** dactylus of pereopod VII.

**Figure 11.**
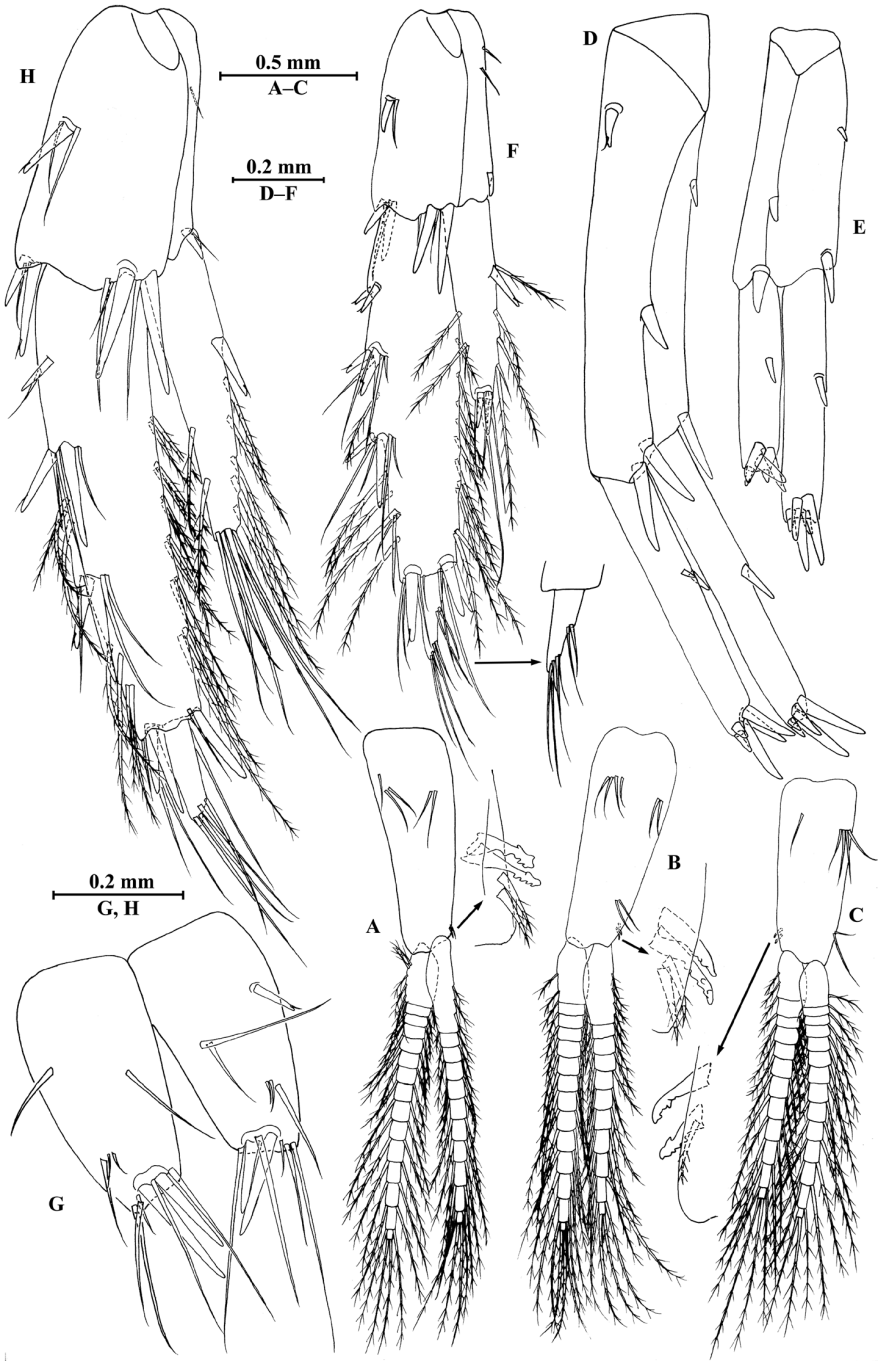
*Gammarus qinling* Hou & Li, sp. n., **A–G** male, holotype; **H** female, paratype. **A** pleopod I **B** pleopod II **C** pleopod III **D** uropod I **E** uropod II **F** uropod III **G** telson **H** uropod III.

**Figure 12.**
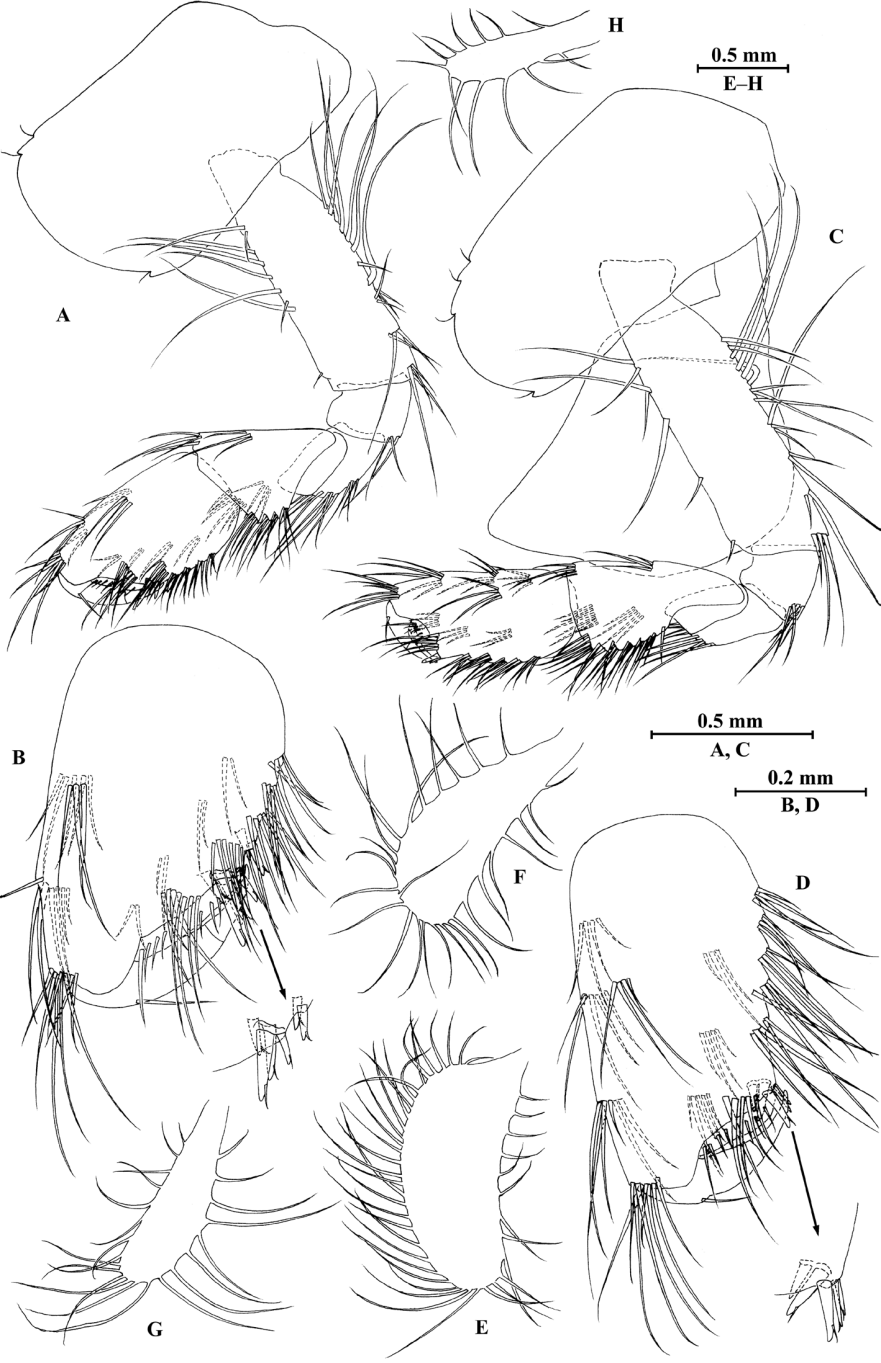
*Gammarus qinling* Hou & Li, sp. n., female paratype. **A** gnathopod I **B** propodus and dactylus of gnathopod I **C** gnathopod II **D** propodus and dactylus of gnathopod II **E** oostegite of gnathopod II **F** oostegite of pereopod III **G** oostegite of pereopod IV **H** oostegite of pereopod V.

**Figure 13.**
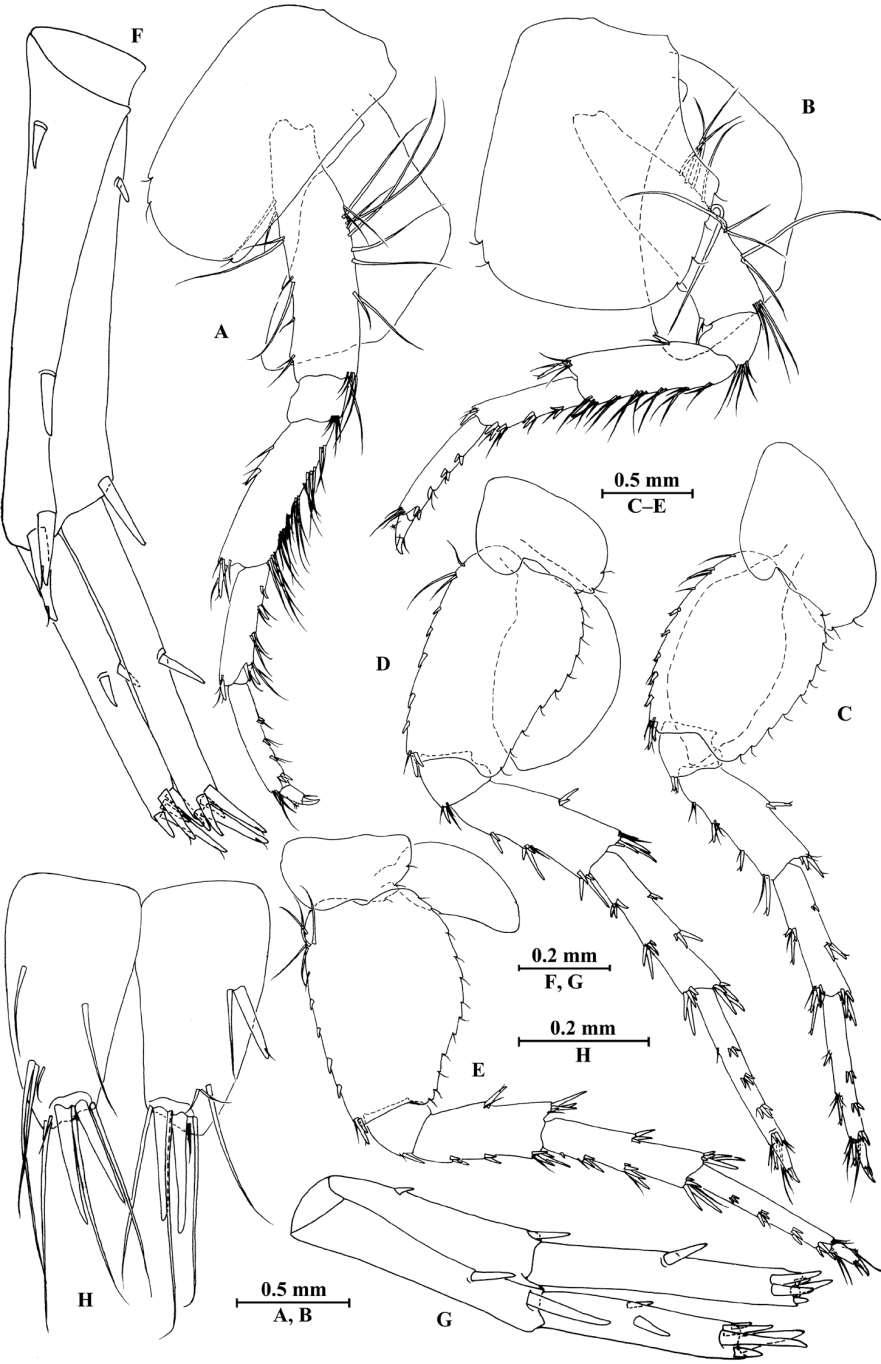
*Gammarus qinling* Hou & Li, sp. n., female paratype. **A** pereopod III **B** pereopod IV **C** pereopod V **D** pereopod VI **E** pereopod VII **F** uropod I **G** uropod II **H** telson.

Figs 8 , 9 , 10 , 11 , 12 , 13

##### Material examined.

Holotype: male (IZCAS-I-A1416-1), 8.3 mm, Zibo Mountain National Forest Park (106.82°E, 33.67°N), altitude 1352 m, Liuba County, Hanzhong City, Shaanxi Province, China, October 24, 2013, collected by Yunchun Li and Jincheng Liu. Paratype: female (IZCAS-I-A1416-2), 9.4 mm, same data as holotype.

##### Etymology.

The specific name is derived from the type locality; noun in apposition.

##### Diagnosis.

Antenna II calceoli present in male; pereopods III and IV with short straight setae on posterior margins of merus and propodus; epimeral plates II and III with blunt posterodistal corners; uropod III inner ramus reaching half the length of outer ramus, terminal article of outer ramus a little longer than adjacent spines, both rami with plumose setae on inner and outer margins.

##### Description of holotype male

(IZCAS-I-A1416-1). 8.3 mm.

***Head*** (Fig. 8A): eyes oval, inferior antennal sinus deep, lateral cephalic lobe rounded.

*Antenna I* (Fig. 8B, C): peduncle articles I–III in length ratio 1.0: 0.6: 0.4, with lateral and distal setae; flagellum incomplete, articles II–XIX with aesthetascs; accessory ﬂagellum with four articles; both primary and accessory ﬂagella with short distal setae.

*Antenna II* (Fig. 8D, E): peduncle articles III–V in length ratio 1.0: 2.7: 2.4, article III with distal setae, articles IV and V with clusters of lateral and medial setae; flagellum with 11 articles and one tiny distal article, with setae along dorsal and ventral margins; articles III and IV with calceoli.

*Upper lip* (Fig. 8F): ventral margin rounded, bearing short minute setae.

*Mandible* (Fig. 8H, I): left mandible incisor with five teeth; lacinia mobilis with four teeth; spine row with five pairs of plumose setae; articles I–III of palp in length ratio 1.0: 3.7: 3.8, second article of palp with nine marginal setae, article III with three A-setae, three B-setae, 12 D-setae and five E-setae apically; incisor of right mandible with four teeth, lacinia mobilis bifurcate, with small teeth.

*Lower lip* (Fig. 8G): inner lobes lacking, outer lobes covered with thin setae.

*Maxilla I* (Fig. 8J, K): asymmetrical, left inner plate with 13 plumose setae on medial margin; outer plate with 11 robust serrated apical spines, each spine with small teeth; second article of left palp with seven slender spines apically; second article of right palp with four stout spines and two slender spines.

*Maxilla II* (Fig. 8L): inner plate with three fine setae and 12 plumose facial setae in an oblique row; inner and outer plates with long setae apically.

*Maxilliped* (Fig. 8M): inner plate with three stout apical spines and one subapical spine, 17 plumose setae along lateral margin; outer plate bearing a row of 13 blade spines and three plumose setae apically; article IV of palp hooked, with a group of setae at hinge of unguis.

***Pereon.*** *Gnathopod I* (Fig. 9A, B): coxal plate bearing three setae and one seta on anterior and posterior margins, respectively; basis with setae on anterior and posterior margins; carpus 1.1 times as long as wide, 0.6 times as long as propodus, ventral margin bearing four clusters of setae; propodus oval, palm with one medial spine and ten spines on posterior margin and surface; dactylus with one seta on outer margin.

*Gnathopod II* (Fig. 9C, D): coxal plate bearing three setae and one seta on anterior and posterior margins, respectively; basis with setae on anterior and posterior margins, and with two serrated spines accompanied by two setae on posterodistal corner; carpus 1.7 times as long as wide, 0.8 times as long as propodus, bearing six clusters of setae along ventral margin, two clusters of setae on dorsal margin; propodus subrectangular, palm margin with one medial spine and four spines on lateral posterodistal corner; dactylus with one seta on outer margin.

*Pereopod III* (Fig. 10A, B): coxal plate bearing two setae on anterior margin and one seta on posterior margin; basis elongated, with setae along anterior and posterior margins; merus with straight setae on posterior margin and two spines on anterior margin, anterodistal corner with one spine accompanied by setae; carpus with three spines accompanied by long setae on posterior margin; propodus with five spines accompanied by short setae on posterior margin and two spines on posterodistal corner; dactylus with one plumose seta on anterior margin, and two setae at hinge of unguis.

*Pereopod IV* (Fig. 10C, D): coxal plate concave, bearing two setae on anterior margin and five setae on posterior margin; basis with setae along anterior and posterior margins; merus with clusters of short straight setae on posterior margin and one spine on anterior margin, anterodistal and posterodistal corners with one spine accompanied by setae each; carpus with three pairs of spines accompanied by setae on posterior margin, anterodistal corner with one spine accompanied by one seta; propodus with three pairs of spines accompanied by setae on posterior margin and two spines on posterodistal corner; dactylus with one plumose seta on anterior margin, and two setae at hinge of unguis.

*Pereopod V* (Fig. 10E, F): coxal plate bearing two setae on posterior margin; basis sub-oval, with three simple setae and five spines accompanied by fine setae on anterior margin, anterodistal corner with two spines, posterior margin with a row of ten setae; merus with three spines accompanied by setae on anterior margin and two spines on posterior margin, anterodistal and posterodistal corners with three spines accompanied by one seta each; carpus with two pairs of spines on anterior and posterior margins each; propodus with three groups of spines on anterior margin; dactylus with one plumose seta on posterior margin, and two setae at hinge of unguis.

*Pereopod VI* (Fig. 10G, H): coxal plate bearing one seta on anterior and posterior margins each; basis expanded, with three simple setae and four spines accompanied by setae on anterior margin, anterodistal corner with two spines and two fine setae, posterior margin with a row of 11 fine setae; merus with two groups of spines on anterior margin and a pair of spines on posterior margin, anterodistal and posterodistal corners with four spines each; carpus with groups of spines on anterior and posterior margins, anterodistal corner with five spines accompanied by one fine seta and posterodistal corner with five spines; propodus with groups of spines on anterior margin; dactylus with one plumose seta on posterior margin, and two setae at hinge of unguis.

*Pereopod VII* (Fig. 10I, J): coxal plate bearing three setae on posterior margin; basis with two simple setae and five spines accompanied by setae on anterior margin, anterodistal corner with three spines and two fine setae, posterior margin with a row of 12 setae; merus with two groups of spines on anterior margin and a pair of spines on posterior margin, anterodistal and posterodistal corners with four spines each; carpus with two groups of spines on anterior margin and three spines on posterior margin, anterodistal corner with three spines accompanied by two fine setae and posterodistal corner with five spines accompanied by one seta; propodus with three groups of spines on anterior margin; dactylus with one plumose seta on posterior margin, and two setae at hinge of unguis.

*Coxal gills*: coxal gill of gnathopod II and gills of pereopods IV and V a little longer than bases; gill of pereopod III approx. as long as basis; gill of pereopod VI a little shorter than basis; gill of pereopod VII smallest, more than half the length of basis.

***Pleon.*** *Epimeral plates* (Fig. 9E–G): plate I ventrally rounded, bearing five setae and one spine on anteroventral margin and two tiny setae on posterior margin; plate II with two spines on ventral margin and five tiny setae on posterior margin, posterodistal corner blunt; plate III with three spines on ventral margin and three tiny setae on posterior margin, posterodistal corner subacute.

*Pleopods I–III* (Fig. 11A–C): similar, peduncle with two retinacula accompanied by one or two plumose setae; outer ramus slightly shorter than inner ramus, both inner and outer rami fringed with plumose setae.

***Urosome.*** *Urosomites* (Fig. 9H). urosomite I with two-one-one-two spines accompanied by setae on dorsal margin; urosomite II with two-one-one-two spines accompanied by setae on dorsal margin; urosomite III with two spines accompanied by one seta on each side and one spine accompanied by three setae on dorsal margin.

*Uropods I–III* (Fig. 11D–F): uropod I peduncle with one basofacial spine, one spine on inner margin and one spine on outer margin, inner and outer distal corners with one and two spines, respectively; inner ramus with one spine on inner margin; outer ramus with one spine on inner and outer margins each; both rami with five terminal spines. Uropod II peduncle with one spine on inner and outer margins each, and with one distal spine on each corner; both rami with one spine on inner margins and five terminal spines. Uropod III peduncle with one spine accompanied by one seta on surface and five distal spines; inner ramus 0.9 times as long as peduncle, reaching 0.5 times the length of outer ramus, with one spine accompanied by four plumose setae and one simple seta on inner margin, two plumose setae and one simple seta on outer margin, and two distal spines accompanied by setae; proximal article of outer ramus with three pairs of spines accompanied by five plumose setae and simple setae on outer margin, with ten plumose setae on inner margin, terminal article with simple setae, a little longer than adjacent spines.

*Telson* (Fig. 11G): deeply cleft, approx. as long as wide; left lobe with two single setae and a cluster of three setae on surface; right lobe with one spine and two clusters of setae on surface; each lobe bearing two distal spines accompanied by setae.

##### Description of paratype female

(IZCAS-I-A1416-2), 9.4 mm.

***Pereon.*** *Gnathopod I* (Fig. 12A, B): coxal plate bearing two and one setae on anterior and posterior margins, respectively; basis with long setae on anterior and posterior margins; propodus oval, palm with six spines on posterior margin, bearing long setae along anterior and posterior margins; dactylus with one seta on outer margin.

*Gnathopod II* (Fig. 12C, D): coxal plate bearing three and one setae on anterior and posterior margins, respectively; basis with long setae on anterior and posterior margins; propodus subrectangular, palm margin with three stout spines and two stiff spines on posterodistal corner, bearing long setae along anterior and posterior margins; dactylus with one seta on outer margin.

*Pereopods III and IV* (Fig. 13A, B): carpus with more setae on posterior margins than those of male.

*Pereopods V–VII* (Fig. 13C–E): similar to those of male.

*Oostegite* (Fig. 12E–H): oostegite of gnathopod II broad, with marginal setae, oostegites of pereopods III and IV elongate, oostegite of pereopod V smallest.

***Urosome.*** *Uropods I–III* (Figs 11H; 13F, G): Uropods I and II similar to those of male. Uropod III peduncle with one spine accompanied by two setae on surface and five distal spines; inner ramus 1.2 times as long as peduncle, reaching 0.5 times the length of outer ramus, with one spine accompanied by five plumose setae on inner margin and two plumose setae on outer margin; proximal article of outer ramus with three clusters of spines accompanied by plumose setae and simple setae on outer margin, with six pairs of plumose setae on inner margin, terminal article a little longer than adjacent spines.

*Telson* (Fig. 13H): cleft, approx. as long as wide; left lobe with two single setae and a cluster of three setae on surface; right lobe with one spine accompanied by one seta and a cluster of three setae on surface; each lobe bearing two distal spines accompanied by setae.

##### Habitat.

Specimens were collected from a spring of Wulong Cave in Zibo Mountain National Forest Park, which is famous for the specific topography of sinkholes. Zibo Mountain is located in the south of Qinling.

##### Remarks.

This new species of *Gammarus qinling* Hou & Li, sp. n. is most similar to *G. vallecula* Hou & Li, sp. n. in pereopods III and IV with short setae on posterior margins; pereopods V–VII with spines along anterior and posterior margins, but few setae; and epimeral plates II and III posterior margins blunt. *Gammarus qinling* Hou & Li, sp. n. can be distinguished from *G. vallecula* Hou & Li, sp. n. by the following characters (*G. vallecula* in parentheses): antenna II calceoli absent (present); uropod III inner ramus approx. half the length of outer ramus, both rami armed with plumose setae (uropod III approx. half the length of outer ramus, both rami with a few plumose setae on inner margins, outer margins with no plumose setae).

This new species of *Gammarus qinling* Hou & Li, sp. n. can be distinguished from the closely related species *G. murarius* Hou & Li, 2004a (*G. murarius* in parentheses) by the following characters: merus and carpus of pereopod III with straight setae on posterior margins (with long curled setae); epimeral plate I bearing five setae and one spine on anteroventral margin (only with four setae); and inner ramus of uropod III 0.5 times the length of outer ramus (0.65 times the length of first article of outer ramus).

#### Gammarus zhigangi

Taxon classificationAnimaliaAmphipodaGammaridae

Hou & Li sp. n.

http://zoobank.org/8303CC73-FE70-41D1-95E5-D8F23B2DF809

**Figure 14.**
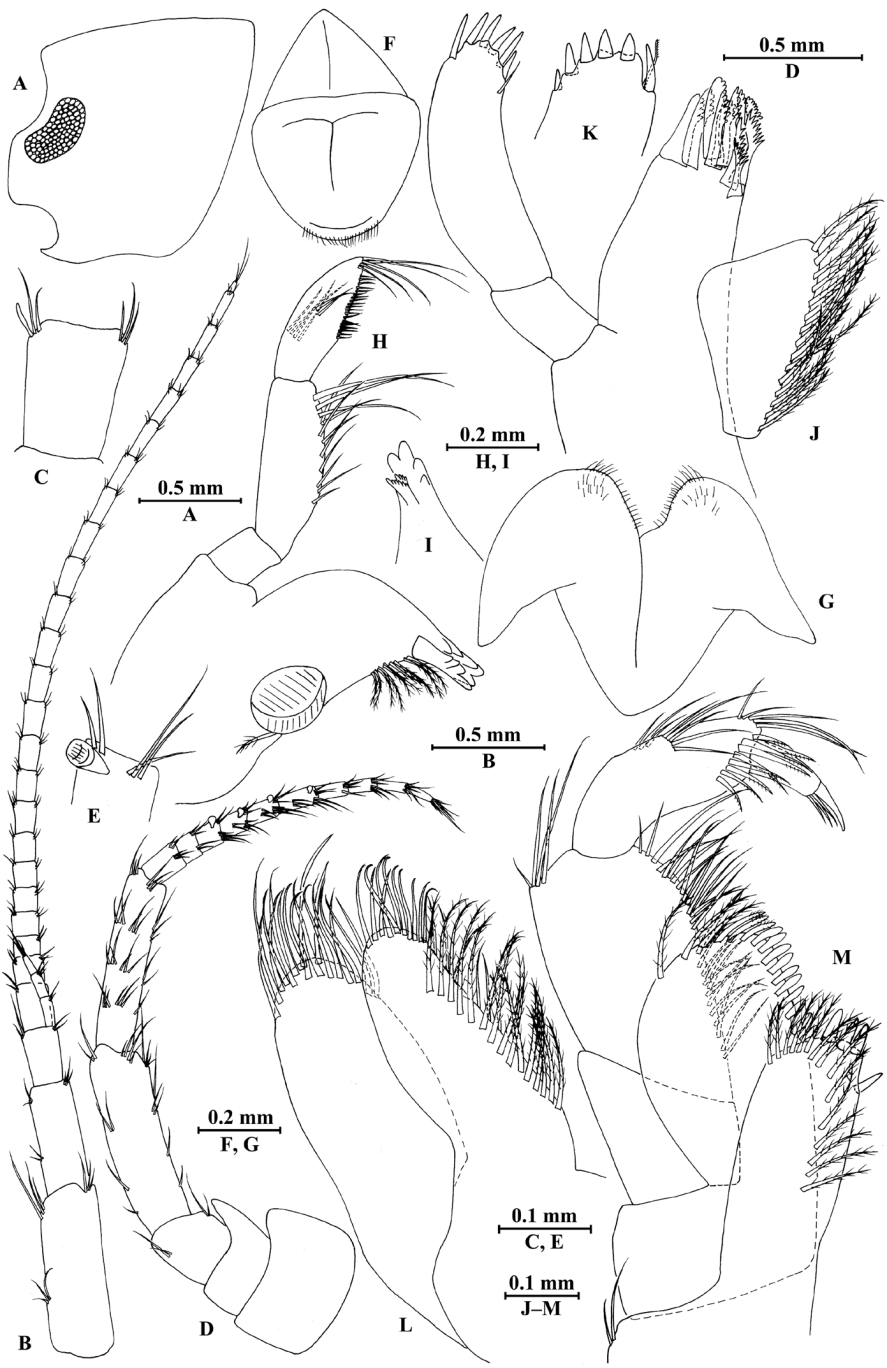
*Gammarus zhigangi* Hou & Li, sp. n., male holotype. **A** head **B** antenna I **C** flagellar article of antenna I with aesthetasc **D** antenna II **E** calceoli of antenna II **F** upper lip **G** lower lip **H** left mandible **I** incisor and lacinia mobilis of right mandible **J** left maxilla I **K** distal part of palp article II of right maxilla I **L** maxilla II **M** maxilliped.

**Figure 15.**
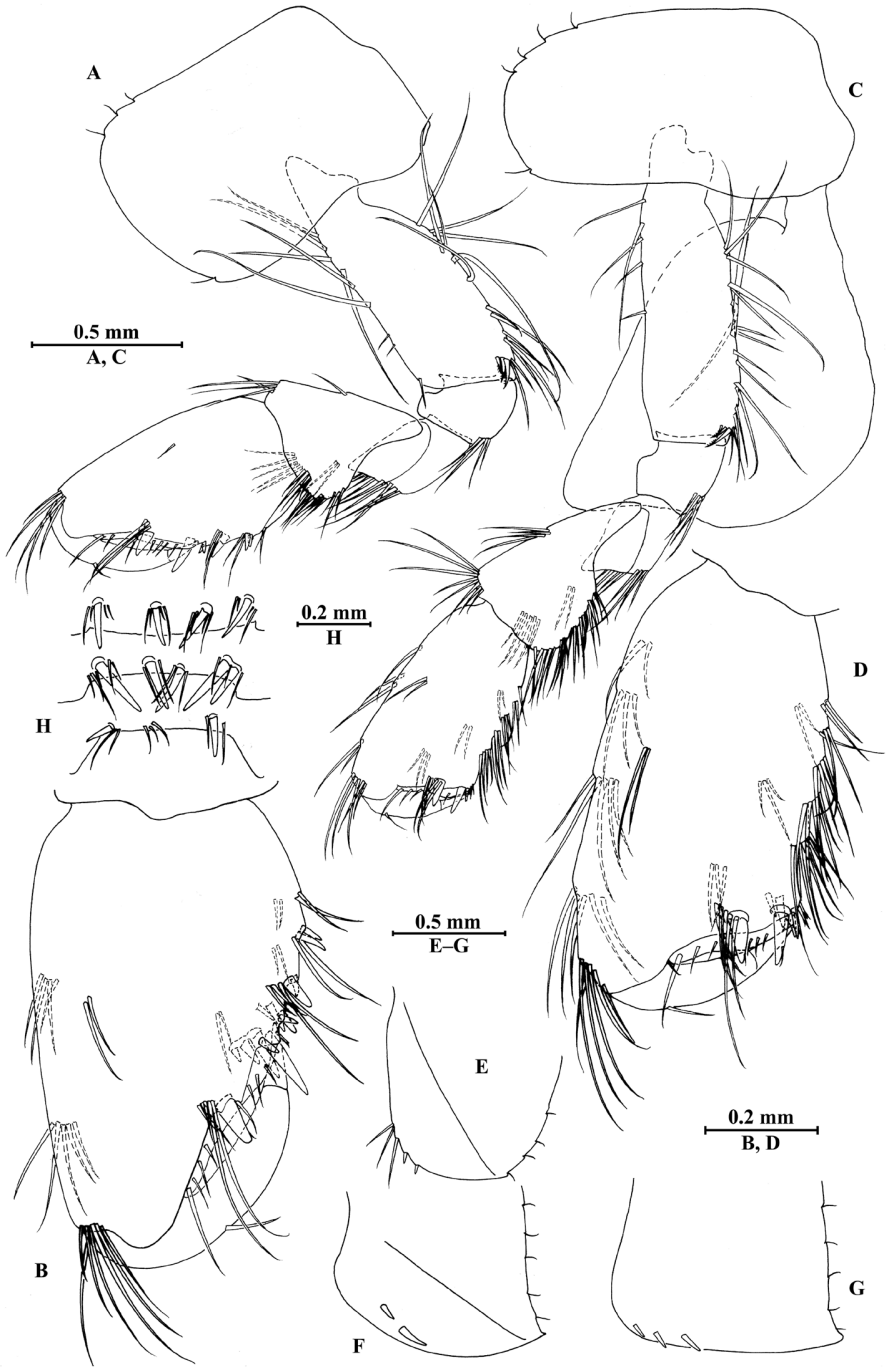
*Gammarus zhigangi* Hou & Li, sp. n., male holotype. **A** gnathopod I **B** propodus and dactylus of gnathopod I **C** gnathopod II **D** propodus and dactylus of gnathopod II **E** epimeral plate I **F** epimeral plate II **G** epimeral plate III **H** dorsal margins of urosomites I–III.

**Figure 16.**
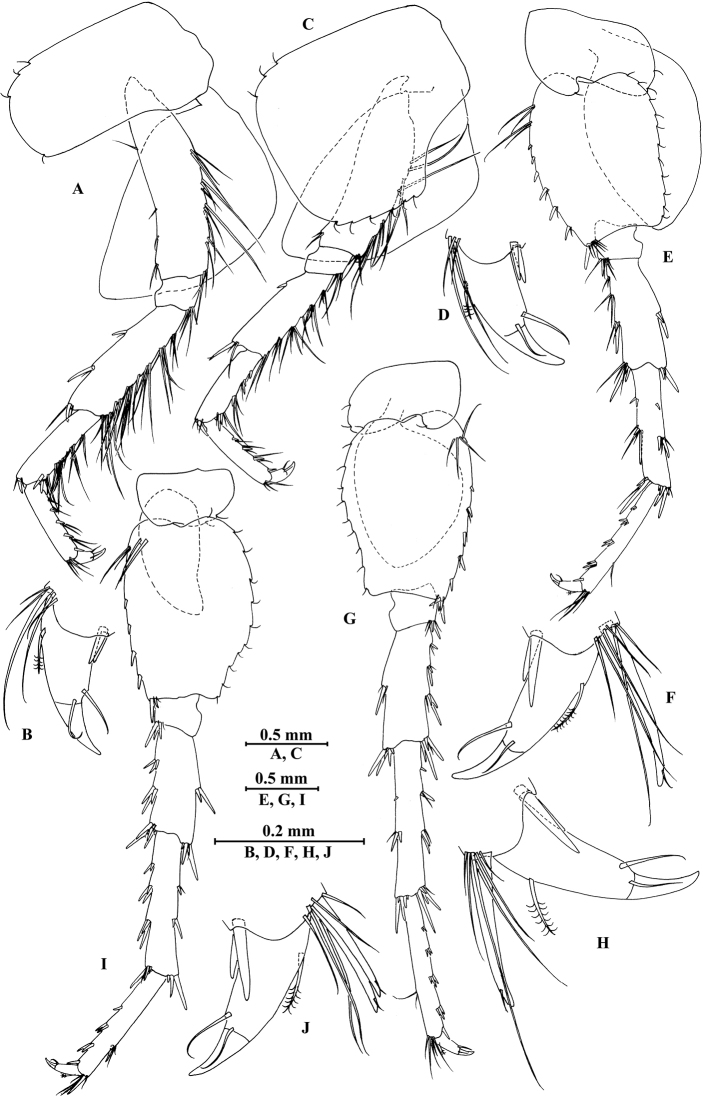
*Gammarus zhigangi* Hou & Li, sp. n., male holotype. **A** pereopod III **B** dactylus of pereopod III **C** pereopod IV **D** dactylus of pereopod IV **E** pereopod V **F** dactylus of pereopod V **G** pereopod VI **H** dactylus of pereopod VI **I** pereopod VII **J** dactylus of pereopod VII.

**Figure 17.**
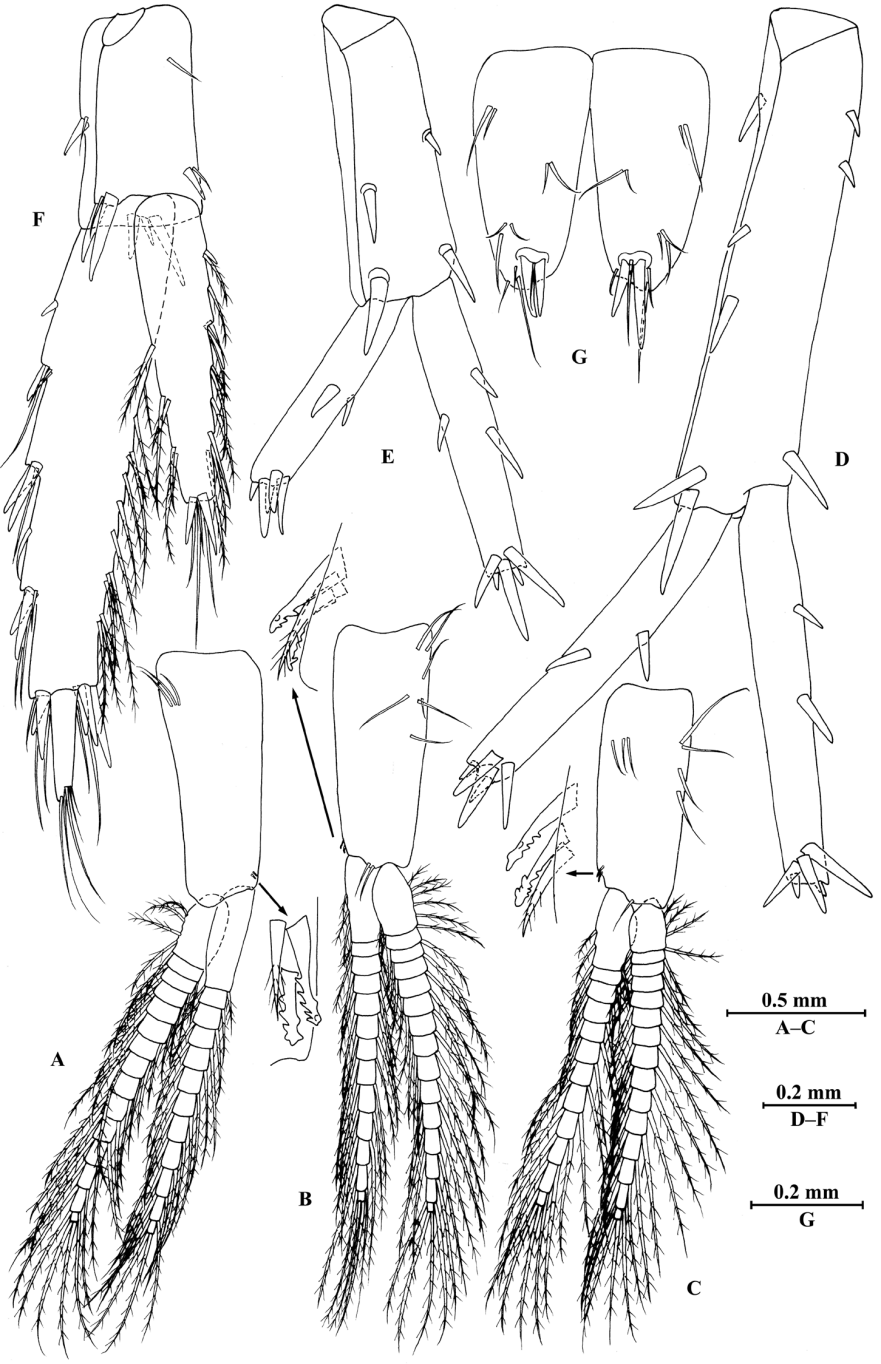
*Gammarus zhigangi* Hou & Li, sp. n., male holotype. **A** pleopod I **B** pleopod II **C** pleopod III **D** uropod I **E** uropod II **F** uropod III **G** telson.

**Figure 18.**
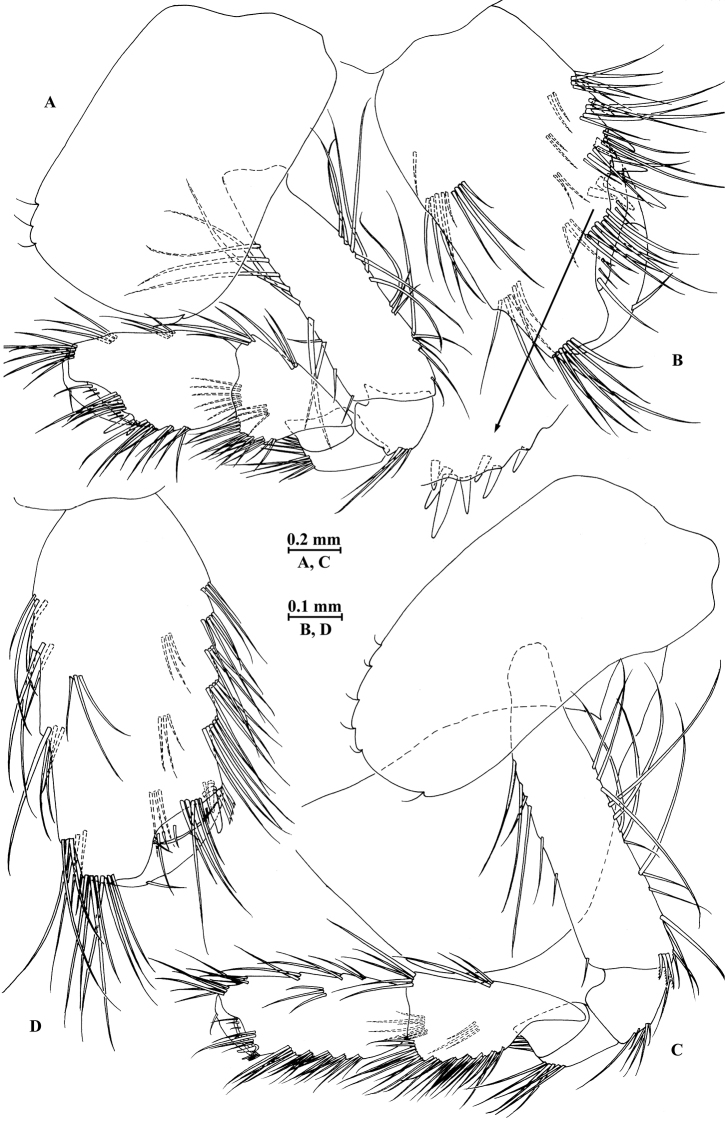
*Gammarus zhigangi* Hou & Li, sp. n., female paratype. **A** gnathopod I **B** propodus and dactylus of gnathopod I **C** gnathopod II **D** propodus and dactylus of gnathopod II.

**Figure 19.**
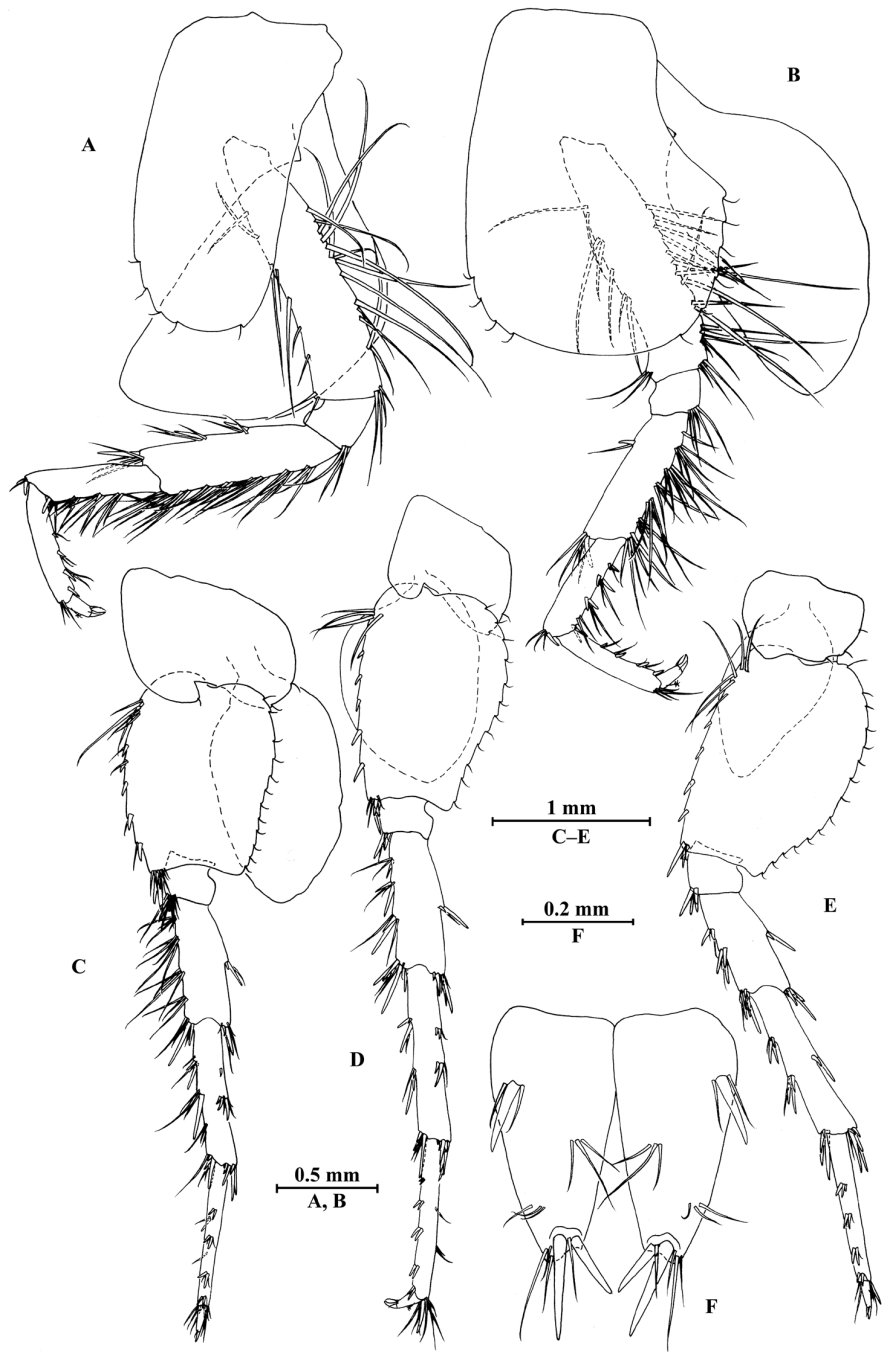
*Gammarus zhigangi* Hou & Li, sp. n., female paratype. **A** pereopod III **B** pereopod IV **C** pereopod V **D** pereopod VI **E** pereopod VII **F** telson.

**Figure 20.**
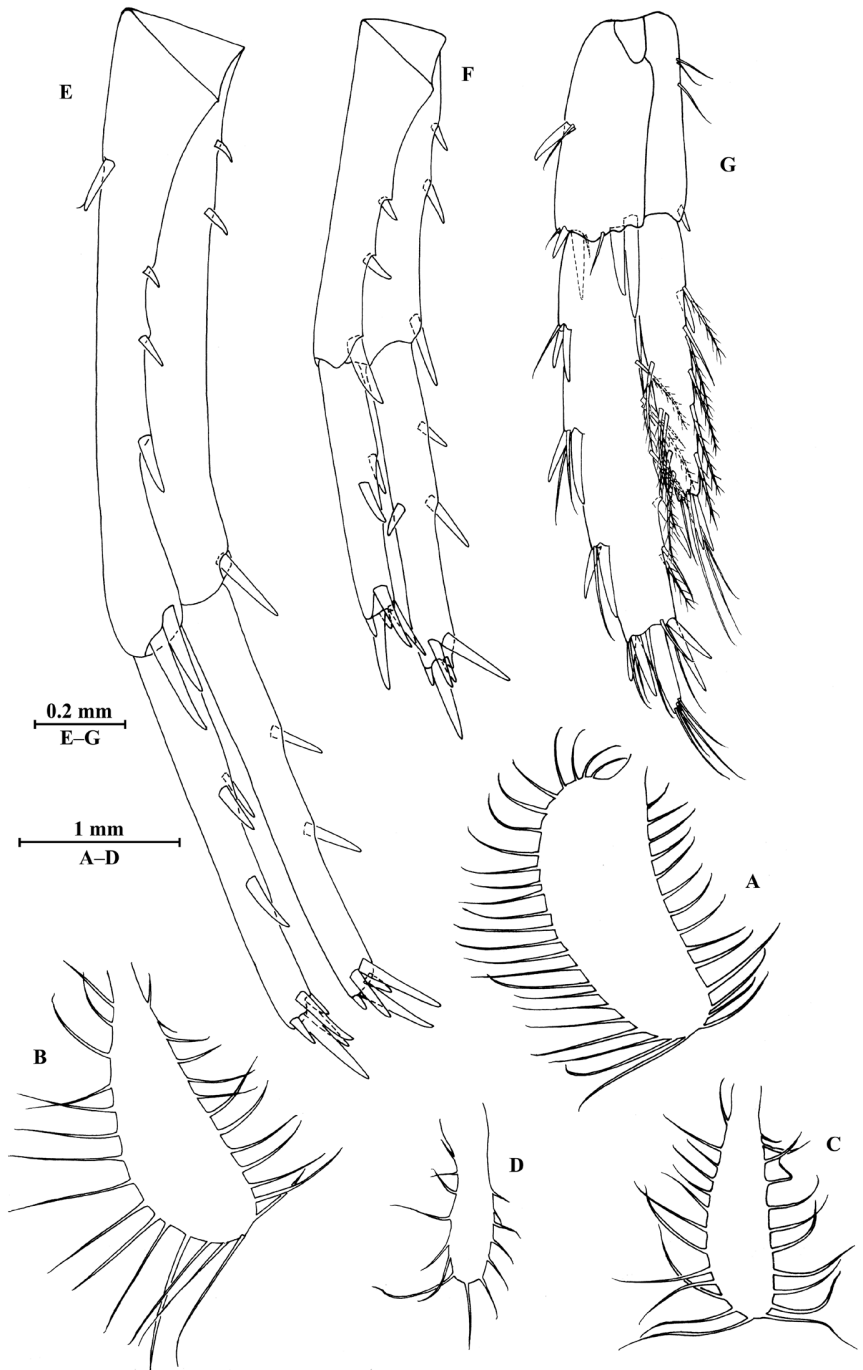
*Gammarus zhigangi* Hou & Li, sp. n., female paratype. **A** oostegite of gnathopod II **B** oostegite of pereopod III **C** oostegite of pereopod IV **D** oostegite of pereopod V **E** uropod I **F** uropod II **G** uropod III.

Figs 14 , 15 , 16 , 17 , 18 , 19 , 20

##### Material examined.

Holotype: male (IZCAS-I-A1424-1), 9.1 mm, Tiantai Mountain National Forest Park (107.05°E, 33.25°N), altitude 865 m, Hanzhong City, Shaanxi Province, China, October 25, 2013, collected by Zhigang Chen. Paratype: female (IZCAS-I-A1424-2), 10.9 mm, same data as holotype.

##### Etymology.

The new species is named after Mr. Zhigang Chen who extensively collected gammarids from China; noun (name) in genitive case.

##### Diagnosis.

Antenna II calceoli present in male; merus of pereopod III with long, straight setae on posterior margin; inner ramus of uropod III reaching 0.6 times the length of outer ramus, outer ramus with no plumose setae on outer margin but with a row of plumose setae on inner margin.

##### Description of holotype male

(IZCAS-I-A1424-1). 9.1 mm.

***Head*** (Fig. 14A): eyes reniform, inferior antennal sinus deep, lateral cephalic lobe rounded.

*Antenna I* (Fig. 14B, C): peduncle articles I–III in length ratio 1.0: 0.6: 0.3, with distal setae; flagellum with 24 articles, articles V–XXII with aesthetascs; accessory ﬂagellum with three articles; both primary and accessory ﬂagella with short distal setae.

*Antenna II* (Fig. 14D, E): peduncle articles III–V in length ratio 1.0: 2.8: 2.7, article IV of peduncle with lateral setae and article V of peduncle with clusters of lateral and medial setae; flagellum with ten articles, with setae along ventral margin; articles III–VI with calceoli.

*Upper lip* (Fig. 14F): ventral margin rounded, bearing short minute setae.

*Mandible* (Fig. 14H, I): left mandible incisor with five teeth; lacinia mobilis with four teeth; spine row with five pairs of plumose setae; articles I–III of palp in length ratio 1.0: 3.1: 2.7, second article of palp with 12 marginal setae, article III with four A-setae, two B-setae, a row of D-setae, and five E-setae apically; incisor of right mandible with four teeth, lacinia mobilis bifurcate, with small teeth.

*Lower lip* (Fig. 14G): inner lobes lacking, outer lobes covered with thin setae.

*Maxilla I* (Fig. 14J, K): asymmetrical, left inner plate with 15 plumose setae on medial margin; outer plate with 11 robust serrated apical spines, each spine with small teeth; second article of left palp with six slender spines and one seta apically; second article of right palp with five stout spines, one stiff seta and one slender spine.

*Maxilla II* (Fig. 14L): inner plate with 15 plumose facial setae in an oblique row; inner and outer plates with long setae apically.

*Maxilliped* (Fig. 14M): inner plate with three stout apical spines and one subapical spine, 15 plumose setae along lateral margin; outer plate bearing a row of 11 blade spines and three plumose setae apically; article IV of palp hooked, with three setae at hinge of unguis.

***Pereon.*** *Gnathopod I* (Fig. 15A, B): coxal plate bearing three setae and one seta on anterior and posterior margins, respectively; basis with long setae on anterior and posterior margins; carpus 1.3 times as long as wide, 0.7 times as long as propodus, ventral margin bearing three clusters of setae; propodus oval, palm with one medial spine and 13 spines on posterior margin and surface; dactylus with one seta on outer margin.

*Gnathopod II* (Fig. 15C, D): coxal plate bearing four setae and one seta on anterior and posterior margins, respectively; basis with long setae on anterior and posterior margins; carpus 1.7 times as long as wide, 0.8 times as long as propodus, bearing six clusters of setae along ventral margin, two clusters of setae on dorsal margin; propodus subrectangular, palm margin with one medial spine and five spines on lateral posterodistal corner; dactylus with one seta on outer margin.

*Pereopod III* (Fig. 16A, B): coxal plate bearing three setae on anterior margin and one seta on posterior margin; basis elongated, with short setae along anterior margin and long setae along posterior margin; merus with long straight setae on posterior margin and two single spines on anterior margin, anterodistal corner with one spine accompanied by two setae; carpus with straight setae on posterior margin; propodus with three spines accompanied by setae on posterior margin and two spines on posterodistal corner; dactylus with one plumose seta on anterior margin, and two setae at hinge of unguis.

*Pereopod IV* (Fig. 16C, D): coxal plate concave, bearing three setae on anterior margin and six setae on posterior margin; basis with two short setae on anterodistal corner and long setae along posterior margin; merus with clusters of setae on posterior margin and one spine on anterior margin, anterodistal corner with one spine accompanied by two setae; carpus with three pairs of spines accompanied by setae on posterior margin, anterodistal corner with one spine accompanied by three setae; propodus with three single spines accompanied by setae on posterior margin and two spines on posterodistal corner; dactylus with one plumose seta on anterior margin, and two setae at hinge of unguis.

*Pereopod V* (Fig. 16E, F): coxal plate bearing one seta on anterior margin and two setae on posterior margin; basis with two pairs of setae and six spines accompanied by fine setae on anterior margin, anterodistal corner with one spine accompanied by setae, posterior margin with a row of 11 setae; merus with one spine accompanied by setae on anterior margin and a pair of spines on posterior margin, anterodistal and posterodistal corners with two spines accompanied by setae each; carpus and propodus with groups of spines on anterior margin; dactylus with one plumose seta on posterior margin, and two setae at hinge of unguis.

*Pereopod VI* (Figs 16G, H): coxal plate bearing one seta on anterior margin and two setae on posterior margin; basis with four simple setae and five spines accompanied by setae on anterior margin, anterodistal corner with three spines and two fine setae, posterior margin with a row of ten fine setae; merus with three pairs of spines on anterior margin and one spine on posterior margin, anterodistal and posterodistal corners with four spines each; carpus with two groups of spines accompanied by setae on anterior and posterior margins each, anterodistal corner with five spines accompanied by one fine seta and posterodistal corner with five spines; propodus with four groups of spines on anterior margin; dactylus with one plumose seta on posterior margin, and two setae at hinge of unguis.

*Pereopod VII* (Fig. 16I, J): coxal plate bearing two setae on posterior margin; basis with four long simple setae and four spines on anterior margin, anterodistal corner with two spines and two fine setae, posterior margin with a row of 11 setae; merus with two groups of spines on anterior margin and a pair of spines on posterior margin, anterodistal corner with four spines accompanied by two setae and posterodistal corner with three spines accompanied by one seta; carpus with three groups of spines on anterior margin and two groups of spines on posterior margin, anterodistal corner with three spines accompanied by one seta and posterodistal corner with three spines; propodus with three groups of spines on anterior margin; dactylus with one plumose seta on posterior margin, and two setae at hinge of unguis.

*Coxal gills*: coxal gill of gnathopod II and gills of pereopods III to V a little longer than bases; gill of pereopod VI a little shorter than basis; gill of pereopod VII smallest, approx. half the length of basis.

***Pleon.*** *Epimeral plates* (Fig. 15E–G): plate I ventrally rounded, bearing three setae and two spines on anteroventral margin and four tiny setae on posterior margin; plate II with two spines on ventral margin and five tiny setae on posterior margin, posterodistal corner subacute; plate III with three spines on ventral margin and five tiny setae on posterior margin, posterodistal corner subacute.

*Pleopods I–III* (Fig. 17A–C): similar, peduncle with two retinacula accompanied by one plumose seta; outer ramus slightly shorter than inner ramus, both inner and outer rami fringed with plumose setae.

***Urosome.*** *Urosomites* (Fig. 15H): urosomite I with one-one-one-one spines accompanied by setae on dorsal margin; urosomite II with one-one-one-two spines accompanied by setae on dorsal margin; urosomite III with one spine accompanied by two setae on each side and three setae on dorsal margin.

*Uropods I–III* (Fig. 17D–F): uropod I peduncle with one basofacial spine, two spines on inner and outer margins each, inner and outer distal corners with one and two spines, respectively; inner ramus with two spines on inner margin; outer ramus with one spine on inner and outer margins each; both rami with five terminal spines. Uropod II peduncle with one spine on inner and outer margins each, and with one distal spine on each corner; inner ramus with two spines on inner margin and one spine on outer margin; outer ramus with one spine on inner and outer margins each; both rami with five terminal spines. Uropod III peduncle with one spine accompanied by one seta on surface and six distal spines; inner ramus 1.4 times as long as peduncle, reaching 0.6 times the length of outer ramus, with two spines accompanied by eight plumose setae and two simple setae on inner margin, five plumose setae on outer margin, and two distal spines accompanied by setae; proximal article of outer ramus with six spines accompanied by simple setae on outer margin, with 13 plumose setae on inner margin, terminal article with simple setae, a little longer than adjacent spines.

*Telson* (Fig. 17G): deeply cleft, approx. as long as wide; each lobe with clusters of setae on surface, bearing two distal spines accompanied by setae.

##### Description of paratype female

(IZCAS-I-A1424-2). 10.9 mm.

***Pereon.*** *Gnathopod I* (Fig. 18A, B): coxal plate bearing three and one setae on anterior and posterior margins, respectively; basis with setae on anterior and posterior margins; propodus oval, palm with seven spines on posterior margin, bearing long setae along anterior and posterior margins; dactylus with one seta on outer margin.

*Gnathopod II* (Fig. 18C, D): coxal plate bearing five and one setae on anterior and posterior margins, respectively; basis with setae on anterior and posterior margins; propodus subrectangular, palm margin with two stout spines and three stiff spines on posterodistal corner, bearing long setae along anterior and posterior margins; dactylus with one seta on outer margin.

*Pereopods III and IV* (Fig. 19A, B): with more setae on posterior margins than those of male.

*Pereopods V–VII* (Fig. 19C–E): similar to those of male, but with more setae on anterior margins of pereopods V–VI.

*Oostegite* (Fig. 20A–D): oostegite of gnathopod II broad, with marginal setae, oostegites of pereopods III and IV elongated, oostegite of pereopod V smallest.

***Urosome.*** *Uropods I–III* (Fig. 20E–G): uropods I and II similar to those of male. Uropod III peduncle with one spine accompanied by one seta on surface and five distal spines; inner ramus 1.3 times as long as peduncle, reaching 0.6 times the length of outer ramus, with one spine accompanied by five plumose setae and one simple seta on inner margin and five plumose setae on outer margin; proximal article of outer ramus with three pairs of spines accompanied by simple setae on outer margin, with one spine accompanied by four plumose setae and three simple setae on inner margin, terminal article a little longer than adjacent spines.

*Telson* (Fig. 19F): cleft, approx. as long as wide; each lobe with one spine accompanied by two setae and two clusters of three setae on surface, bearing two distal spines accompanied by setae.

##### Habitat.

Specimens were collected from a geyser in Tiantai Mountain National Forest Park. The geyser is influenced by the formation of cavities in the deep strata. When the groundwater is filled with cavities, the geyser will erupt from the rock cracks. The intermittent geyser is considered as person’s breathing, therefore it is known as a breathing spring. This park is located in the middle of the southern slope of Qinling with lush forests; the topography is full of deep valleys and steep mountains.

##### Remarks.

The new species of *Gammarus zhigangi* Hou & Li, sp. n. is most similar to *Gammarus qinling* Hou & Li, sp. n. in antenna II calceoli present; merus of pereopod III with straight setae on posterior margin; and epimeral plates II and III blunt on posterodistal corners. *Gammarus zhigangi* Hou & Li, sp. n. differs from *Gammarus qinling* Hou & Li, sp. n. (*Gammarus qinling* in parentheses) by pereopod V of male and female with more setae on anterior margin of merus; and uropod III inner ramus 0.6 times the length of outer ramus (0.5 times), outer margin of outer ramus with no plumose setae (with plumose setae).

The new species of *Gammarus zhigangi* Hou & Li, sp. n. is similar to *G. preciosus* Wang, Hou & Li, 2009 in antenna II calceoli present; uropod III without plumose setae on outer margin of outer ramus, and terminal article longer than adjacent spines; and telson long than wide. *Gammarus zhigangi* Hou & Li, sp. n. can be distinguished from *G. preciosus* Wang, Hou & Li, 2009 (*G. preciosus* in parentheses) in epimeral plate I with three setae and two spines on anteroventral margin (with eight long setae on anteroventral margin); epimeral plate III with five setae on posterior margin (with 11 setae on posterior margin); and uropod III inner ramus 0.6 times the length of outer ramus (inner ramus 0.4 times the length of outer ramus).

This new species can be distinguished from *G. murarius* Hou & Li, 2004 (*G. murarius* in parentheses) by the following characters: merus and carpus of pereopod III with straight setae on posterior margins (with long curled setae); epimeral plate I bearing three setae and two spines on anteroventral margin (only with four setae); and uropod III without plumose setae on outer margin of outer ramus (with plumose).

#### Gammarus jidutanxian

Taxon classificationAnimaliaAmphipodaGammaridae

Hou & Li sp. n.

http://zoobank.org/3CB909C4-CF89-4BB6-B94B-580A94E6147C

**Figure 21.**
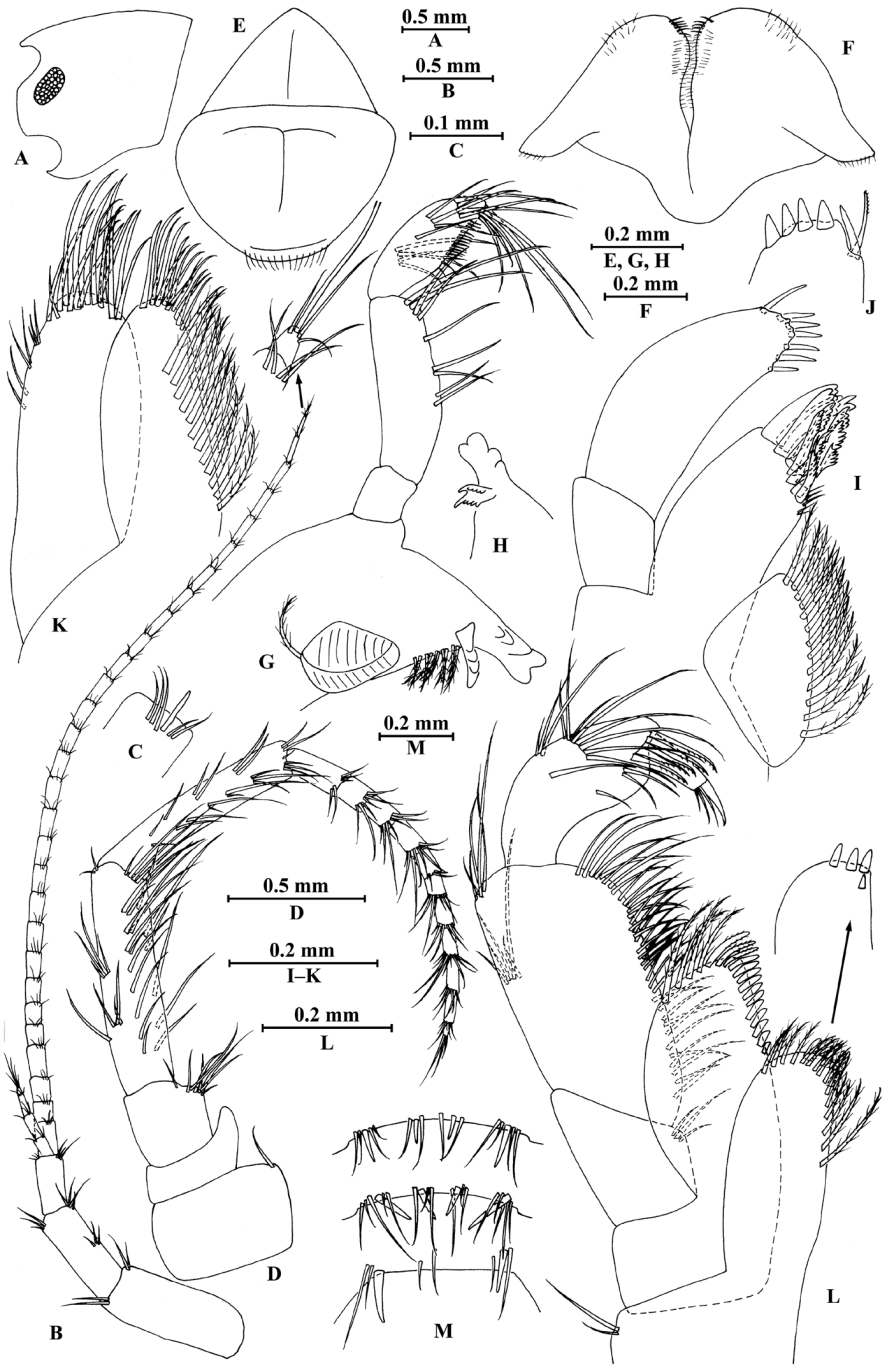
*Gammarus jidutanxian* Hou & Li, sp. n., male holotype. **A** head **B** antenna I **C** flagellar article of antenna I with aesthetasc **D** antenna II **E** upper lip **F** lower lip **G** left mandible **H** incisor and lacinia mobilis of right mandible **I** left maxilla I **J** distal part of palp article II of right maxilla I **K** maxilla II **L** maxilliped **M** dorsal margins of urosomites I–III.

**Figure 22.**
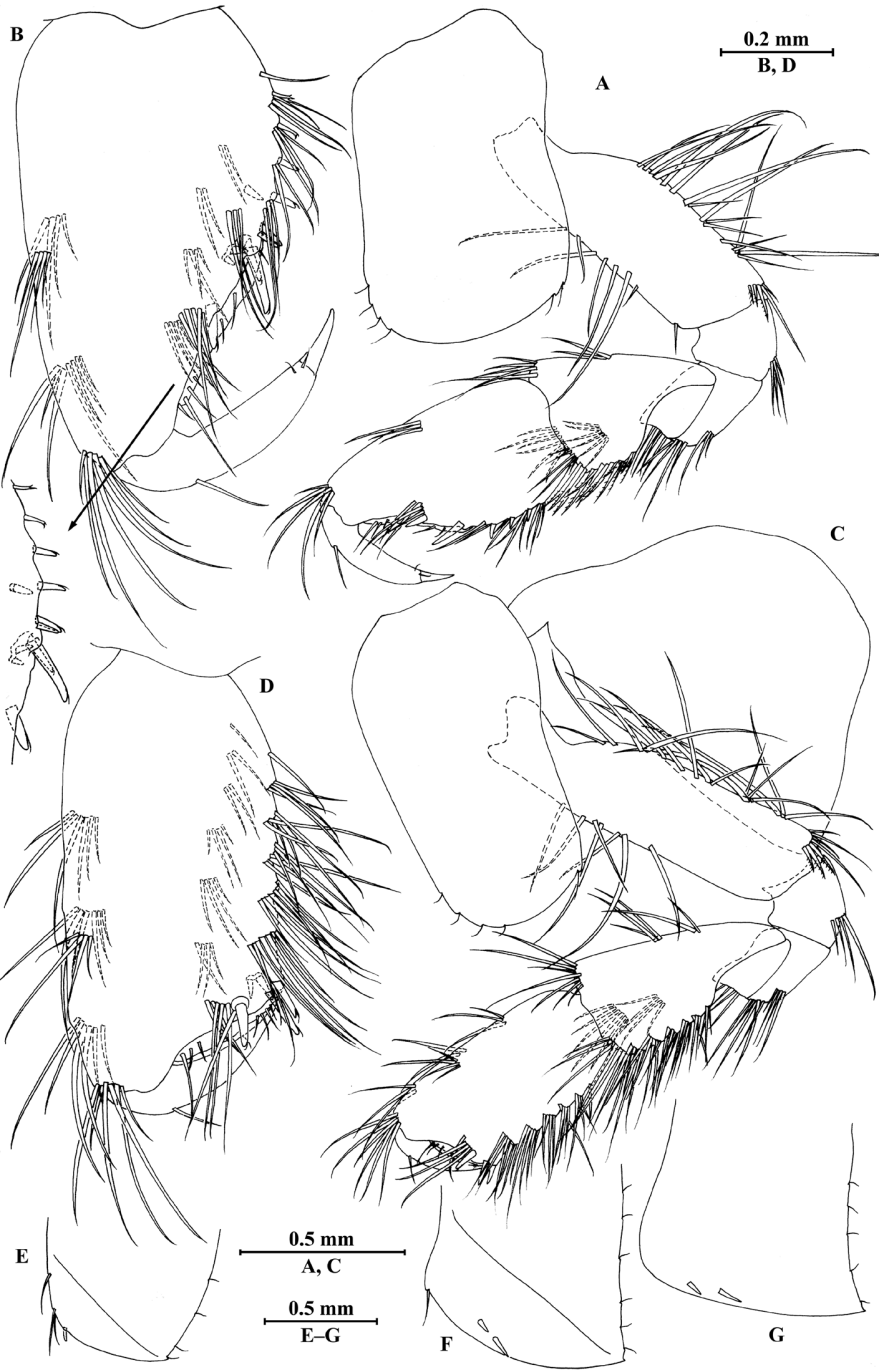
*Gammarus jidutanxian* Hou & Li, sp. n., male holotype. **A** gnathopod I **B** propodus and dactylus of gnathopod I **C** gnathopod II **D** propodus and dactylus of gnathopod II **E** epimeral plate I **F** epimeral plate II **G** epimeral plate III.

**Figure 23.**
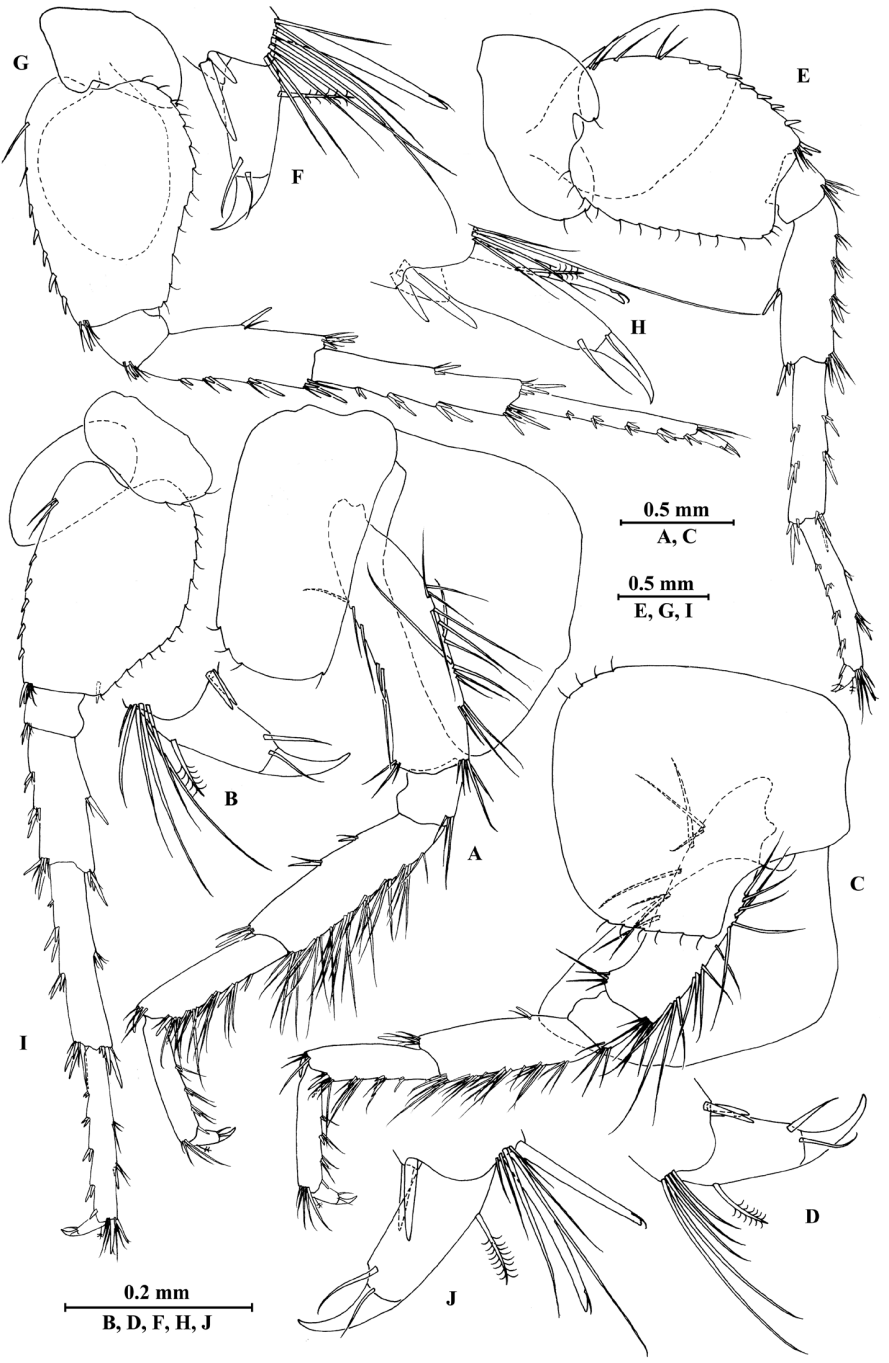
*Gammarus jidutanxian* Hou & Li, sp. n., male holotype. **A** pereopod III **B** dactylus of pereopod III **C** pereopod IV **D** dactylus of pereopod IV **E** pereopod V **F** dactylus of pereopod V **G** pereopod VI **H** dactylus of pereopod VI **I** pereopod VII **J** dactylus of pereopod VII.

**Figure 24.**
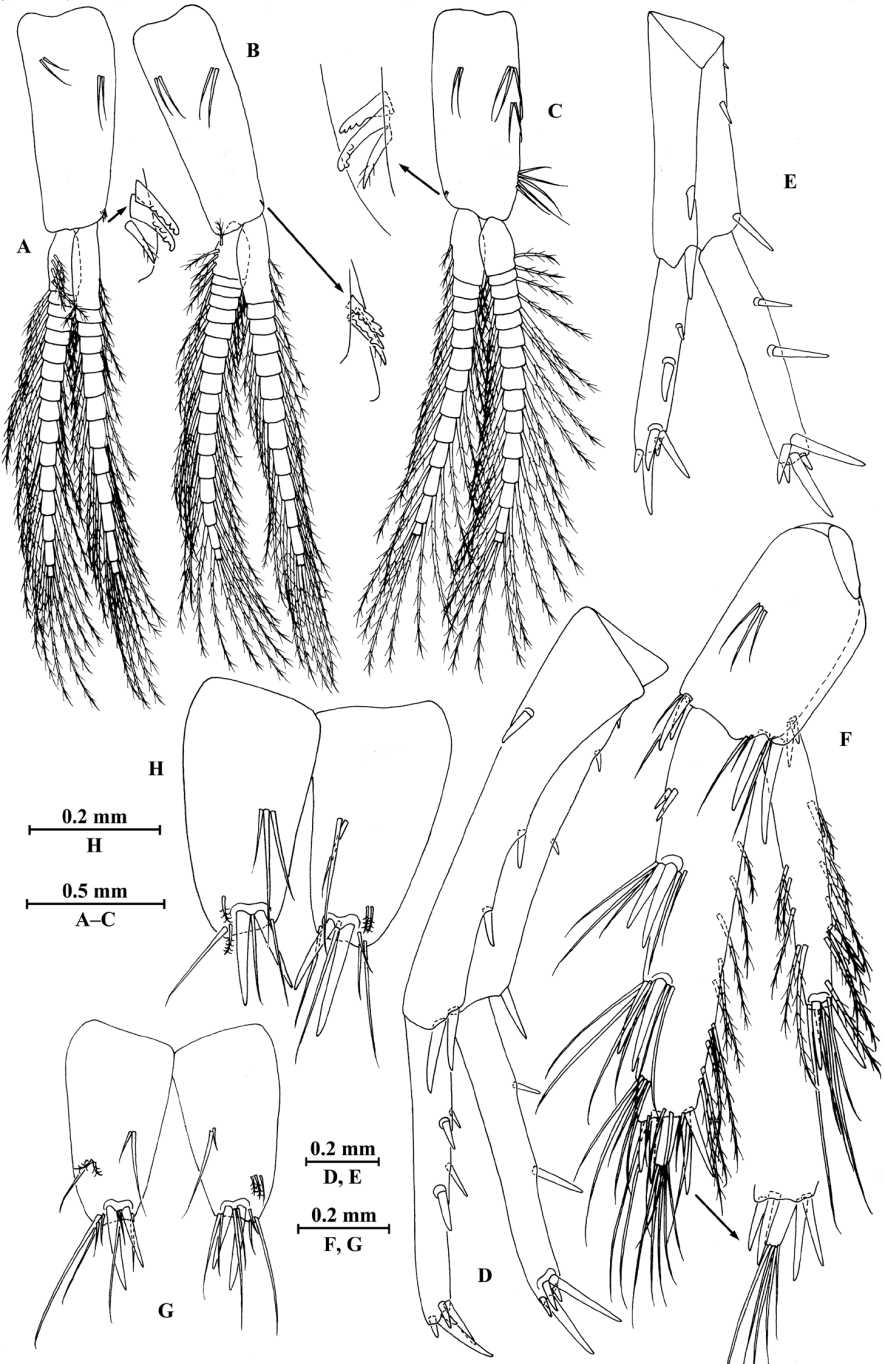
*Gammarus jidutanxian* Hou & Li, sp. n., **A–G** male, holotype; **H** female, paratype. **A** pleopod I **B** pleopod II **C** pleopod III **D** uropod I **E** uropod II **F** uropod III **G** telson **H** telson.

**Figure 25.**
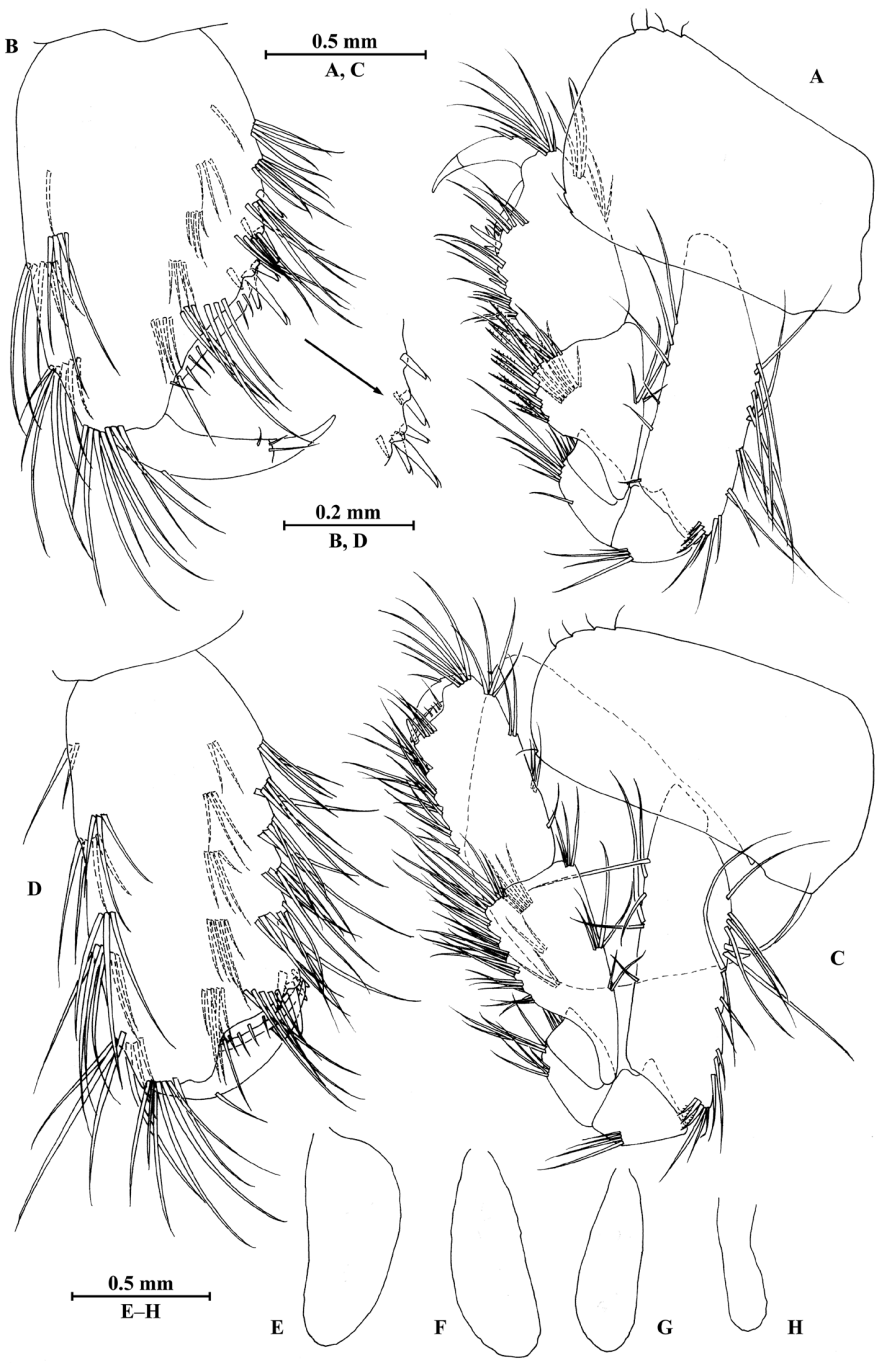
*Gammarus jidutanxian* Hou & Li, sp. n., female paratype. **A** gnathopod I **B** propodus and dactylus of gnathopod I **C** gnathopod II **D** propodus and dactylus of gnathopod II **E** oostegite of gnathopod II **F** oostegite of pereopod III **G** oostegite of pereopod IV **H** oostegite of pereopod V.

**Figure 26.**
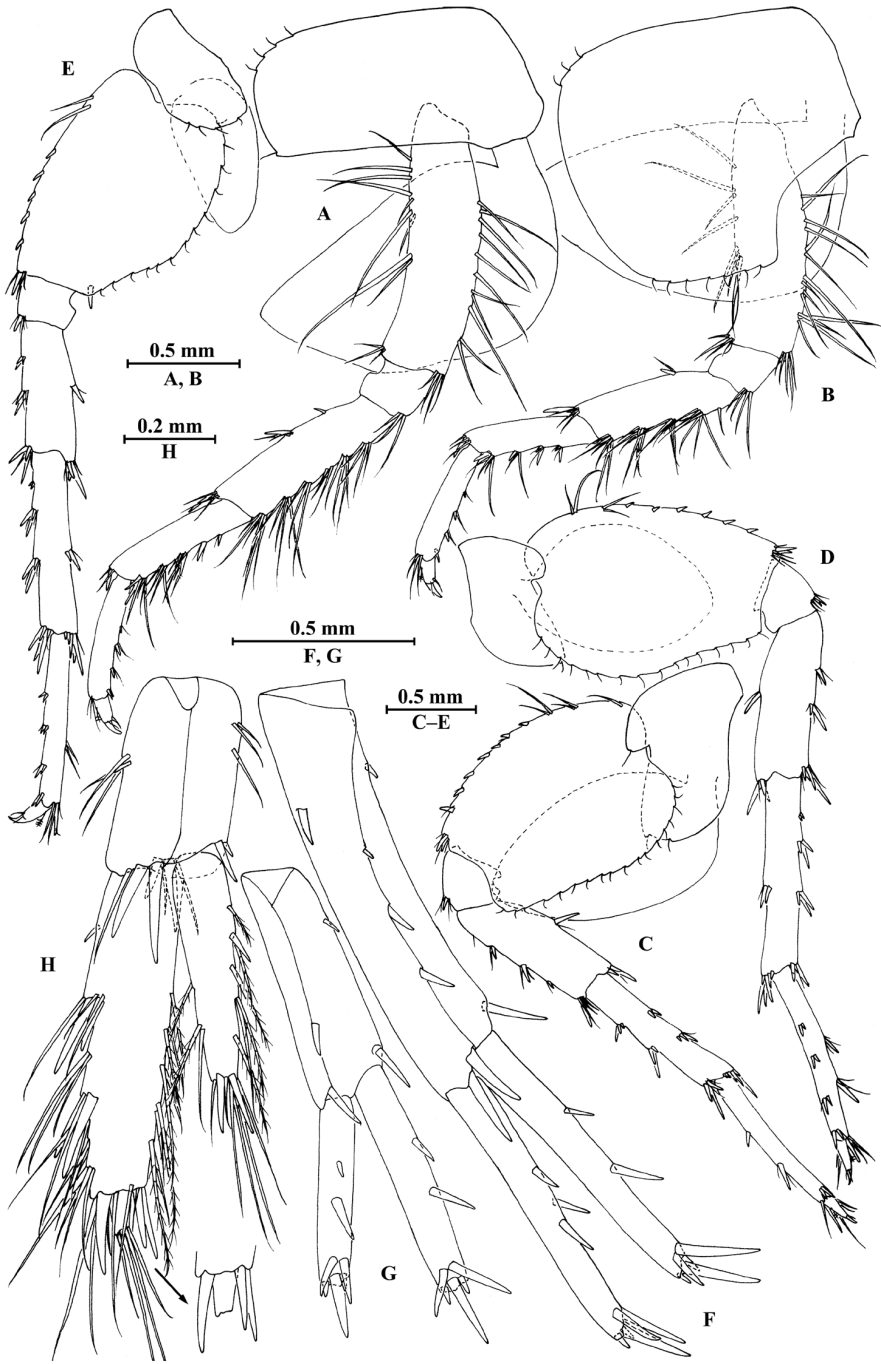
*Gammarus jidutanxian* Hou & Li, sp. n., female paratype. **A** pereopod III **B** pereopod IV **C** pereopod V **D** pereopod VI **E** pereopod VII **F** uropod I **G** uropod II **H** uropod III.

Figs 21 , 22 , 23 , 24 , 25 , 26

##### Material examined.

Holotype: male (IZCAS-I-A1439-1), 8.2 mm, Langao County (108.91°E, 32.29°N), altitude 529 m, Ankang City, Shaanxi Province, China, October 28, 2013, collected by Yunchun Li and Jincheng Liu. Paratype: female (IZCAS-I-A1439-2), 9.8 mm, same data as holotype. Paratype: male (IZCAS-I-A1804), 8.5 mm, Huiwan Town (109.84°E, 32.15°N), Zhuxi County, Shiyan City, Hubei Province, August 28, 2015, collected by Chunjiang Sang.

##### Etymology.

The species name is a Chinese phrase, “*jidutanxian*”, meaning “adventure exploration”, in honour of Mr. Chunjiang Sang extensively exploring karst biota in southern China; noun in apposition.

##### Diagnosis.

Antenna II peduncle with long setae, calceoli absent; epimeral plate III with subacute posterodistal corner; uropod III inner ramus reaching 0.6 times the length of outer ramus, outer ramus with no plumose setae on outer margin, terminal article of outer ramus shorter than adjacent spines; each lobe of telson with plumose setae on surface.

##### Description of holotype male

(IZCAS-I-A1439-1). 8.2 mm.

***Head*** (Fig. 21A): eyes oval, inferior antennal sinus deep, lateral cephalic lobe rounded.

*Antenna I* (Fig. 21B, C): peduncle articles I–III in length ratio 1.0: 0.7: 0.4, with distal setae; flagellum with 30 articles, articles VII–XXV with aesthetascs; accessory ﬂagellum with four articles; both primary and accessory ﬂagella with short distal setae.

*Antenna II* (Fig. 21D): peduncle articles III–V in length ratio 1.0: 2.7: 2.3, articles IV–V with long setae along anterior and posterior margins; flagellum with 11 articles, each article with long setae; calceoli absent.

*Upper lip* (Fig. 21E): ventral margin rounded, bearing short minute setae.

*Mandible* (Fig. 21G, H): left mandible incisor with five teeth; lacinia mobilis with four teeth; spine row with five pairs of plumose setae; articles I–III of palp in length ratio 1.0: 3.9: 2.5, second article with nine marginal setae, article III with four A-setae, eight B-setae, a row of D-setae, and five E-setae apically; incisor of right mandible with four teeth, lacinia mobilis bifurcate, with small teeth.

*Lower lip* (Fig. 21F): inner lobes lacking, outer lobes covered with thin setae.

*Maxilla I* (Figs 21I, J): asymmetrical, left inner plate with 13 plumose setae on medial margin; outer plate with 11 robust serrated apical spines, each spine with small teeth; second article of left palp with seven slender spines apically; second article of right palp with four stout spines, one stiff seta and one slender spine.

*Maxilla II* (Fig. 21K): inner plate with 12 plumose setae in an oblique row; inner and outer plates with long setae apically.

*Maxilliped* (Fig. 21L): inner plate with three stout apical spines and one subapical spine, 15 plumose setae along lateral margin; outer plate bearing a row of 14 blade spines and four plumose setae apically; article IV of palp hooked, with three setae at hinge of unguis.

***Pereon.*** *Gnathopod I* (Fig. 22A, B): coxal plate bearing four setae and two setae on anterior and posterior margins, respectively; basis with setae on anterior and posterior margins; carpus 1.4 times as long as wide, 0.8 times as long as propodus, posterior margin bearing four clusters of short setae; propodus oval, palm with one medial spine and 11 spines and clusters of simple setae on posterior margin and surface; dactylus with one seta on outer margin.

*Gnathopod II* (Fig. 22C, D): coxal plate bearing four setae and one seta on anterior and posterior margins, respectively; basis with setae on anterior and posterior margins; carpus 2.0 times as long as wide, approx. as long as propodus, bearing seven clusters of long setae along ventral margin, three clusters of setae on dorsal margin; propodus subrectangular, palm margin with one medial spine and four spines on posterodistal corner; dactylus with one seta on outer margin.

*Pereopod III* (Fig. 23A, B): coxal plate bearing three setae on anterior margin and one seta on posterior margin; basis elongated, with setae along anterior and posterior margins; merus with long straight setae on posterior margin and two spines accompanied by two setae on anterior margin, anterodistal corner with one spine accompanied by three setae; carpus with four clusters of spines accompanied by straight setae on posterior margin; propodus with three clusters of spines accompanied by setae on posterior margin and two spines on posterodistal corner; dactylus with one plumose seta on anterior margin, and two setae at hinge of unguis.

*Pereopod IV* (Fig. 23C, D): coxal plate concave, bearing four setae on anterior margin and six setae on posterior margin; basis with long setae along anterior and posterior margins; merus with clusters of setae on posterior margin and one spine accompanied by one seta on anterior margin, anterodistal corner with one spine accompanied by four setae; carpus and propodus with three groups of spines accompanied by setae on posterior margins; dactylus with one plumose seta on anterior margin, and two setae at hinge of unguis.

*Pereopod V* (Fig. 23E, F): coxal plate bearing one seta on anterior margin and two setae on posterior margin; basis with five setae and seven spines accompanied by fine setae on anterior margin, anterodistal corner with two spines accompanied by setae, posterior margin with a row of 14 setae; merus with two spines accompanied by setae on anterior margin and one spine accompanied by seta on posterior margin, anterodistal and posterodistal corners with one and two spines accompanied by setae respectively; carpus and propodus with groups of spines on anterior margins; dactylus with one plumose seta on posterior margin, and two setae at hinge of unguis.

*Pereopod VI* (Fig. 23G, H): coxal plate bearing one seta on posterior margin; basis with two setae and five spines accompanied by one seta on anterior margin, anterodistal corner with one spine and three fine setae, posterior margin with a row of 14 fine setae; merus to propodus with groups of spines accompanied by short setae on anterior margins; dactylus with one plumose seta on posterior margin, and two setae at hinge of unguis.

*Pereopod VII* (Fig. 23I, J): coxal plate bearing three setae on posterior margin; basis with two simple setae and five spines on anterior margin, anterodistal corner with two spines accompanied by fine setae, posterior margin with a row of 15 setae, and with one spine on inner surface; merus to propodus with groups of spines accompanied by short setae on anterior margins; dactylus with one plumose seta on posterior margin, and two setae at hinge of unguis.

*Coxal gills*: coxal gills of gnathopod II and pereopod III a little shorter than bases; gill of pereopod IV longer than basis; gills of pereopods V and VI shorter than bases; gill of pereopod VII smallest, less than half the length of basis.

***Pleon.*** *Epimeral plates* (Fig. 22E–G): plate I ventrally rounded, bearing three setae and one spine on anteroventral margin and four setae on posterior margin; plate II with one seta and two spines on ventral margin and six setae on posterior margin, posterodistal corner blunt; plate III with two spines on ventral margin and five setae on posterior margin, posterodistal corner subacute.

*Pleopods I–III* (Fig. 24A–C): similar, peduncle with two retinacula accompanied by one seta; outer ramus as long as inner ramus, both rami fringed with plumose setae.

***Urosome.*** *Urosomites* (Fig. 21M): urosomites I and II with one-one-one-one spines accompanied by setae on dorsal margins; urosomite III with one spine accompanied by two setae on each side and two setae on dorsal margin.

*Uropods I–III* (Figs 24D–F): uropod I peduncle with one basofacial spine, three and two spines on inner and outer margins, respectively, inner and outer distal corners with one and two spines, respectively; inner ramus with two spines on inner margin; outer ramus with two spines on inner and outer margins each; both rami with five terminal spines. Uropod II peduncle with two spines on inner margin and one spine on outer margin, and with one distal spine on each corner; inner ramus with two spines on inner margin; outer ramus with two spines on outer margin; both rami with five terminal spines. Uropod III peduncle with three setae on surface and six distal spines; inner ramus 1.2 times as long as peduncle, reaching 0.6 times the length of outer ramus, with two spines accompanied by seven plumose setae on inner margin, five plumose setae on outer margin, and two distal spines accompanied by setae; proximal article of outer ramus with three pairs of spines accompanied by simple setae on outer margin, with ten plumose setae on inner margin, terminal article with simple setae, shorter than adjacent spines.

*Telson* (Fig. 24G): deeply cleft, approx. as long as wide; each lobe with three simple setae and two plumose setae on surface, bearing two distal spines accompanied by setae.

##### Description of paratype female

(IZCAS-I-A1439-2). 9.8 mm.

***Pereon.*** *Gnathopod I* (Fig. 25A, B): coxal plate bearing four and two setae on anterior and posterior margins, respectively; basis with setae on anterior and posterior margins; propodus oval, palm with seven spines on posterior margin, bearing long setae along anterior and posterior margins; dactylus with one seta on outer margin.

*Gnathopod II* (Fig. 25C, D): coxal plate bearing four and one seta on anterior and posterior margins, respectively; basis with setae on anterior and posterior margins; propodus subrectangular, palm margin with four spines on posterodistal corner, bearing long setae along anterior and posterior margins; dactylus with one seta on outer margin.

*Pereopods III and IV* (Fig. 26A, B): with fewer setae on posterior margins than those of male.

*Pereopods V–VII* (Fig. 26C–E): similar to those of male.

*Oostegite* (Fig. 25E–G): oostegite of gnathopod II broad, oostegites of pereopods III and IV elongated, oostegite of pereopod V smallest.

***Urosome.*** *Uropods I–III* (Fig. 26F–H): uropod I peduncle with one basofacial spine, with one and three spines on inner and outer margins, respectively, with one and two spines on inner and outer corners; inner ramus with two spines on inner margin; outer ramus with one and two spines on inner and outer margins, respectively; both rami with five terminal spines. Uropod II peduncle with one spine on inner and outer margins each, with one distal spine on each corner; inner ramus with two spines on inner margin; outer ramus with two spines on outer margin; both rami with five terminal spines. Uropod III peduncle with setae on surface and six distal spines; inner ramus 1.4 times as long as peduncle, reaching 0.8 times the length of outer ramus, with one spine accompanied by four plumose setae and one simple seta on inner margin and two plumose setae accompanied by one simple setae on outer margin; proximal article of outer ramus with a single spine and two pairs of spines accompanied by simple setae on outer margin, with five plumose setae and four simple setae on inner margin, terminal article shorter than adjacent spines.

*Telson* (Fig. 24H): cleft, approx. as long as wide; each lobe with three or two simple setae and two plumose setae on surface, bearing two distal spines accompanied by setae.

##### Habitat.

This species was collected along the shore of a brook, usually in gravel and decomposing leaves. The type locality is located in a valley of north part of Daba Mountain.

##### Remarks.

The new species of *Gammarus jidutanxian* Hou & Li, sp. n. is most similar to *G. craspedotrichus* Hou & Li, 2002 in antenna II with long setae along peduncular articles and calceoli absent; and outer margin of outer ramus in uropod III with simple setae. It differs from *G. craspedotrichus* (*G. craspedotrichus* in parentheses) in peduncle of uropod I with one basofacial spine (without basofacial spine); inner ramus reaching 0.6 times of outer ramus in uropod III (inner ramus approx. as long as outer ramus); and urosomites with four groups of spines and setae (with two clusters of spines and setae).

The new species of *Gammarus jidutanxian* Hou & Li, sp. n. is most similar to *G. vallecula* Hou & Li, sp. n. in antenna II with long setae on peduncle margin and calceoli absent; pereopods III and IV with straight setae on posterior margin; and urosomites with four groups of spines and setae on dorsal margin. *Gammarus jidutanxian* Hou & Li, sp. n. can be distinguished from *G. vallecula* Hou & Li, sp. n. (*G. vallecula* in parentheses) in uropod III inner ramus reaching 0.6 times the length of outer ramus, terminal article shorter than adjacent spines (inner ramus approx. half the length of outer ramus, terminal article subequal or longer than adjacent spines); and telson as long as wide, with no spines on surface (telson 0.8 times as long as wide, each lobe with one spine accompanied by setae on surface).

*Gammarus jidutanxian* Hou & Li, sp. n. differs from *Gammarus accretus* Hou & Li, 2002a (*G. accretus* in parentheses) by urosomites I and II with one-one-one-one spines accompanied by setae on dorsal margins (with only one group of setae); uropod I peduncle with one basofacial spine (without basofacial spine); and inner ramus of uropod III 0.6 times the length of outer ramus (approx. the same length).

#### Gammarus longdong

Taxon classificationAnimaliaAmphipodaGammaridae

Hou & Li sp. n.

http://zoobank.org/0FF3D2CA-932A-4ABE-B222-22AE4922DC1B

**Figure 27.**
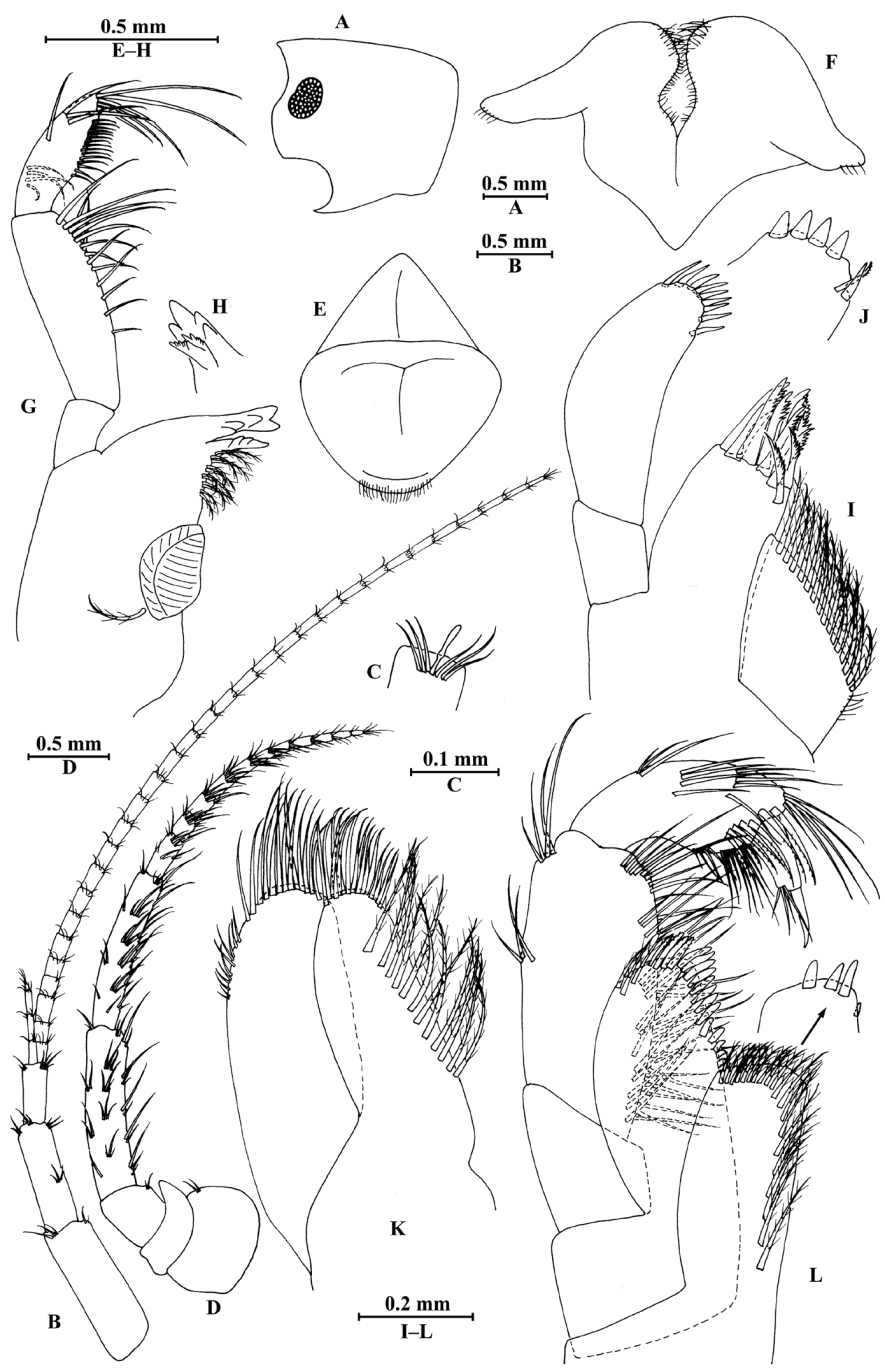
*Gammarus longdong* Hou & Li, sp. n., male holotype. **A** head **B** antenna I **C** flagellar article of antenna I with aesthetasc **D** antenna II **E** upper lip **F** lower lip **G** left mandible **H** incisor and lacinia mobilis of right mandible **I** left maxilla I **J** distal part of palp article II of right maxilla I **K** maxilla II **L** maxilliped.

**Figure 28.**
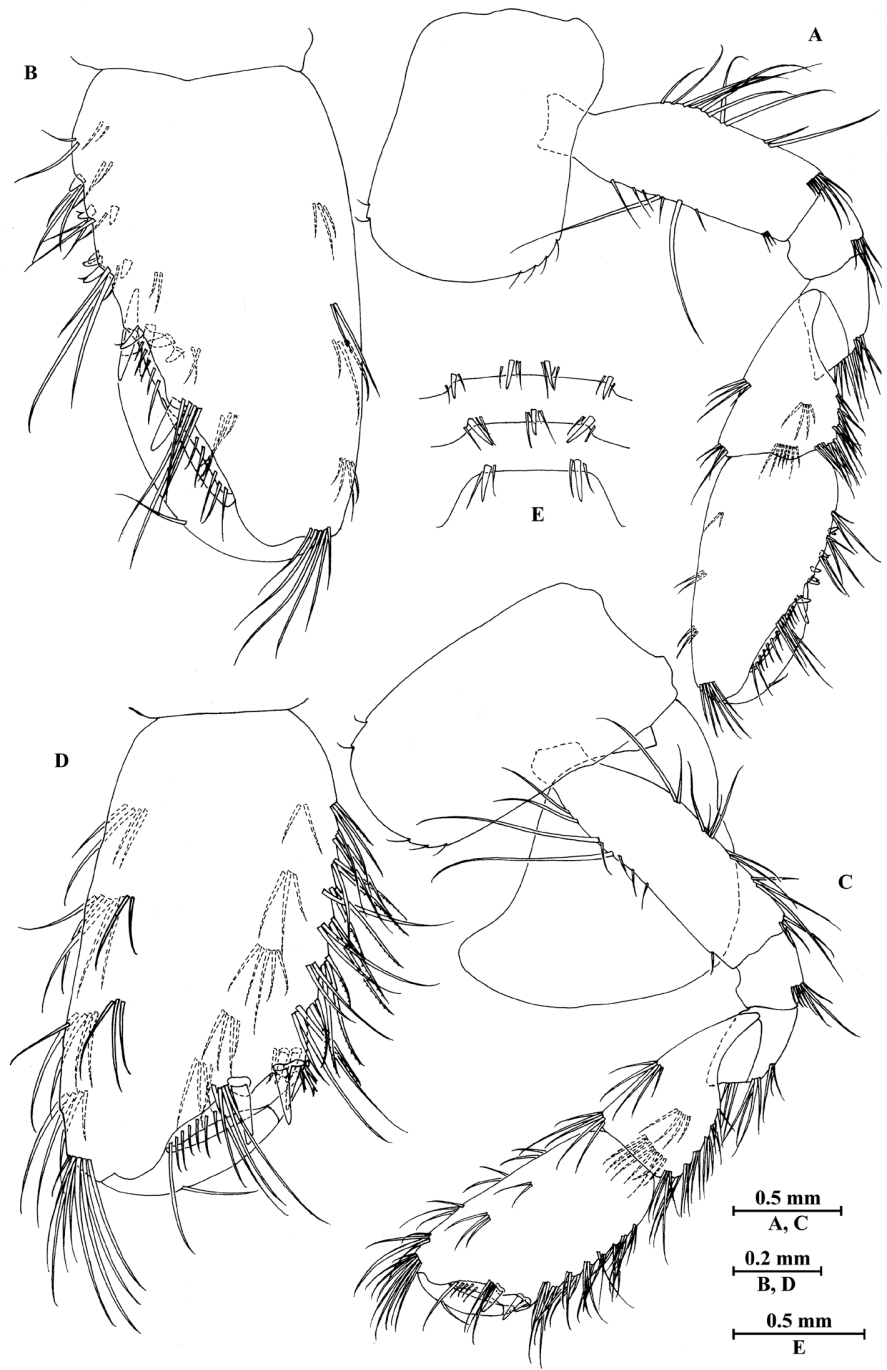
*Gammarus longdong* Hou & Li, sp. n., male holotype. **A** gnathopod I **B** propodus and dactylus of gnathopod I **C** gnathopod II **D** propodus and dactylus of gnathopod II **E** dorsal margins of urosomites I–III.

**Figure 29.**
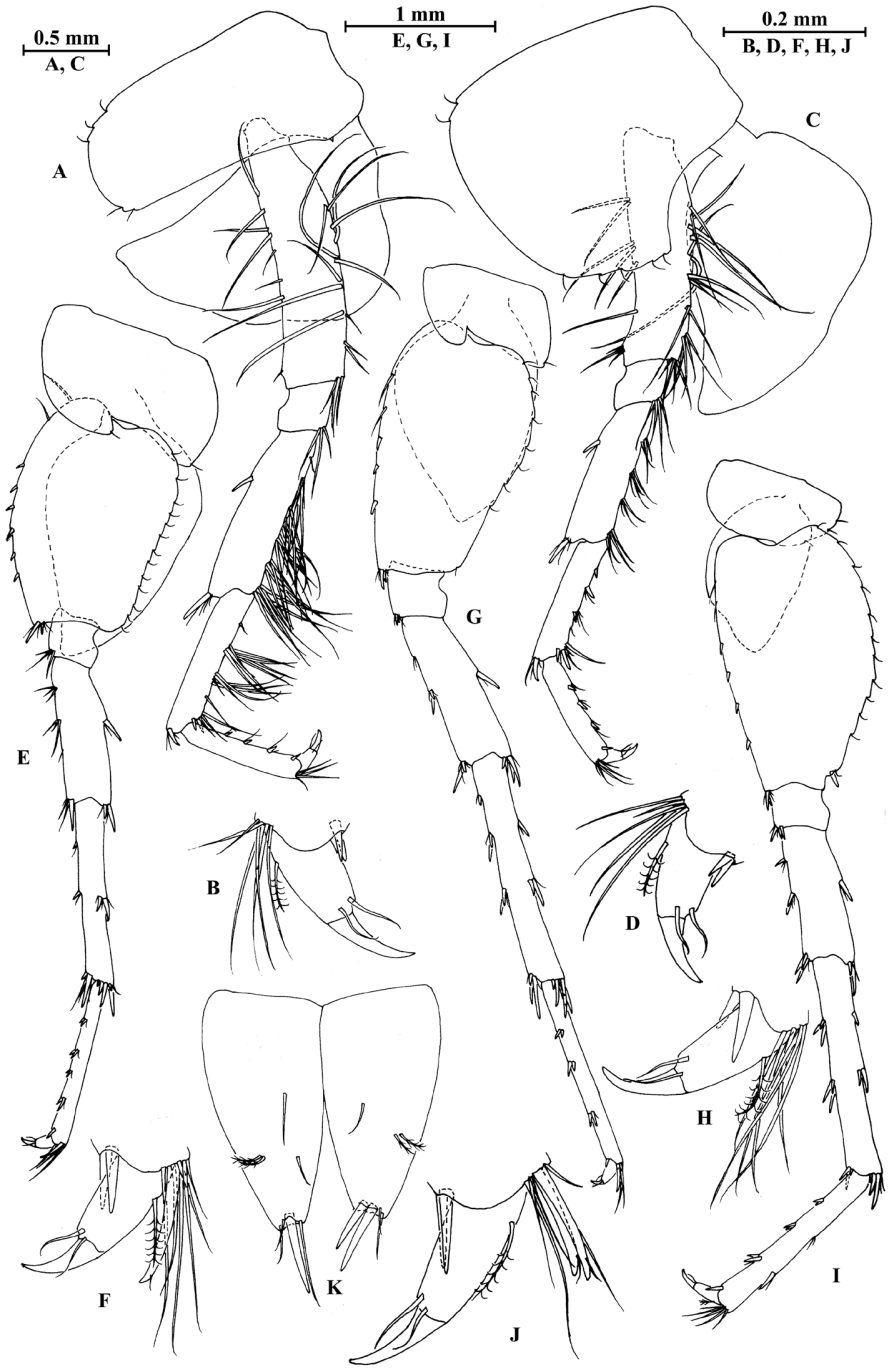
*Gammarus longdong* Hou & Li, sp. n., male holotype. **A** pereopod III **B** dactylus of pereopod III **C** pereopod IV **D** dactylus of pereopod IV **E** pereopod V **F** dactylus of pereopod V **G** pereopod VI **H** dactylus of pereopod VI **I** pereopod VII **J** dactylus of pereopod VII **K** telson.

**Figure 30.**
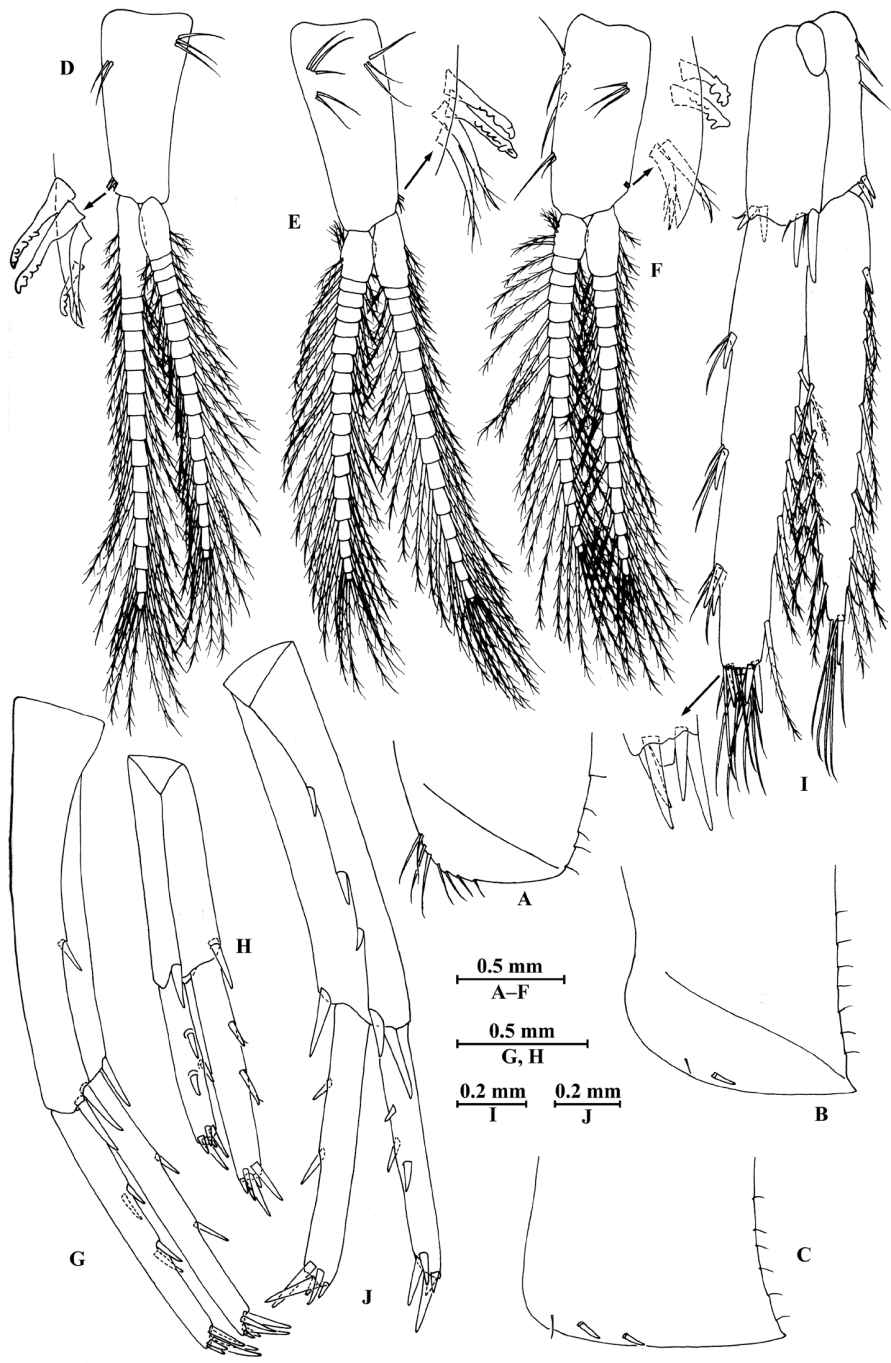
*Gammarus longdong* Hou & Li, sp. n., **A–I** male, holotype; **J** female, paratype. **A** epimeral plate I **B** epimeral plate II **C** epimeral plate III **D** pleopod I **E** pleopod II **F** pleopod III **G** uropod I **H** uropod II **I** uropod III **J** uropod I (right).

**Figure 31.**
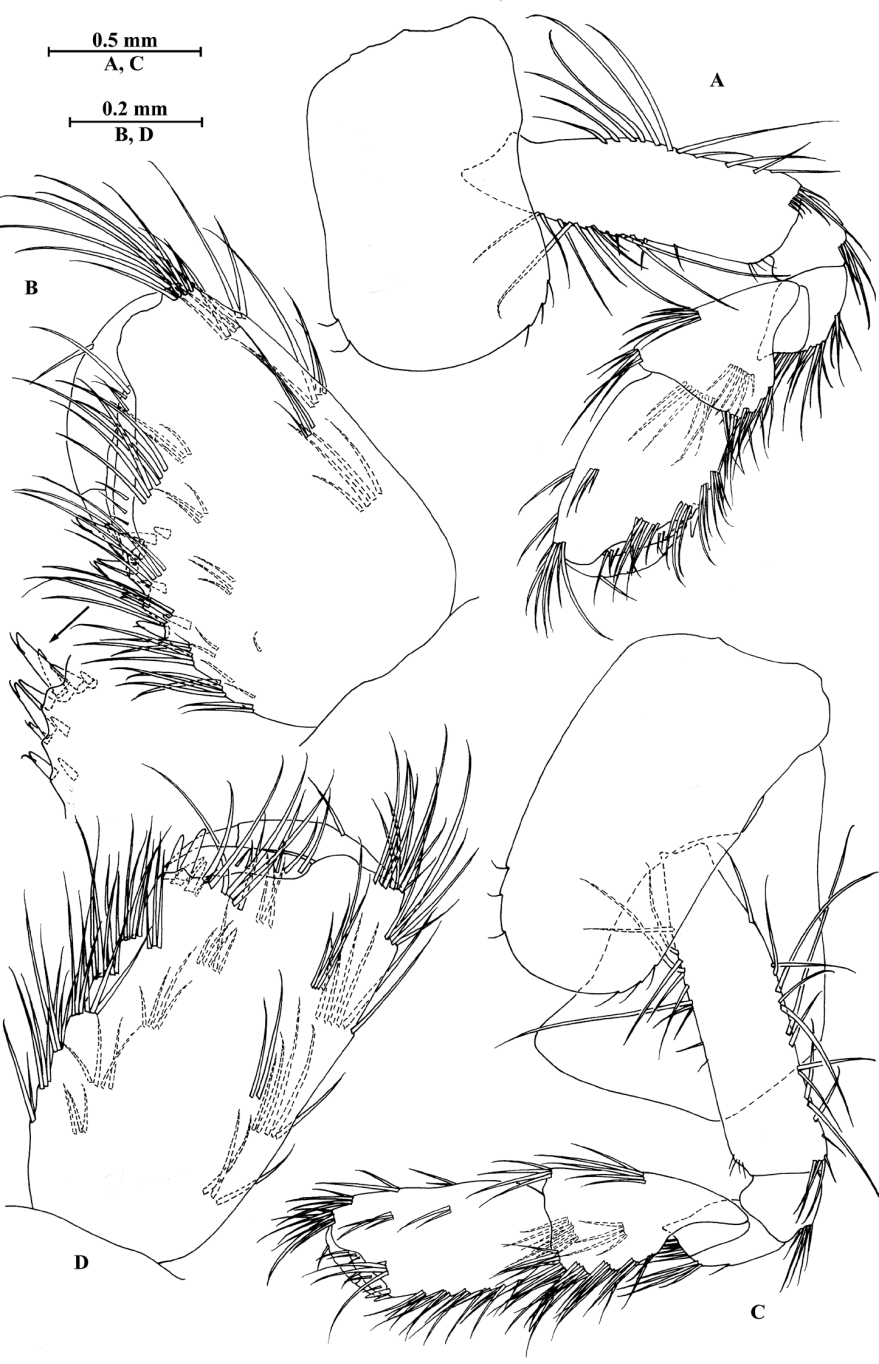
*Gammarus longdong* Hou & Li, sp. n., female paratype. **A** gnathopod I **B** propodus and dactylus of gnathopod I **C** gnathopod II **D** propodus and dactylus of gnathopod II.

**Figure 32.**
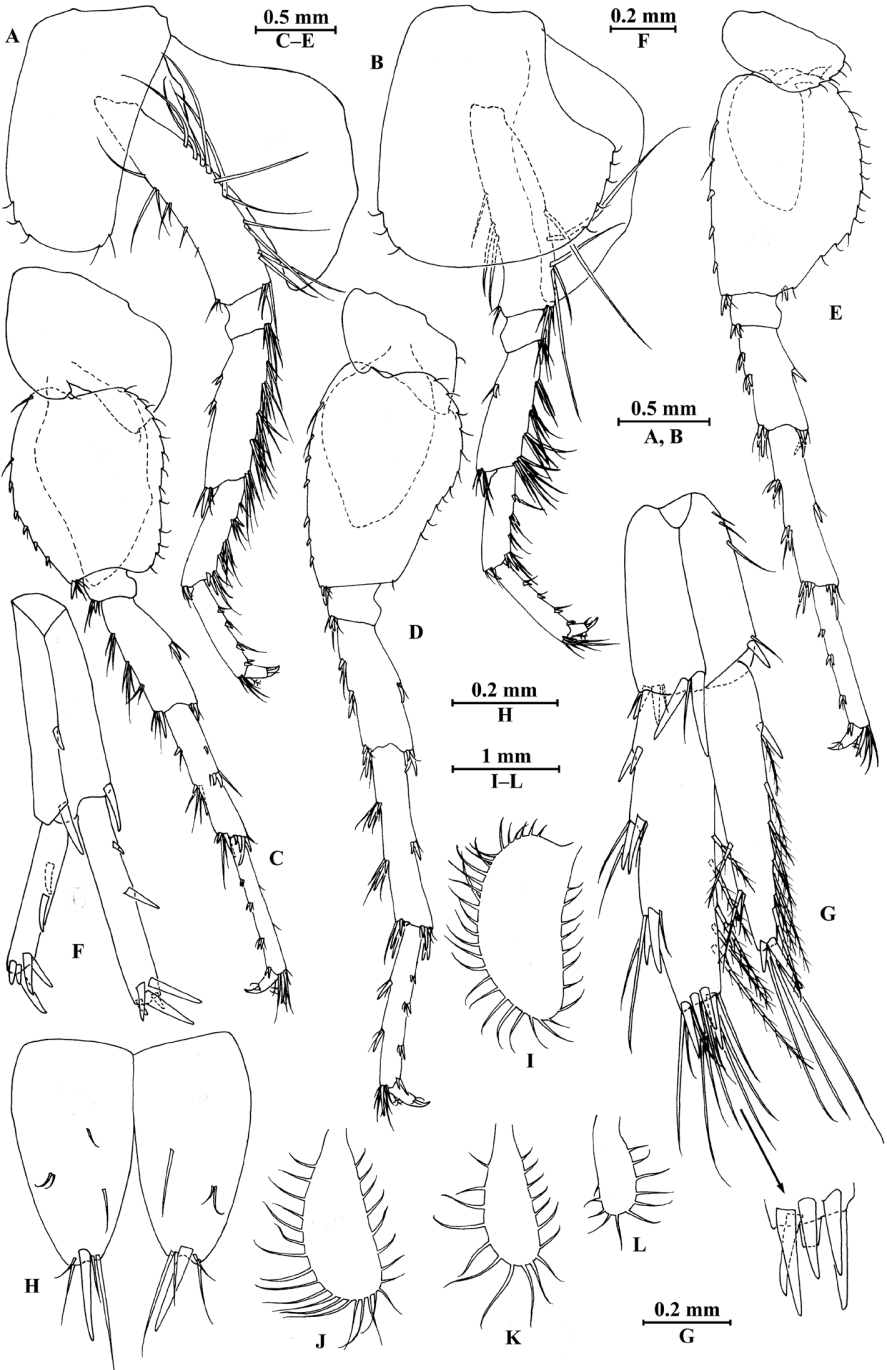
*Gammarus longdong* Hou & Li, sp. n., female paratype. **A** pereopod III **B** pereopod IV **C** pereopod V **D** pereopod VI **E** pereopod VII **F** uropod II **G** uropod III **H** telson **I** oostegite of gnathopod II **J** oostegite of pereopod III **K** oostegite of pereopod IV **L** oostegite of pereopod V.

Figs 27 , 28 , 29 , 30 , 31 , 32

##### Material examined.

Holotype: male (IZCAS-I-A1566-1), 10.1 mm, Qinglong Cave (103.75°E, 27.69°N), altitude 1289 m, Mohan Town, Daguan County, Zhaotong City, Yunnan Province, China, March 18, 2014, collected by Yunchun Li and Jincheng Liu. Paratype: female (IZCAS-I-A1566-2), 7.3 mm, same data as holotype.

##### Etymology.

The species name is taken from the Chinese word, “*longdong*” meaning “Dragon Cave”, referring to a cave filled with water; noun in apposition.

##### Diagnosis.

Peduncle of antenna II with long setae, calceoli absent; merus and carpus of pereopod III with clusters of long setae on posterior margins; epimeral plates II and III with subacute posterodistal corners; uropod I peduncle with no basofacial spine; inner ramus of uropod III reaching 0.9 times the length of outer ramus, terminal article vestigial.

##### Description of holotype male

(IZCAS-I-A1566-1). 10.1 mm.

***Head*** (Fig. 27A): eyes reniform, inferior antennal sinus deep.

*Antenna I* (Fig. 27B, C): peduncle articles I–III in length ratio 1.0: 0.8: 0.4, with distal setae; flagellum with 31 articles, articles V–XXX with aesthetascs; accessory ﬂagellum with four articles; both primary and accessory ﬂagella with short distal setae.

*Antenna II* (Fig. 27D): peduncle articles III–V in length ratio 1.0: 2.9: 2.7, articles IV–V of peduncle with lateral and medial setae; flagellum with 11 articles, each article with long setae; calceoli absent.

*Upper lip* (Fig. 27E): ventral margin rounded, bearing short minute setae.

*Mandible* (Fig. 27G, H): left mandible incisor with five teeth; lacinia mobilis with four teeth; spine row with six pairs of plumose setae; articles I–III of palp in length ratio 1.0: 2.7: 2.0, second article with 15 marginal setae, article III with four A-setae, four B-setae, a row of D-setae, and five E-setae apically; incisor of right mandible with four teeth, lacinia mobilis bifurcate, with small teeth.

*Lower lip* (Fig. 27F): inner lobes lacking, outer lobes covered with thin setae.

*Maxilla I* (Fig. 27I, J): asymmetrical, left inner plate with 15 plumose setae on medial margin; outer plate with 11 robust serrated apical spines; second article of left palp with nine slender spines apically; second article of right palp with four stout spines, one stiff seta and one slender spine.

*Maxilla II* (Fig. 27K): inner plate with 12 plumose setae in an oblique row; inner and outer plates with long setae apically.

*Maxilliped* (Fig. 27L): inner plate with three stout apical spines, one subapical spine, and 20 plumose setae; outer plate bearing a row of 17 blade spines and three plumose setae apically; article IV of palp hooked, with a group of setae at hinge of unguis.

***Pereon.*** *Gnathopod I* (Fig. 28A, B): coxal plate bearing two setae and four setae on anterior and posterior margins, respectively; basis with setae on anterior and posterior margins; merus bearing setae on posterodistal corner; carpus 1.7 times as long as wide, 0.75 times as long as propodus, bearing four clusters of setae along ventral margin and two clusters of setae on dorsal margin; propodus oval, palm with one medial spine and 12 spines on posterior margin and surface; dactylus with one seta on outer margin.

*Gnathopod II* (Fig. 28C, D): coxal plate bearing two setae and three setae on anterior and posterior margins, respectively; basis with setae on anterior and posterior margins; merus bearing setae on posterodistal corner; carpus 1.9 times as long as wide, 0.8 times as long as propodus, bearing six clusters of setae along ventral margin and two clusters of setae on dorsal margin; propodus subrectangular, palm margin with one medial spine and four spines on posterodistal corner; dactylus with one seta on outer margin.

*Pereopod III* (Fig. 29A, B): coxal plate bearing three setae and two setae on anterior and posterior margins, respectively; basis elongated, with setae along anterior and posterior margins; merus with clusters of long setae on posterior margin and one spine on anterior margin, anterodistal corner with one spine accompanied by setae; carpus with two groups of long setae on posterior margin, anterodistal corner with one spine accompanied by setae and posterodistal corner with two spines accompanied by setae; propodus with three spines accompanied by setae on posterior margin and two spines on posterodistal corner; dactylus with one plumose seta on anterior margin, and two setae at hinge of unguis.

*Pereopod IV* (Fig. 29C, D): coxal plate concave, bearing two fine setae on anterior margin and five setae on posterior margin; basis with setae along anterior and posterior margins; merus with four clusters of setae on posterior margin and one spine accompanied by one seta on anterior margin, anterodistal corner with one spine accompanied by setae; carpus and propodus with three or four spines accompanied by setae on posterior margins; dactylus with one plumose seta on anterior margin, and two setae at hinge of unguis.

*Pereopod V* (Fig. 29E, F): coxal plate bearing one seta on anterior and posterior margins, respectively; basis expanded, with two setae and six spines on anterior margin, anterodistal corner with two spines accompanied by setae, posterior margin with a row of 13 setae; merus with two clusters of short setae on anterior margin and one spine accompanied by one seta on posterior margin, anterodistal corner with one spine accompanied by setae and posterodistal corner with two spines accompanied by setae; carpus and propodus with groups of spines accompanied by fine setae on anterior margins; dactylus with one plumose seta on posterior margin, and two setae at hinge of unguis.

*Pereopod VI* (Fig. 29G, H): coxal plate bearing one seta on posterior margin; basis elongated, with two setae and three spines on anterior margin, anterodistal corner with two spines accompanied by setae, posterior margin with a row of nine setae; merus with two spines accompanied by setae on anterior margin and one spine accompanied by one seta on posterior margin, anterodistal and posterodistal corners with two and three spines accompanied by setae respectively; carpus and propodus with three groups of spines accompanied by setae on anterior margins; dactylus with one plumose seta on posterior margin, and two setae at hinge of unguis.

*Pereopod VII* (Figs 29I, J): coxal plate with three setae on posterior margin; basis with two setae and four spines on anterior margin, anterodistal corner with two spines accompanied by setae, posterior margin with a row of 12 setae and one spine; merus with two groups of spines accompanied by setae on anterior margin and one spine accompanied by one seta on posterior margin, anterodistal and posterodistal corners with three and two spines accompanied by one seta, respectively; carpus and propodus with two or three groups of spines on anterior margins; dactylus with one plumose seta on posterior margin, and two setae at hinge of unguis.

*Coxal gill*s: coxal gill of gnathopod II a little shorter than basis; gills of pereopods IV and V longer than bases; gills of pereopods III and VI more than half the length of bases; gill of pereopod VII smallest, less than half of the basis.

***Pleon.*** *Epimeral plates* (Fig. 30A–C): plate I ventrally rounded, bearing eight long setae on anteroventral margin and five setae on posterior margin; plate II with one seta and one spine on ventral margin and seven setae on posterior margin, posterodistal corner subacute; plate III with one seta and two spines on ventral margin and six setae on posterior margin, posterodistal corner subacute.

*Pleopods I–III* (Fig. 30D–F): similar, peduncle with two or three retinacula accompanied by one or two setae; outer ramus slightly shorter than inner ramus, both rami fringed with plumose setae.

***Urosome.*** *Urosomites* (Fig. 28E): urosomite I with one-one-one-one spines accompanied by setae on dorsal margin; urosomite II with one-one-one spines accompanied by setae on dorsal margin; urosomite III with one spine accompanied by two setae on each side.

*Uropods I–III* (Fig. 30G–I): uropod I peduncle with no basofacial spine and outer margin with one spine, inner and outer distal corners with one and two spines respectively; inner ramus with two spines on inner margin; outer ramus with two spines on inner and outer margins each; both rami with five terminal spines. Uropod II short, peduncle bearing one distal spine on each corner; inner ramus with two spines on inner margin; outer ramus with one spine and two spines on inner and outer margins, respectively; both rami with five terminal spines. Uropod III peduncle with three setae on surface and six distal spines; inner ramus 2.2 times as long as peduncle, reaching 0.9 times the length of outer ramus, with one spine accompanied by ten plumose setae and three simple setae on inner margin, with six plumose setae on outer margin and one spine accompanied by long setae distally; proximal article of outer ramus with three clusters of spines accompanied by simple setae on outer margin, with eight plumose setae on inner margin, and four distal spines, terminal article vestigial, with simple setae.

*Telson* (Fig. 29K): deeply cleft, approx. as long as wide, left lobe with two simple setae and two plumose setae on surface and with one distal spine accompanied by three setae; right lobe with one simple seta and two plumose setae on surface and with two distal spines accompanied by two setae.

##### Description of paratype female

(IZCAS-I-A1566-2). 7.3 mm.

***Pereon.*** *Gnathopod I* (Fig. 31A, B): coxal plate bearing two setae on anterior margin and three setae on posterior margin; basis with long setae on anterior and posterior margins; propodus oval, palm with ten spines on posterior margin, bearing long setae along anterior and posterior margins; dactylus with one seta on outer margin.

*Gnathopod II* (Fig. 31C, D): coxal plate bearing three setae on anterior and posterior margins each; basis with setae on anterior and posterior margins; propodus subrectangular, palm margin with four spines on posterodistal corner, bearing long setae along anterior and posterior margins; dactylus with one seta on outer margin.

*Pereopod III* (Fig. 32A): merus and carpus with shorter setae on posterior margins than that of male.

*Pereopods IV–VII* (Fig. 32B–E): similar to those of male.

*Oostegite* (Fig. 32I–L): oostegite of gnathopod II broad, with marginal setae, oostegites of pereopods III and IV elongated, oostegite of pereopod V smallest.

***Urosome.*** *Uropods I–III* (Figs 30J; 32F, G): uropod I peduncle with no basofacial spine and outer margin with three spines, inner and outer distal corners with one and two spines respectively; inner ramus with two spines on inner margin; outer ramus with two spines on inner margin and one spine on outer margin; both rami with five terminal spines. Uropod II short, peduncle bearing one spine on inner margin, each corner with one distal spine; outer ramus with one spine on outer and inner margins each; inner ramus with two spines on inner margin; both rami with five terminal spines. Uropod III peduncle with three setae on surface and five distal spines accompanied by setae; inner ramus 1.5 times as long as peduncle, reaching 0.9 times the length of proximal article of outer ramus, with one spine and plumose setae on inner margin, two plumose setae and one simple seta on outer margin; proximal article of outer ramus with three pairs of spines accompanied by simple setae on outer margin and five plumose setae on inner margin, terminal article much shorter than adjacent spines.

*Telson* (Fig. 32H): cleft, 0.9 times as long as wide, each lobe with simple setae on surface and with one distal spine accompanied by setae.

##### Habitat.

The species was collected in Qinglong Cave Park. The park has a limestone karst mountain landscape. There is an underground river winding through the cave before flowing into a pool. Individuals are found along the bank of river, with no vegetation.

##### Remarks.

The new species of *Gammarus longdong* Hou & Li, sp. n. is similar to *G. craspedotrichus* Hou & Li, 2002 in antenna II with long setae along peduncle margin, calceoli absent; pereopod III merus and carpus with long setae on posterior margins; and uropod I with no basofacial spine. *Gammarus longdong* Hou & Li, sp. n. can be distinguished from *G. craspedotrichus* Hou & Li, 2002 by the following characters (*G. craspedotrichus* in parentheses): urosomites I and II with four groups of spines and setae (with two clusters of spines and setae); and uropod III terminal article vestigial (short but distinct).

The new species of *Gammarus longdong* Hou & Li, sp. n. is similar to *jidutanxian* Hou & Li, sp. n. in antenna II peduncle with long setae, calceoli absent; and uropod III outer ramus with no plumose setae on outer margin. It can be distinguished from *G. jidutanxian* Hou & Li, sp. n. (*G. jidutanxian* in parentheses) in uropod I without basofacial spine (with one basofacial spine); and uropod III inner ramus reaching 0.9 times the length of outer ramus (inner ramus 0.6 times the length of outer ramus).

The new species is similar to *G. egregius* Hou, Li & Li, 2013 in accessory ﬂagellum of antenna I with four articles; antenna II calceoli absent; and uropod I peduncle without basofacial spine. The new species can be distinguished from *G. egregius* Hou, Li & Li, 2013 by the following characters (*G. egregius* in parentheses): urosomite I with one-one-one-one spines accompanied by setae on dorsal margin (bare); urosomite II with one-one-one spines accompanied by setae on dorsal margin (with two single spines); inner ramus of uropod III 0.9 times the length of proximal article of outer ramus (0.6 times the length of outer ramus); and both rami of uropod III with plumose setae on inner margins (simple setae).

The new species is similar to *G. platvoeti* Hou & Li, 2003a in accessory ﬂagellum of antenna I with four articles; antenna II calceoli absent; epimeral plates II and III with subacute posterodistal corners; and uropod I peduncle without basofacial spine. It differs from *G. platvoeti* Hou & Li, 2003a (*G. platvoeti* in parentheses) by merus and carpus of pereopod III with long setae on posterior margins (with a few short setae); urosomites I and II with spines accompanied by setae on dorsal margin (only with setae); inner ramus of uropod III 0.9 times the length of proximal article of outer ramus (0.85 times the length of outer ramus); and both lobes of telson with simple and plumose setae on surface (bare).

#### Gammarus mosuo

Taxon classificationAnimaliaAmphipodaGammaridae

Hou & Li sp. n.

http://zoobank.org/FF370401-2EC9-4E35-B043-11FBFE09FB87

**Figure 33.**
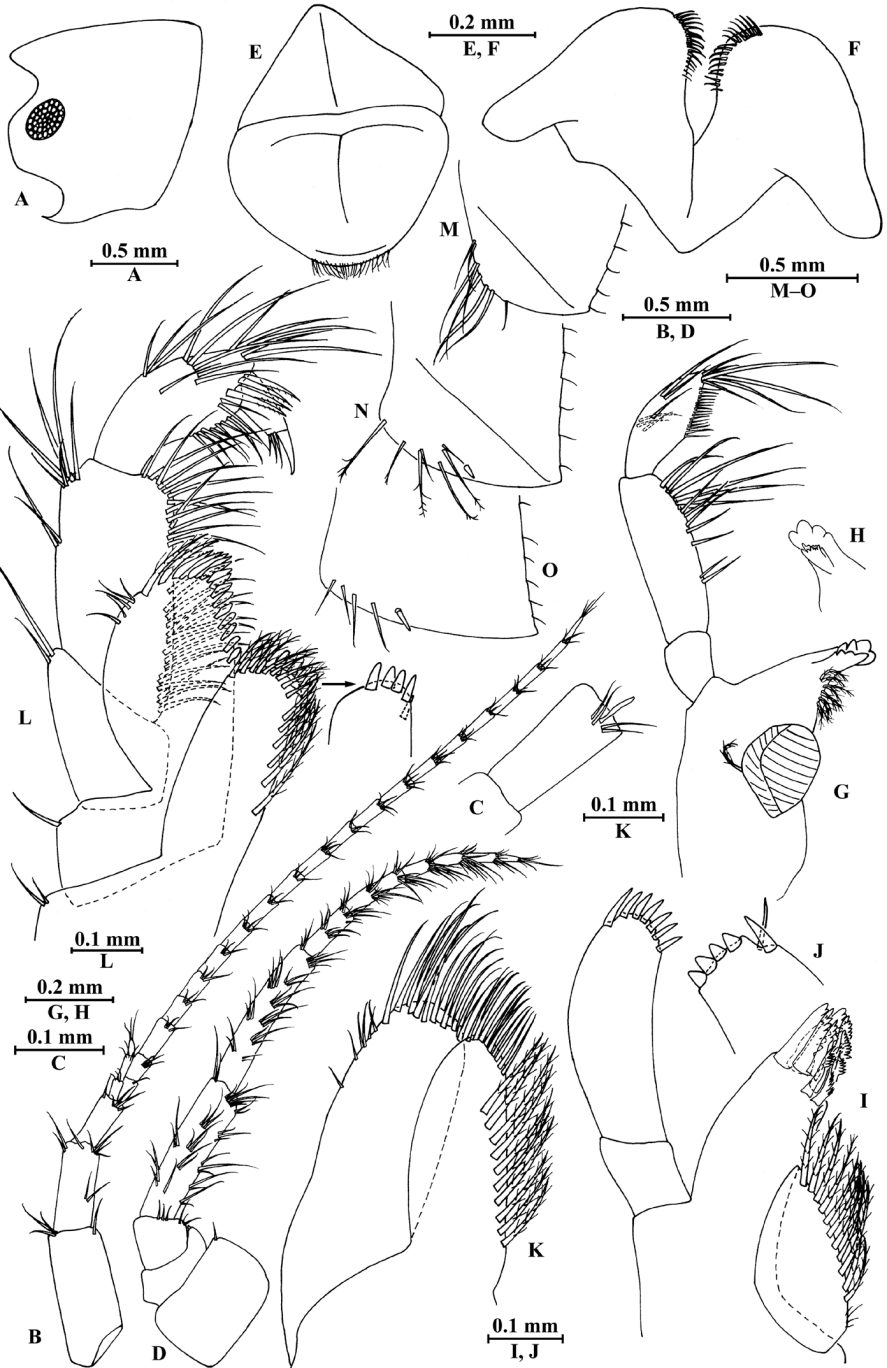
*Gammarus mosuo* Hou & Li, sp. n., male holotype. **A** head **B** antenna I **C** flagellar article of antenna I with aesthetasc **D** antenna II **E** upper lip **F** lower lip **G** left mandible **H** incisor and lacinia mobilis of right mandible **I** left maxilla I **J** distal part of palp article II of right maxilla I **K** maxilla II **L** maxilliped **M** epimeral plate I **N** epimeral plate II **O** epimeral plate III.

**Figure 34.**
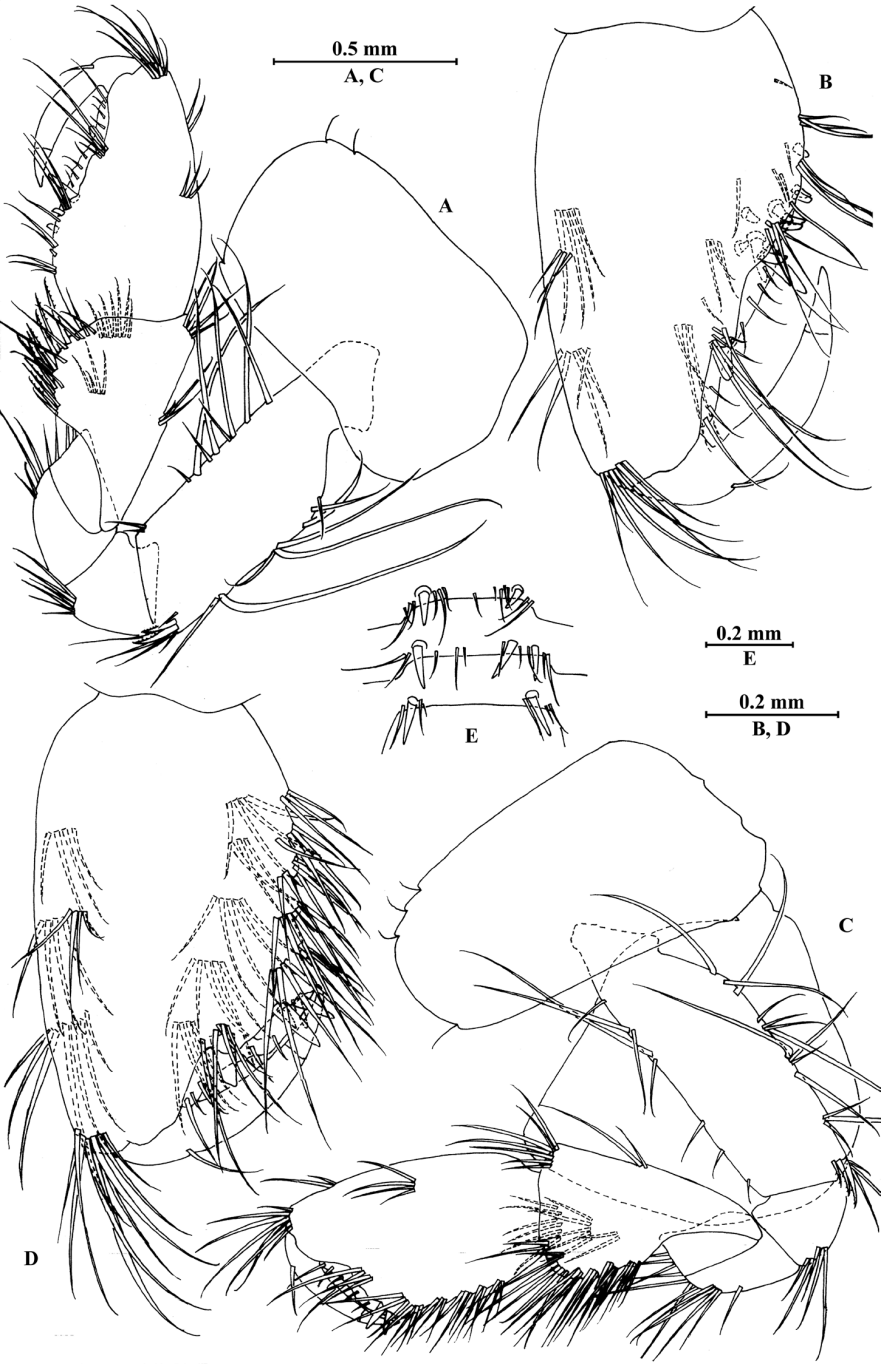
*Gammarus mosuo* Hou & Li, sp. n., male holotype. **A** gnathopod I **B** propodus and dactylus of gnathopod I **C** gnathopod II **D** propodus and dactylus of gnathopod II **E** dorsal margins of urosomites I–III.

**Figure 35.**
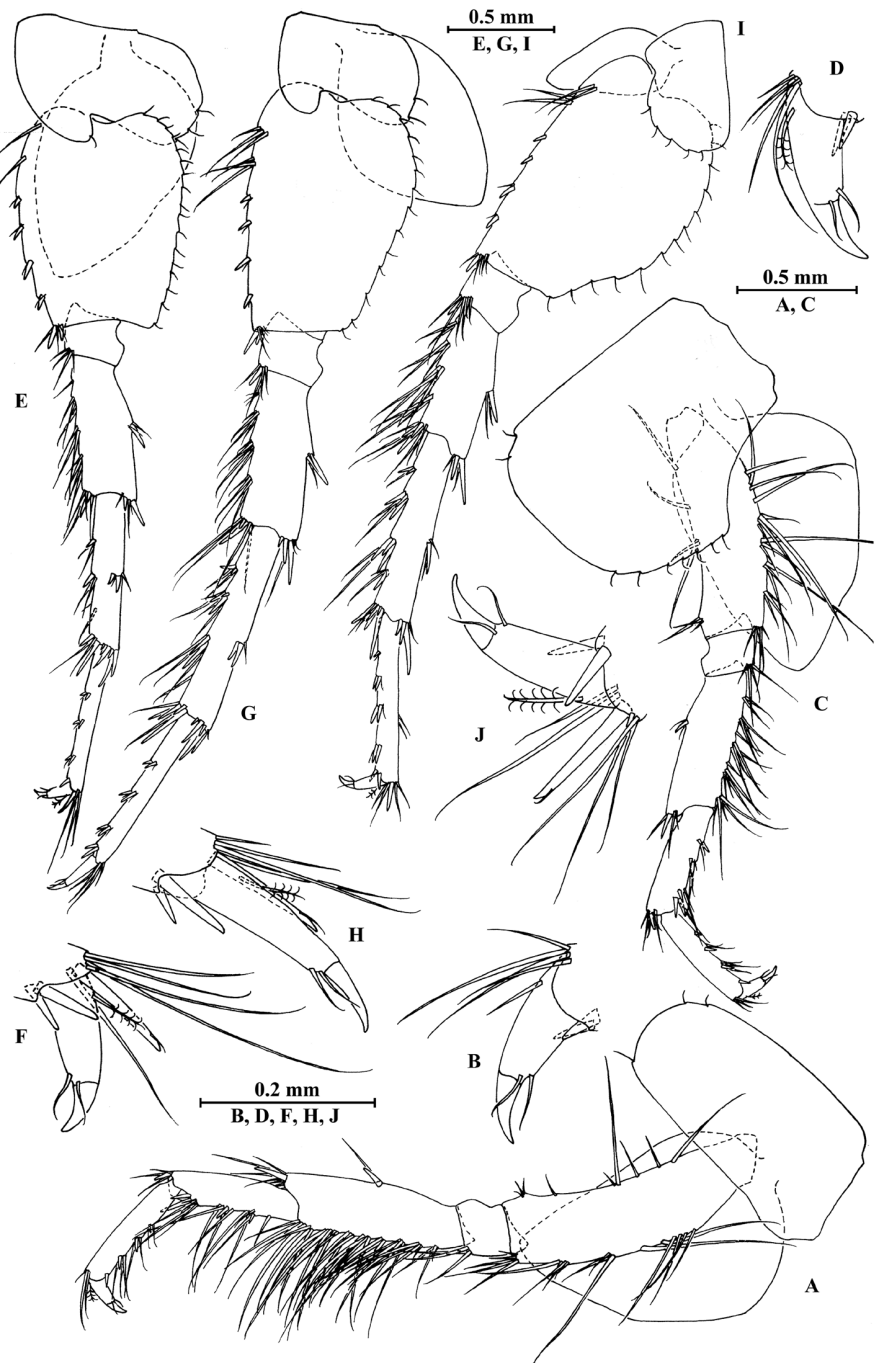
*Gammarus mosuo* Hou & Li, sp. n., male holotype. **A** pereopod III **B** dactylus of pereopod III **C** pereopod IV **D** dactylus of pereopod IV **E** pereopod V **F** dactylus of pereopod V **G** pereopod VI **H** dactylus of pereopod VI **I** pereopod VII **J** dactylus of pereopod VII.

**Figure 36.**
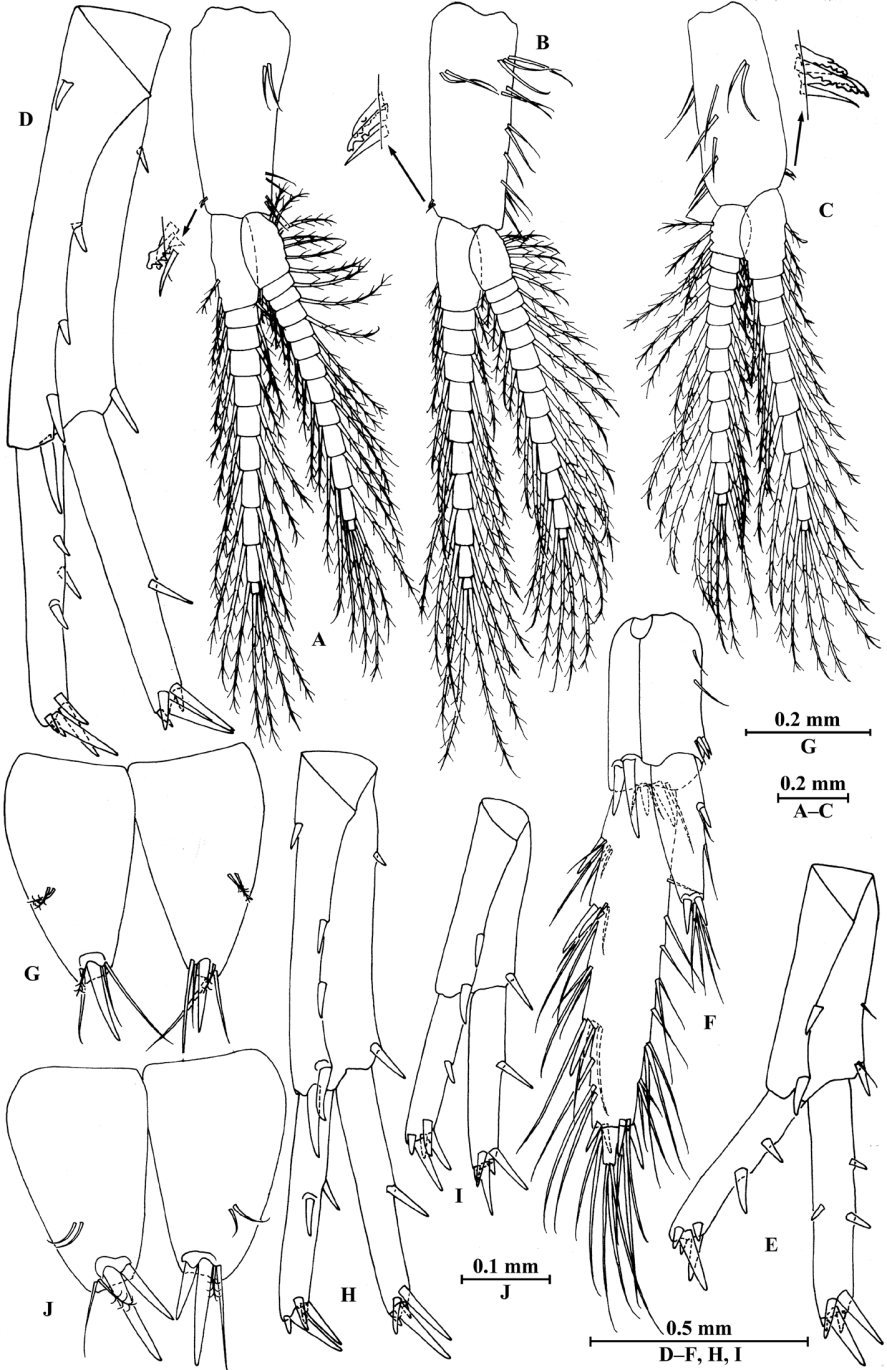
*Gammarus mosuo* Hou & Li, sp. n., **A–G** male, holotype; **H–J** female, paratype. **A** pleopod I **B** pleopod II **C** pleopod III **D** uropod I **E** uropod II **F** uropod III **G** telson **H** uropod I **I** uropod II **J** telson.

**Figure 37.**
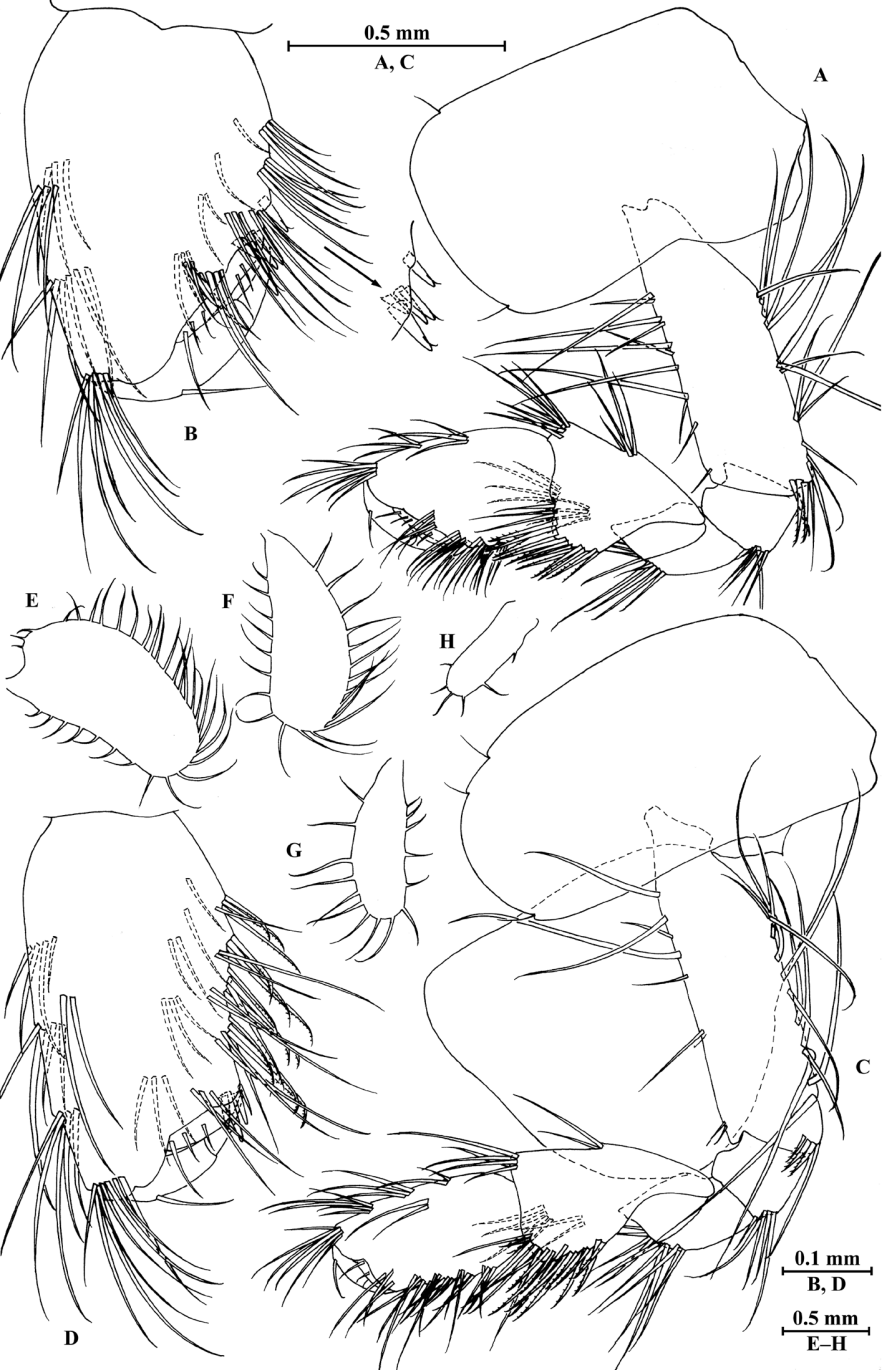
*Gammarus mosuo* Hou & Li, sp. n., female paratype. **A** gnathopod I **B** propodus and dactylus of gnathopod I **C** gnathopod II **D** propodus and dactylus of gnathopod II **E** oostegite of gnathopod II **F** oostegite of pereopod III **G** oostegite of pereopod IV **H** oostegite of pereopod V.

**Figure 38.**
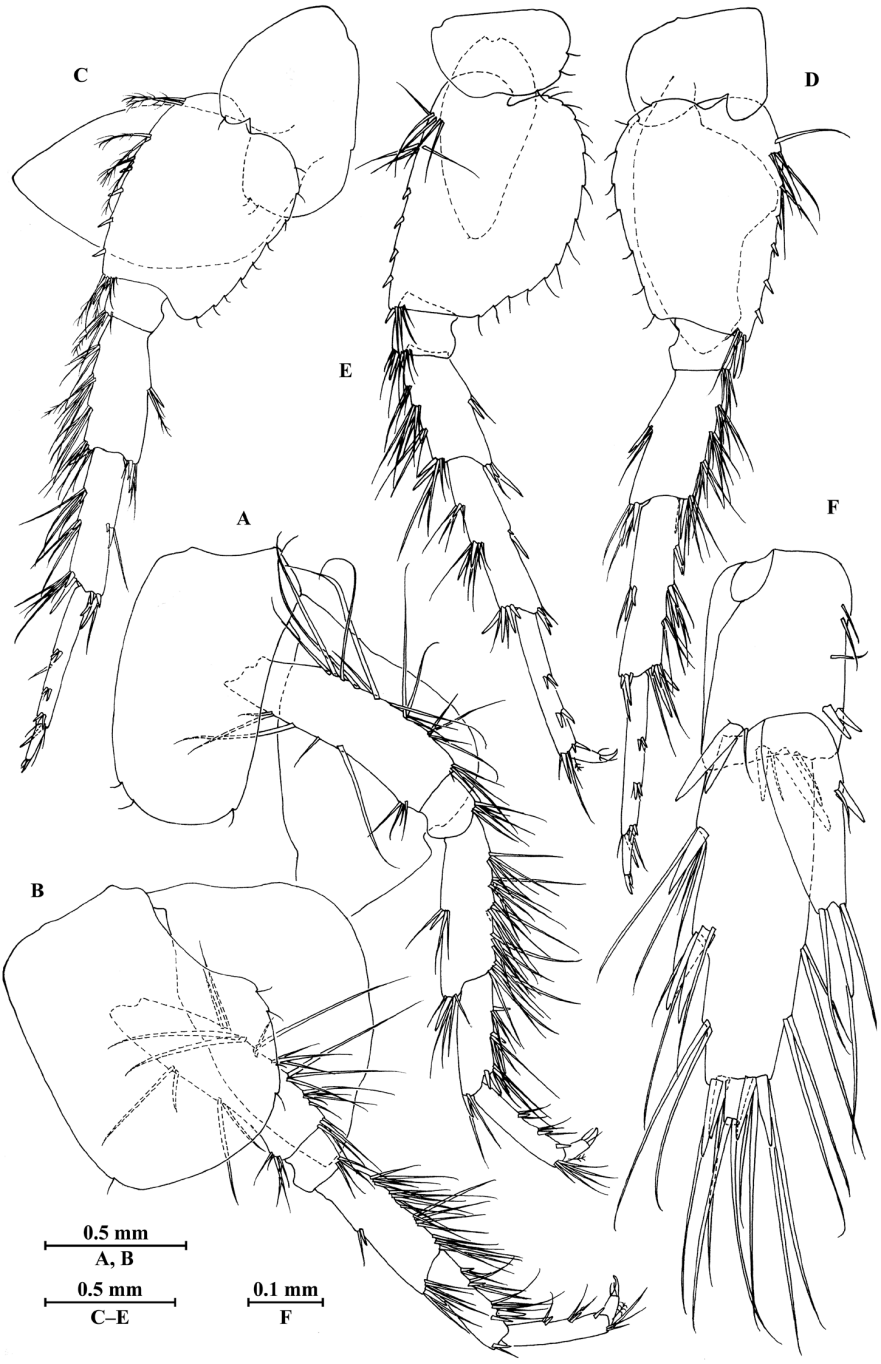
*Gammarus mosuo* Hou & Li, sp. n., female paratype. **A** pereopod III **B** pereopod IV **C** pereopod V **D** pereopod VI (right) **E** pereopod VII **F** uropod III.

Figs 33 , 34 , 35 , 36 , 37 , 38

##### Material examined.

Holotype: male (IZCAS-I-A1570-1), 8.0 mm, Yanyuan County (101.53°E, 27.40°N), altitude 2620 m, Xichang City, Sichuan Province, China, March 23, 2014, collected by Yunchun Li and Jincheng Liu. Paratype: female (IZCAS-I-A1570-2), 6.4 mm, same data as holotype.

##### Etymology.

The name derives from the Mosuo people, living in the type locality; noun in apposition.

##### Diagnosis.

Antenna II calceoli absent; merus to carpus of pereopod III with clusters of long setae on posterior margins; pereopods V–VII with long setae on anterior margins; epimeral plate II with five plumose setae, two simple setae and one spine on ventral margin, posterodistal corner blunt; urosomites with two clusters of spines accompanied by setae on dorsal margins; inner ramus of uropod III reaching 0.4 times the length of outer ramus, both inner and outer rami armed with simple setae.

##### Description of holotype male

(IZCAS-I-A1570-1). 8.0 mm.

***Head*** (Fig. 33A): eyes oval, inferior antennal sinus deep.

*Antenna I* (Fig. 33B, C): peduncle articles I–III in length ratio 1.0: 0.6: 0.4, with distal setae; flagellum with 20 articles, articles III–IX with aesthetascs; accessory ﬂagellum with three articles; both primary and accessory ﬂagella with short distal setae.

*Antenna II* (Fig. 33D): peduncle articles III–V in length ratio 1.0: 2.9: 2.9, articles IV and V with lateral and medial setae; flagellum with seven articles, each article with numerous setae; calceoli absent.

*Upper lip* (Fig. 33E): ventral margin rounded, bearing short minute setae.

*Mandible* (Fig. 33G, H): left mandible incisor with five teeth; lacinia mobilis with four teeth; spine row with five pairs of plumose setae; articles I–III of palp in length ratio 1.0: 2.9: 2.2, second article with 13 marginal setae, article III with three A-setae, three B-setae, a row of D-setae, and six E-setae apically; incisor of right mandible with four teeth, lacinia mobilis bifurcate, with small teeth.

*Lower lip* (Fig. 33F): inner lobes lacking, outer lobes covered with thin setae.

*Maxilla I* (Fig. 33I, J): asymmetrical, left inner plate with 11 plumose setae on medial margin; outer plate with 11 robust serrated apical spines; second article of left palp with seven slender spines apically; second article of right palp with four stout spines, one stiff seta and one slender spine.

*Maxilla II* (Fig. 33K): inner plate with ten plumose facial setae in an oblique row; inner and outer plates with long setae apically.

*Maxilliped* (Fig. 33L): inner plate with four stout apical spines, one subapical spine, and 15 plumose setae; outer plate bearing a row of 14 blade spines and two plumose setae apically; article IV of palp hooked, with three setae at hinge of unguis.

***Pereon.*** *Gnathopod I* (Fig. 34A, B): coxal plate bearing two setae and one seta on anterior and posterior margins, respectively; basis with long setae on anterior and posterior margins; merus bearing setae on posterodistal corner; carpus 1.8 times as long as wide, 0.8 times as long as propodus, bearing clusters of setae along ventral margin and two clusters of setae on dorsal margin; propodus oval, palm with one medial spine and 11 spines on posterior margin and surface; dactylus with one seta on outer margin.

*Gnathopod II* (Fig. 34C, D): coxal plate bearing three setae and one seta on anterior and posterior margins, respectively; basis with setae on anterior and posterior margins; merus bearing setae on posterodistal corner; carpus 1.7 times as long as wide, 0.9 times as long as propodus, bearing five clusters of setae along ventral margin and two clusters of setae on dorsal margin; propodus subrectangular, palm margin with one medial spine and four spines on posterodistal corner; dactylus with one seta on outer margin.

*Pereopod III* (Fig. 35A, B): coxal plate bearing two setae and one seta on anterior and posterior margins, respectively; basis elongated, with setae along anterior and posterior margins; merus with eight clusters of long setae on posterior margin and one spine accompanied by one seta on anterior margin, anterodistal corner with one spine accompanied by setae; carpus with two spines accompanied by groups of long setae on posterior margin, anterodistal corner with one spine accompanied by setae and posterodistal corner with two spines accompanied by setae; propodus with three pairs of spines accompanied by setae on posterior margin and two spines on posterodistal corner; dactylus with one plumose seta on anterior margin, and two setae at hinge of unguis.

*Pereopod IV* (Fig. 35C, D): coxal plate concave, bearing two setae on anterior margin and seven setae on posterior margin; basis with setae along anterior and posterior margins; merus with five clusters of setae on posterior margin and one spine accompanied by two setae on anterior margin, anterodistal corner with one spine accompanied by setae; carpus and propodus with spines accompanied by setae on posterior margins; dactylus with one plumose seta on anterior margin, and two setae at hinge of unguis.

*Pereopod V* (Fig. 35E, F): coxal plate bearing one seta and three setae on anterior and posterior margins, respectively; basis expanded, with two setae and four spines accompanied by setae on anterior margin, anterodistal corner with two spines accompanied by setae, posterior margin with a row of 17 setae; merus with four clusters of setae on anterior margin and one spine accompanied by two setae on posterior margin, anterodistal corner with one spine accompanied by setae and posterodistal corner with two spines accompanied by setae; carpus and propodus with groups of spines accompanied by fine setae on anterior margins; dactylus with one plumose seta on posterior margin, and two setae at hinge of unguis.

*Pereopod VI* (Fig. 35G, H): coxal plate bearing two setae on posterior margin; basis elongated, with two clusters of long setae and four spines accompanied by short setae on anterior margin, anterodistal corner with two spines accompanied by setae, posterior margin with a row of 14 setae; merus with four clusters of setae on anterior margin and one spine accompanied by two setae on posterior margin, anterodistal and posterodistal corners with two and three spines accompanied by setae respectively; carpus with three groups of spines accompanied by straight setae on anterior margin; propodus with three groups of spines accompanied by fine setae on anterior margin; dactylus with one plumose seta on posterior margin, and two setae at hinge of unguis.

*Pereopod VII* (Fig. 35I, J): coxal plate with five setae on posterior margin; basis with two clusters of long setae and four spines accompanied by setae on anterior margin, anterodistal corner with one spine accompanied by setae, posterior margin with a row of 13 setae; merus with four clusters of setae on anterior margin and one spine accompanied by two setae on posterior margin, anterodistal and posterodistal corners with three and two spines accompanied by setae, respectively; carpus with four groups of spines accompanied by straight setae on anterior margin; propodus with groups of spines accompanied by fine setae on anterior margin; dactylus with one plumose seta on posterior margin, and two setae at hinge of unguis.

*Coxal gills*: coxal gill of gnathopod II and gill of pereopod IV a little longer than bases; gills of pereopods III, V and VI shorter than bases; gill of pereopod VII smallest, less than half of the basis.

***Pleon.*** *Epimeral plates* (Fig. 33M–O): plate I ventrally rounded, bearing eight long setae on anteroventral margin and five setae on posterior margin; plate II with five sub-plumose setae, two simple setae and one spine on ventral margin and six setae on posterior margin, posterodistal corner blunt; plate III with four setae and one spine on ventral margin and six setae on posterior margin, posterodistal corner subacute.

*Pleopods I–III* (Fig. 36A–C): similar, peduncle with two or three retinacula accompanied by one seta; outer ramus slightly shorter than inner ramus, both rami fringed with plumose setae.

***Urosome.*** *Urosomites* (Fig. 34E): urosomite I with one spine accompanied by six setae on each side and one seta on dorsal margin; urosomite II with one and two spines accompanied by setae on each side; urosomite III with one spine accompanied by three setae on each side.

*Uropods I–III* (Fig. 36D–F): uropod I peduncle with one basofacial spine, one and three spines on inner and outer margins, respectively, inner and outer distal corners with one spine each; inner ramus with one spine on inner margin; outer ramus with one spine and two spines on inner and outer margins, respectively; both rami with five terminal spines. Uropod II short, peduncle bearing one seta and one spine on inner and outer margins respectively, one distal spine accompanied by one seta on inner corner and one spine on outer corner; inner ramus with two spines on inner margin and one spine on outer margin; outer ramus with two spines on inner margin; both rami with five terminal spines. Uropod III peduncle with two setae on surface and six distal spines; both inner and outer rami armed with simple setae, inner ramus 0.9 times as long as peduncle, reaching 0.4 times the length of outer ramus, with one spine accompanied by two simple setae on inner margin and two spines accompanied by long setae distally; proximal article of outer ramus with three groups of spines accompanied by simple setae on outer margin, simple setae on inner margin, and four distal spines, terminal article with simple setae, slightly shorter than adjacent spines.

*Telson* (Fig. 36G): deeply cleft, 0.9 times as long as wide, each lobe with two plumose setae on surface and with one distal spine accompanied by three or four simple setae and a plumose seta.

##### Description of paratype female

(IZCAS-I-A1570-2). 6.4 mm.

***Pereon.*** *Gnathopod I* (Fig. 37A, B): coxal plate bearing one seta on anterior and posterior margins each; basis with long setae on anterior and posterior margins; propodus oval, palm with five spines on posterior margin, bearing long setae along anterior and posterior margins; dactylus with one seta on outer margin.

*Gnathopod II* (Fig. 37C, D): coxal plate bearing two setae and one seta on anterior and posterior margins, respectively; basis with setae on anterior and posterior margins; propodus subrectangular, palm margin with three spines on posterodistal corner, bearing long setae along anterior and posterior margins; dactylus with one seta on outer margin.

*Pereopod III* (Fig. 38A): similar to that of male.

*Pereopod IV* (Fig. 38B): merus and carpus with longer setae on posterior margins than that of male.

*Pereopod V* (Fig. 38C): similar to that of male; basis to carpus with a few plumose setae on anterior or posterior margins.

*Pereopods VI–VII* (Fig. 38D, E): similar to those of male, but with more setae on anterior margins.

*Oostegite* (Fig. 37E–H): oostegite of gnathopod II broad, with marginal setae, oostegites of pereopods III and IV elongated, oostegite of pereopod V smallest.

***Urosome.*** *Uropods I–III* (Figs 36H, I; 38F): uropod I peduncle with one basofacial spine, one and two spines on inner and outer margins, respectively, inner distal corner with one spine and outer distal corner with two spines; inner ramus with one spine on inner margin; outer ramus with one spine on inner and outer margins each; both rami with five terminal spines. Uropod II short, peduncle bearing one spine on outer margin, each corner with one distal spine; both rami with one spine on inner margin and five terminal spines. Uropod III peduncle with three setae on surface and six distal spines accompanied by setae; both inner and outer rami with simple setae, inner ramus approx. as long as peduncle, reaching 0.6 times the length of outer ramus, with one spine accompanied by one seta on inner margin and one spine accompanied by long setae distally; proximal article of outer ramus with three spines accompanied by simple setae on outer margin and simple setae on inner margin, terminal article slightly shorter than adjacent spines.

*Telson* (Fig. 36J): cleft, 1.2 times as long as wide, each lobe with two simple setae on surface and with two distal spines accompanied by one simple seta and one plumose seta.

##### Habitat.

This species was collected under decomposing leaves alongside a pool.

##### Remarks.

The new species of *Gammarus mosuo* Hou & Li, sp. n. is most similar to *G. sinuolatus* Hou & Li, 2004b in propodus of gnathopod II with long straight setae on anterior margin; pereopods III and IV with long setae on posterior margins; epimeral plates with long setae on ventral margins; and uropod III inner ramus approx. one-third of outer ramus, both rami armed with simple setae. *Gammarus mosuo* Hou & Li, sp. n. can be distinguished from *G. sinuolatus* in the following characters (*G. sinuolatus* in parentheses): antenna II calceoli absent (present); pereopods V–VII with long setae on anterior margin (with few setae on anterior margin); urosomites with two clusters of spines accompanied by setae (four groups of spines accompanied by long setae); and telson with a pair of short facial setae on each lobe (with long setae on dorsal surface).

The new species of *Gammarus mosuo* Hou & Li, sp. n. is similar to *G. curvativus* Hou & Li, 2003b in pereopods III and IV with long straight setae on posterior margins; uropod I with one basofacial spine; and uropod III inner ramus less than half of outer ramus, both rami densely with simple setae. *Gammarus mosuo* Hou & Li, sp. n. differs from *G. curvativus* Hou & Li, 2003b (*G. curvativus* in parentheses) by eyes oval and small (reniform, and relatively large); antenna II calceoli absent (present); gnathopod II propodus with groups of long setae on anterior margin (with long curled setae on anterior margin); pereopods V–VII with long setae along anterior margin (with no long setae); and urosomites with two clusters of spines and setae on dorsal margins (with four groups of spines and setae).

The new species is similar to *G. paucispinus* Hou & Li, 2002b in eyes oval; antenna II calceoli absent; merus and carpus of pereopod III with clusters of long setae on posterior margins; and both rami of uropod III with simple setae. It differs from *G. paucispinus* Hou & Li, 2002b (*G. paucispinus* in parentheses) by urosomite I with two clusters of spines and setae on dorsal margin (with a few short setae); telson 0.9 times as long as wide (0.8 times as long as wide); and each lobe with a pair of setae on surface (with two groups of long setae).

#### Gammarus caecigenus

Taxon classificationAnimaliaAmphipodaGammaridae

Hou & Li sp. n.

http://zoobank.org/C6A67FAB-DC7A-4E2D-8C30-5D7B14A65827

**Figure 39.**
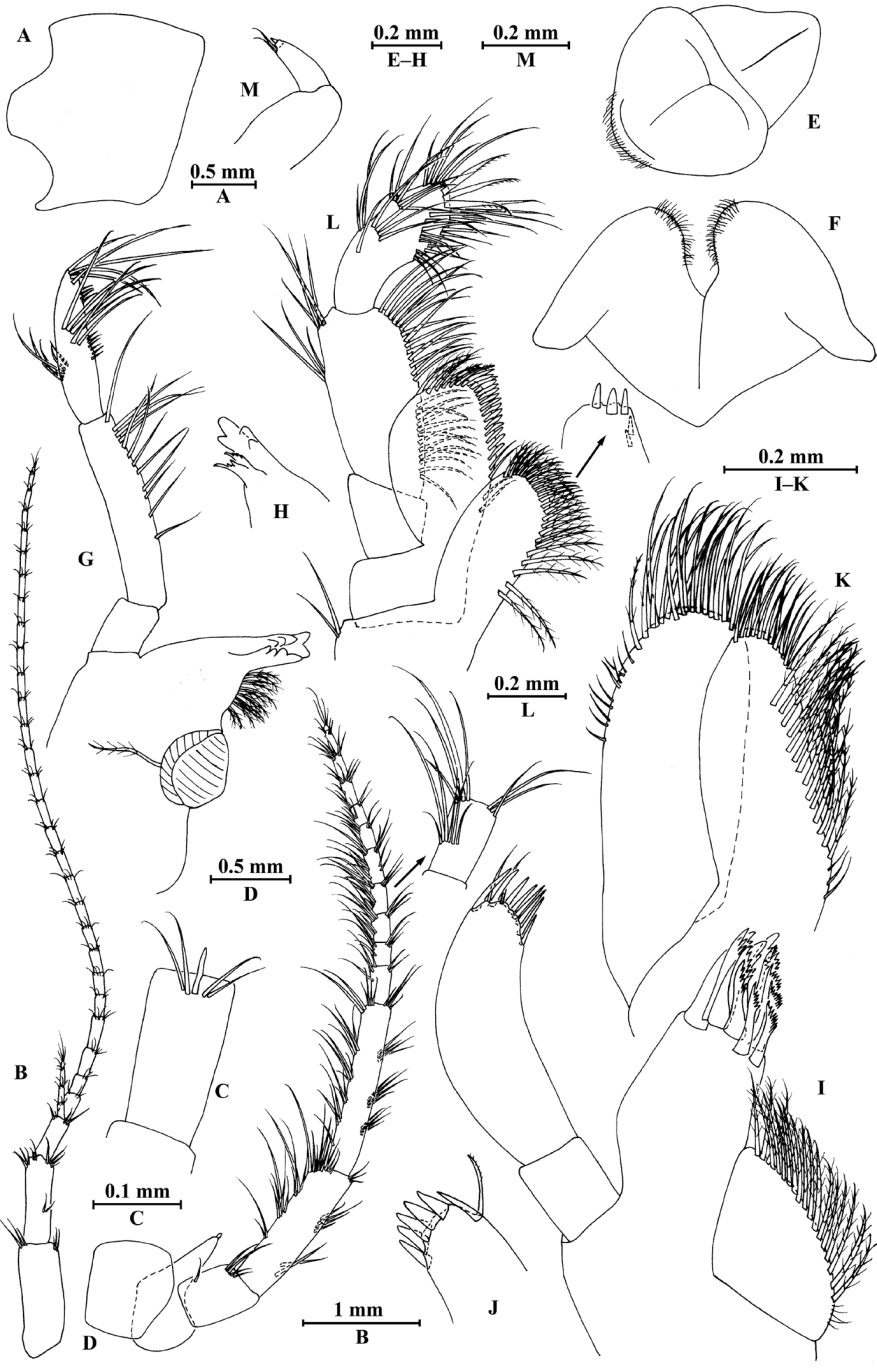
*Gammarus caecigenus* Hou & Li, sp. n., male holotype. **A** head **B** antenna I **C** flagellar article of antenna I with aesthetasc **D** antenna II **E** upper lip **F** lower lip **G** left mandible **H** incisor and lacinia mobilis of right mandible **I** left maxilla I **J** distal part of palp article II of right maxilla I **K** maxilla II **L** maxilliped **M** article IV of maxilliped right palp.

**Figure 40.**
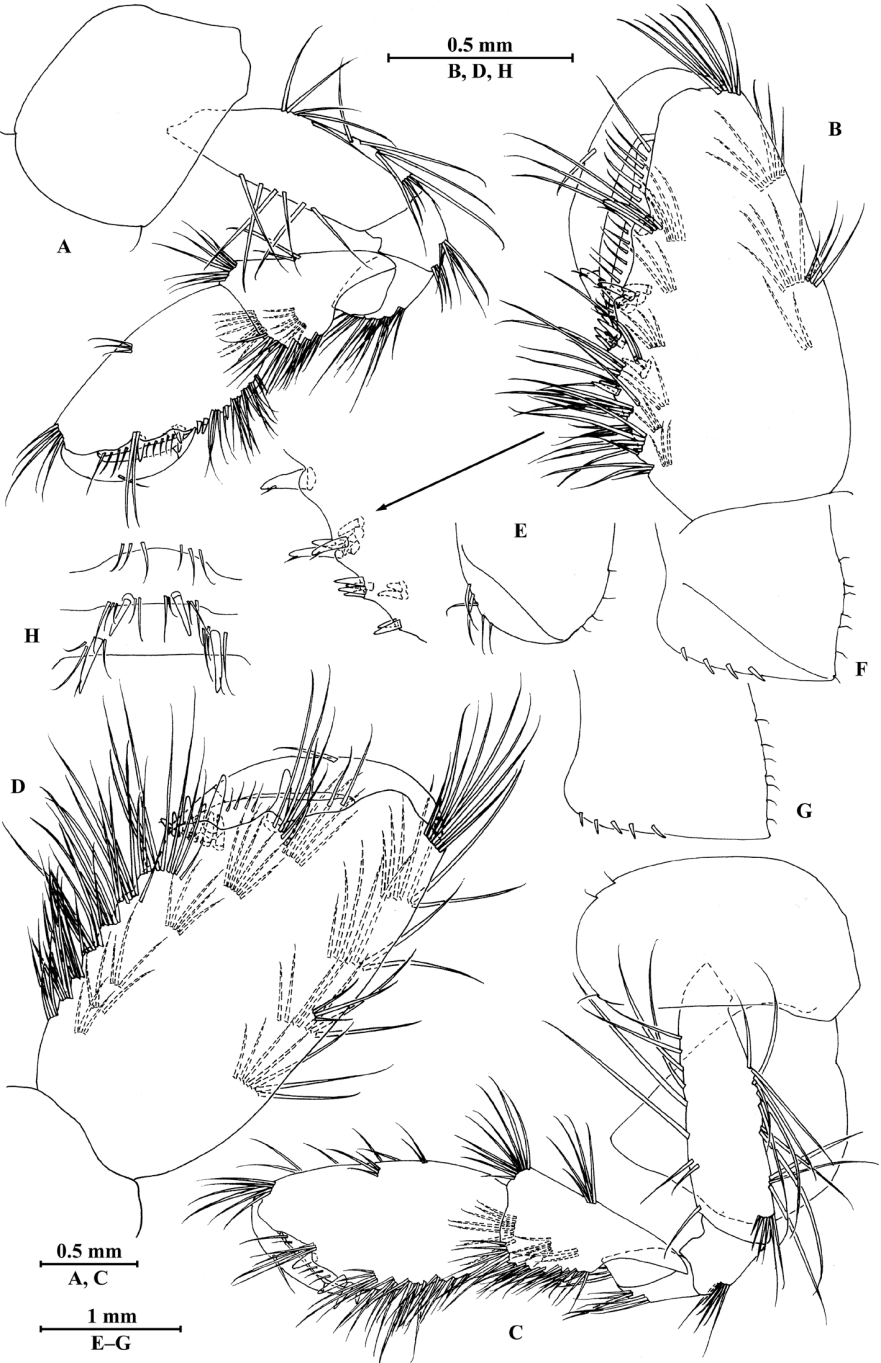
*Gammarus caecigenus* Hou & Li, sp. n., male holotype. **A** gnathopod I **B** propodus and dactylus of gnathopod I **C** gnathopod II **D** propodus and dactylus of gnathopod II **E** epimeral plate I **F** epimeral plate II **G** epimeral plate III **H** dorsal margins of urosomites I–III.

**Figure 41.**
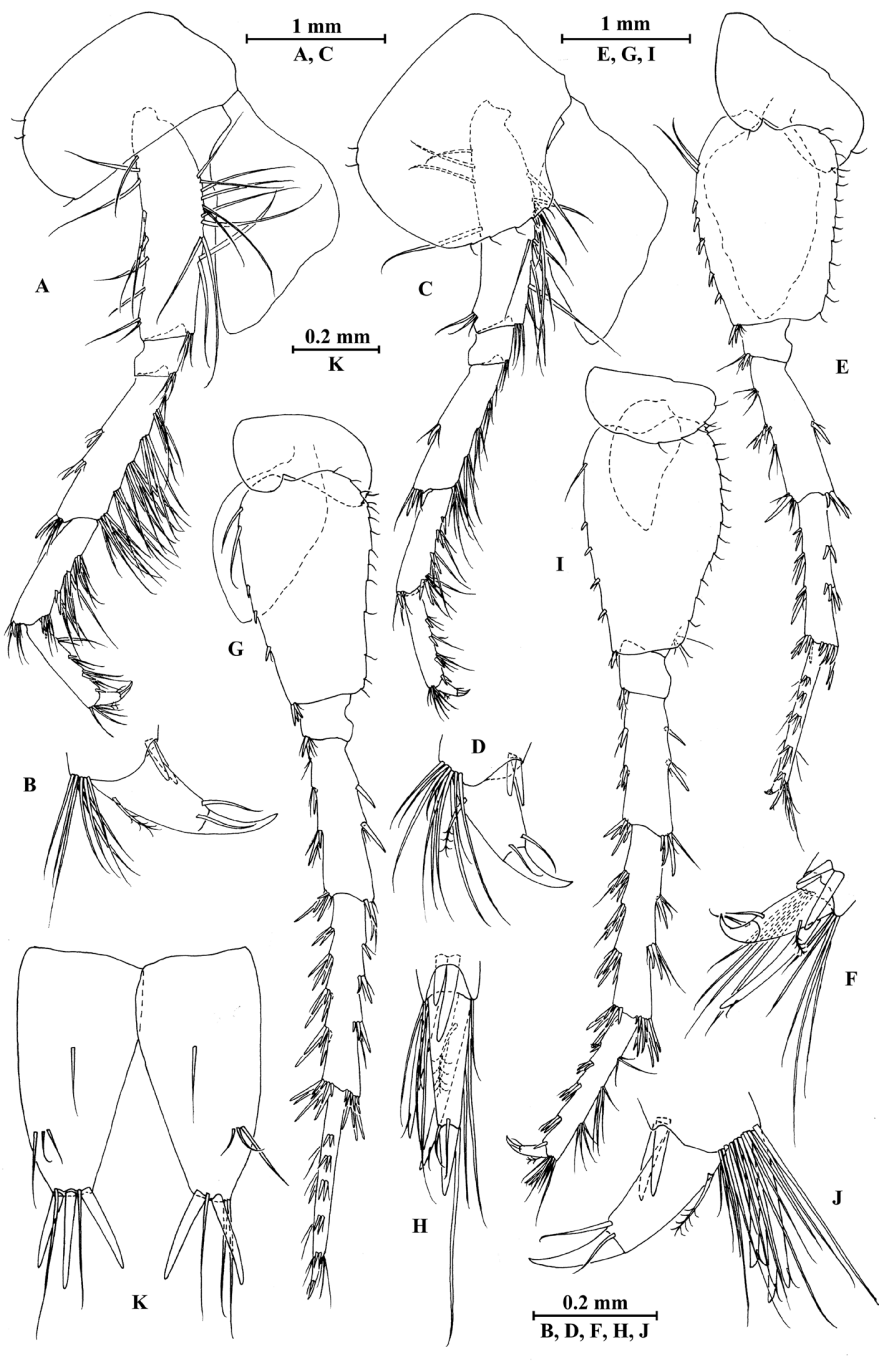
*Gammarus caecigenus* Hou & Li, sp. n., male holotype. **A** pereopod III **B** dactylus of pereopod III **C** pereopod IV **D** dactylus of pereopod IV **E** pereopod V **F** dactylus of pereopod V **G** pereopod VI **H** dactylus of pereopod VI **I** pereopod VII **J** dactylus of pereopod VII **K** telson.

**Figure 42.**
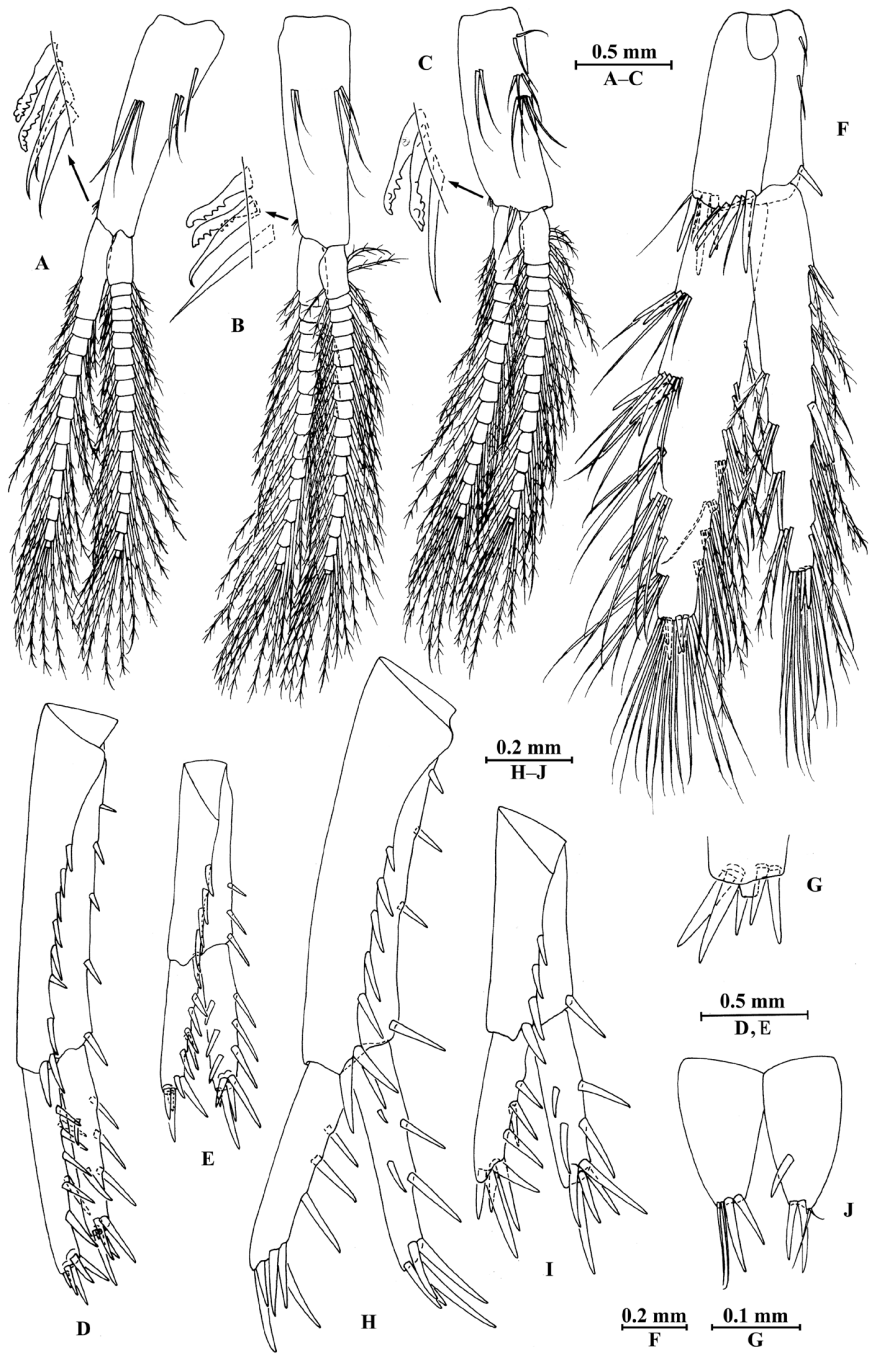
*Gammarus caecigenus* Hou & Li, sp. n., **A–G** male, holotype; **H–J** female, paratype. **A** pleopod I **B** pleopod II **C** pleopod III **D** uropod I **E** uropod II **F** uropod III **G** terminal article of outer ramus (another male) **H** uropod I **I** uropod II **J** telson.

**Figure 43.**
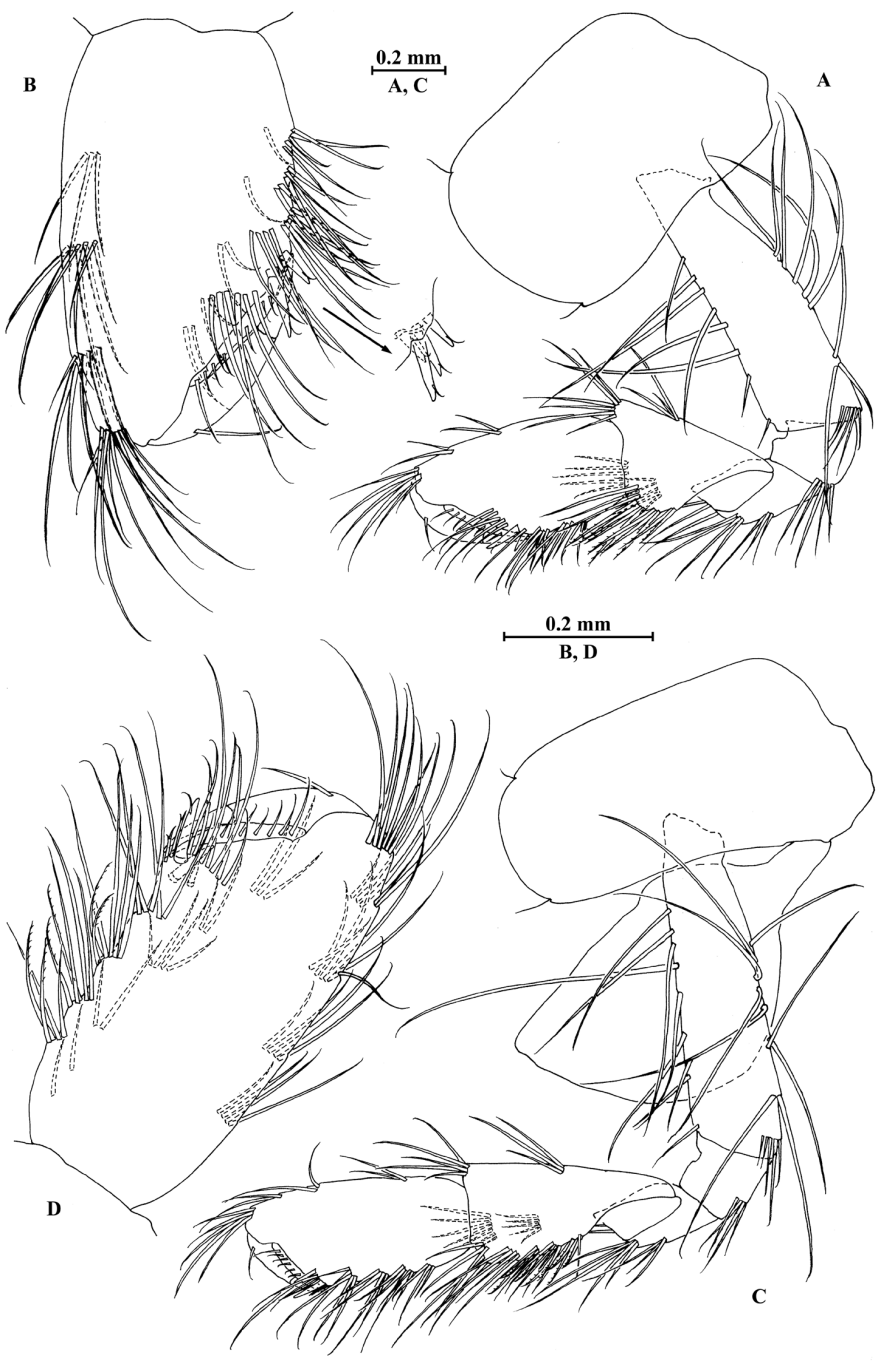
*Gammarus caecigenus* Hou & Li, sp. n., female paratype. **A** gnathopod I **B** propodus and dactylus of gnathopod I **C**, gnathopod II **D** propodus and dactylus of gnathopod II.

**Figure 44.**
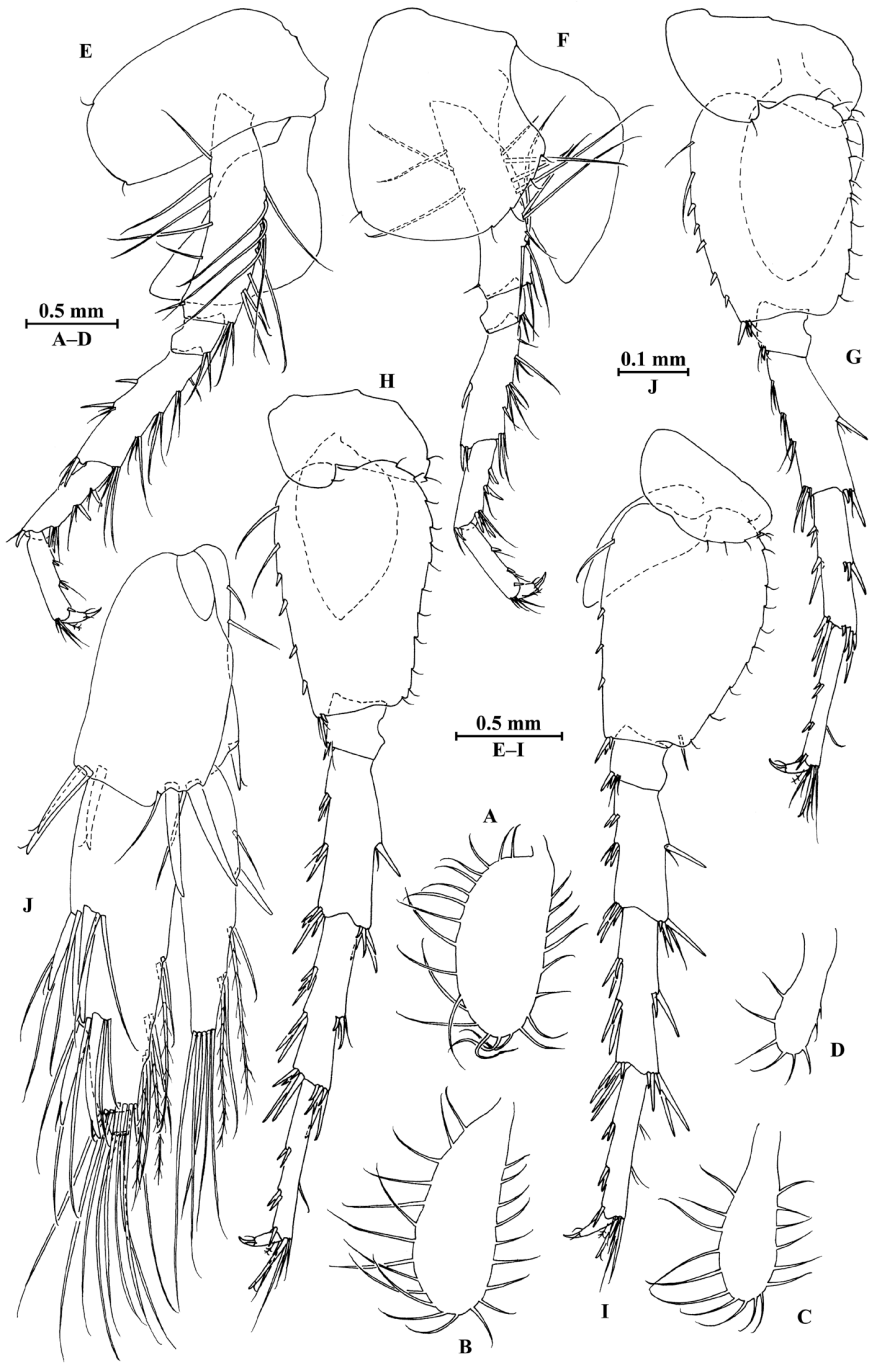
*Gammarus caecigenus* Hou & Li, sp. n., female paratype. **A** oostegite of gnathopod II **B** oostegite of pereopod III **C** oostegite of pereopod IV **D** oostegite of pereopod V **E** pereopod III **F** pereopod IV **G** pereopod V **H** pereopod VI **I** pereopod VII **J** uropod III.

Figs 39 , 40 , 41 , 42 , 43 , 44

##### Material examined.

Holotype: male (IZCAS-I-A1587-1), 10.0 mm, Xingwen County (105.12°E, 28.19°N), altitude 840 m, Yibin City, Sichuan Province, China, April 25, 2014, collected by Yucheng Lin, Huifeng Zhao, Yunchun Li, Jianglang Wu and Fengyuan Li. Paratype: female (IZCAS-I-A1587-2), 5.9 mm, same data as holotype.

##### Etymology.

The epithet derives from the Latin word “*caecigenus*”, referring to the eyes absent; adjective.

##### Diagnosis.

Eyes absent; antenna II with long setae, calceoli absent; merus and carpus of pereopod III with clusters of long setae on posterior margins; armature of urosomites degenerated, urosomite I with setae on dorsal margin, urosomite II with two groups of spines accompanied by setae; uropod I peduncle without basefacial spine; uropods I–II with more marginal spines; inner ramus of uropod III reaching 0.9 times the length of outer ramus, terminal article of outer ramus vestigial.

##### Description of holotype male

(IZCAS-I-A1587-1), 10.0 mm.

***Head*** (Fig. 39A): eyes absent, inferior antennal sinus deep.

*Antenna I* (Fig. 39B, C): peduncle articles I–III in length ratio 1.0: 0.8: 0.4, with distal setae; flagellum with 28 articles, most with aesthetascs; accessory ﬂagellum with four articles; both primary and accessory ﬂagella with short distal setae.

*Antenna II* (Fig. 39D): peduncle articles III–V in length ratio 1.0: 2.1: 2.4, articles IV and V of peduncle with long lateral and medial setae; flagellum with ten articles and one tiny distal article, with setae along dorsal and ventral margins; calceoli absent.

*Upper lip* (Fig. 39E): ventral margin rounded, bearing short minute setae.

*Mandible* (Fig. 39G, H): left mandible incisor with five teeth; lacinia mobilis with four teeth; spine row with seven pairs of plumose setae; articles I–III of palp in length ratio 1.0: 3.1: 2.6, second article with 12 marginal setae, article III with five A-setae, two clusters of B-setae, a row of D-setae, and five E-setae apically; incisor of right mandible with four teeth, lacinia mobilis bifurcate, with small teeth.

*Lower lip* (Fig. 39F): inner lobes lacking, outer lobes covered with thin setae.

*Maxilla I* (Fig. 39I, J): asymmetrical, left inner plate with 18 plumose setae and five fine setae on medial margin; outer plate with 11 robust serrated apical spines; second article of left palp with two simple setae and eight slender spines apically; second article of right palp with five stout spines, one stiff seta and one slender spine.

*Maxilla II* (Fig. 39K): inner plate with three simple setae and 14 plumose facial setae in an oblique row; inner and outer plates with long setae apically.

*Maxilliped* (Fig. 39L, M): inner plate with three stout apical spines, two subapical spines, and 25 plumose setae; outer plate bearing four simple setae, a row of 16 blade spines and five plumose setae apically; article IV of left palp missing, right palp hooked, with two setae at hinge of unguis.

***Pereon.*** *Gnathopod I* (Fig. 40A, B): coxal plate bearing one seta on anterior and posterior margins each; basis with long setae on anterior and posterior margins; merus bearing setae on posterodistal corner; carpus 1.6 times as long as wide, 0.8 times as long as propodus, bearing four clusters of setae along ventral margin and two clusters of setae on dorsal margin; propodus oval, palm with one medial spine and 13 spines on posterior margin and surface; dactylus with one seta on outer margin.

*Gnathopod II* (Fig. 40C, D): coxal plate bearing two setae and one seta on anterior and posterior margins, respectively; basis with setae on anterior and posterior margins; merus bearing setae on posterodistal corner; carpus 2.2 times as long as wide, 0.8 times as long as propodus, bearing eight clusters of setae along ventral margin and two clusters of setae on dorsal margin; propodus subrectangular, palm margin with one medial spine and seven spines on posterodistal corner; dactylus with one seta on outer margin.

*Pereopod III* (Fig. 41A, B): coxal plate bearing two setae and one seta on anterior and posterior margins, respectively; basis elongated, with setae along anterior and posterior margins; merus with two short setae and five clusters of long straight setae on posterior margin and two groups of spines accompanied by setae on anterior margin, anterodistal corner with one spine accompanied by setae; carpus with three groups of spines accompanied by long setae on posterior margin, anterodistal corner with one spine accompanied by setae and posterodistal corner with two spines accompanied by setae; propodus with two spines accompanied by setae on posterior margin and two spines on posterodistal corner; dactylus with one plumose seta on anterior margin, and two setae at hinge of unguis.

*Pereopod IV* (Fig. 41C, D): coxal plate concave, bearing two setae on anterior margin and five setae on posterior margin; basis with setae along anterior and posterior margins; merus with a single seta and four clusters of straight setae on posterior margin and one spine accompanied by two setae on anterior margin, anterodistal corner with two spines accompanied by setae; carpus and propodus with spines accompanied by setae on posterior margins; dactylus with one plumose seta on anterior margin, and two setae at hinge of unguis.

*Pereopod V* (Fig. 41E, F): coxal plate bearing one seta and two setae on anterior and posterior margins, respectively; basis expanded, with two setae and five spines accompanied by setae on anterior margin, anterodistal corner with two spines accompanied by setae, posterior margin with a row of 13 setae; merus with one spine accompanied by clusters of short setae on anterior margin and one spine accompanied by two setae on posterior margin, anterodistal corner with one spine accompanied by setae and posterodistal corner with two spines accompanied by setae; carpus and propodus with three to four groups of spines accompanied by fine setae on anterior margins; dactylus with one plumose seta on posterior margin, and two setae at hinge of unguis.

*Pereopod VI* (Fig. 41G, H): coxal plate bearing two setae on posterior margin; basis elongated, with two long setae and three spines accompanied by setae on anterior margin, anterodistal corner with three spines accompanied by one seta, posterior margin with a row of 13 setae; merus with three spines accompanied by setae on anterior margin and two spines accompanied by two setae on posterior margin, anterodistal and posterodistal corners with four and three spines accompanied by setae respectively; carpus with groups of spines accompanied by setae on anterior and posterior margins; propodus with groups of spines accompanied by fine setae on anterior margin; dactylus with one plumose seta on posterior margin, and two setae at hinge of unguis.

*Pereopod VII* (Fig. 41I, J): coxal plate with five setae on posterior margin; basis with one seta and four spines accompanied by setae on anterior margin, anterodistal corner with one spine accompanied by setae, posterior margin with a row of 15 setae; merus with three spines accompanied by setae on anterior margin and two spines accompanied by two setae on posterior margin, anterodistal and posterodistal corners with five and three spines accompanied by setae, respectively; carpus with groups of spines accompanied by setae on anterior and posterior margins, respectively; propodus with groups of spines accompanied by fine setae on anterior margin and three clusters of long setae on posterior margin; dactylus with one plumose seta on posterior margin, and two setae at hinge of unguis.

*Coxal gills*: coxal gill of gnathopod II and gill of pereopod V a little shorter than bases; gill of pereopod III approx. as long as basis; gill of pereopod IV a little longer than basis; gill of pereopod VI approx. half of the basis; gill of pereopod VII smallest, less than half of the basis.

***Pleon.*** *Epimeral plates* (Fig. 40E–G): plate I ventrally rounded, bearing five long setae on anteroventral margin and four setae on posterior margin; plate II with four spines on ventral margin and seven setae on posterior margin, posterodistal corner blunt; plate III with five spines on ventral margin and seven setae on posterior margin, posterodistal corner blunt.

*Pleopods I–III* (Fig. 42A–C): similar, peduncle with two retinacula accompanied by one to three setae; outer ramus approx. as long as inner ramus, both rami fringed with plumose setae.

***Urosome.*** *Urosomites* (Fig. 40H): urosomite I with two clusters of dorsal setae; urosomite II with one spine accompanied by five setae on each side; urosomite III with one spine accompanied by three setae on each side.

*Uropods I–III* (Fig. 42D–F): uropod I peduncle longer than rami, without basofacial spine, with four and seven spines on inner and outer margins, respectively, inner and outer distal corners with one spine and two spines respectively; inner ramus with three spines on inner margin and two spines on outer margin; outer ramus with two spines and five spines on inner and outer margins, respectively; both rami with five terminal spines. Uropod II short, peduncle a little longer than rami, bearing two spines on inner margin, four spines on outer margin and one distal spine on each corner; inner ramus with three spines on inner and outer margins each; outer ramus with one spine on inner margin and four spines on outer margin; both rami with five terminal spines. Uropod III peduncle with two setae on surface and seven distal spines; inner ramus 2.0 times as long as peduncle, reaching 0.9 times the length of outer ramus, with two spines accompanied by simple and plumose setae on inner margin, four plumose setae accompanied by simple setae on outer margin and two spines accompanied by long setae distally; proximal article of outer ramus with three groups of spines accompanied by simple setae on outer margin, simple and plumose setae on inner margin, and four distal spines, terminal article vestigial.

*Telson* (Fig. 41K): deeply cleft, 1.1 times as long as wide, each lobe with four setae on surface; left lobe with three distal spines accompanied by two simple setae and right lobe with two distal spines accompanied by three simple setae.

##### Description of paratype female

(IZCAS-I-A1587-2). 5.9 mm.

***Pereon.*** *Gnathopod I* (Fig. 43A, B): coxal plate bearing one seta on anterior and posterior margins each; basis with long setae on anterior and posterior margins; propodus oval, palm with four spines on posterior margin, bearing long setae along anterior and posterior margins; dactylus with one seta on outer margin.

*Gnathopod II* (Fig. 43C, D): coxal plate bearing one seta on anterior and posterior margins each; basis with setae on anterior and posterior margins; propodus subrectangular, palm margin with three spines on posterodistal corner, bearing long setae along anterior and posterior margins; dactylus with one seta on outer margin.

*Pereopods III and IV* (Fig. 44E, F): merus and carpus with fewer setae on posterior margins than those of male.

*Pereopods V–VII* (Fig. 44G–I): similar to those of male.

*Oostegite* (Fig. 44A–D): oostegite of gnathopod II broad, with marginal setae, oostegites of pereopods III and IV elongated, oostegite of pereopod V smallest.

***Urosome.*** *Uropods I–III* (Figs 42H, I; 44J): uropod I peduncle longer than rami, without basofacial spine, with three and five spines on inner and outer margins, respectively, inner distal corner with one spine and outer distal corner with three spines; inner ramus with two spines on inner and outer margins each; outer ramus with three spines on inner margin; both rami with five terminal spines. Uropod II short, peduncle a little longer than rami, bearing three spines on outer margin, each corner with one distal spine; inner ramus with two spines on inner and outer margins each; outer ramus with one spine on inner margin and three spines on outer margin; both rami with five terminal spines. Uropod III peduncle with two setae on surface and six distal spines accompanied by setae; inner ramus 1.1 times as long as peduncle, reaching 0.7 times the length of outer ramus, with one spine accompanied by three simple setae and two plumose setae on inner margin; proximal article of outer ramus with two pairs of spines accompanied by simple setae on outer margin and three plumose setae accompanied by three simple setae on inner margin, terminal article much shorter than distal setae; adjacent spine absent.

*Telson* (Fig. 42J): cleft, 1.1 times as long as wide, right lobe with one spine on surface, each lobe with two distal spines accompanied by two setae.

##### Variability.

Outer ramus of uropod III without terminal article or much shorter than adjacent spines.

##### Habitat.

This species was collected from a cave, where a pool with an area of one square meter was formed by dripping water from stalactites.

##### Remarks.

The new species of *Gammarus caecigenus* Hou & Li, sp. n. is most similar to *G. hirtellus* Hou, Li & Li, 2013 in eyes absent; epimeral plate III posterodistal corner subacute; and uropod III inner ramus 0.9 times the length of outer ramus, terminal article vestigial. *Gammarus caecigenus* Hou & Li, sp. n. differs from *G. hirtellus* Hou, Li & Li, 2013 (*G. hirtellus* in parentheses) in antenna II calceoli absent (present); pereopod III with long straight setae on posterior margin (with long curled setae); pereopods V–VII with groups of spines on anterior margins, but with few setae (with spines accompanied by long setae); uropods I and II with more spines along peduncle and both rami (with one spine on each side of inner and outer rami); and urosomites I and II with two groups of spines and setae (with four groups of spines and setae).

The comparison between these seven species is presented in the following key.

### Key to the new species of *Gammarus* from southern China

**Table:** 

1	Eyes present	**2**
–	Eyes absent	***G. caecigenus* Hou & Li, sp. n.**
2	Uropod III inner ramus reaching 0.9 times the length of outer ramus	***G. longdong* Hou & Li, sp. n.**
–	Uropod III inner ramus less than 0.6 times the length of outer ramus	**3**
3	Pereopods V–VII with many long setae on anterior margins	***G. mosuo* Hou & Li, sp. n.**
–	Pereopods V–VII with a few short setae on anterior margins	**4**
4	Uropod III both margins of inner and outer rami with plumose setae	***G. qinling* Hou & Li, sp. n.**
–	Uropod III outer margin of outer ramus with no plumose setae	**5**
5	Antenna II peduncular articles IV and V with long setae, terminal article of uropod III shorter than adjacent spines	***G. jidutanxian* Hou & Li, sp. n.**
–	Antenna II peduncular articles IV and V with short setae, terminal article of uropod III subequal or longer than adjacent spines	**6**
6	Antenna II calceoli present in male, inner margin of outer ramus in uropod III with a row of ten plumose setae	***G. zhigangi* Hou & Li, sp. n.**
–	Antenna II calceoli absent, inner margin of outer ramus in uropod III with three or four plumose setae	***G. vallecula* Hou & Li, sp. n.**

## Supplementary Material

XML Treatment for Gammarus

XML Treatment for Gammarus vallecula

XML Treatment for Gammarus qinling

XML Treatment for Gammarus zhigangi

XML Treatment for Gammarus jidutanxian

XML Treatment for Gammarus longdong

XML Treatment for Gammarus mosuo

XML Treatment for Gammarus caecigenus
